# Stimuli-controlled self-assembly of diverse tubular aggregates from one single small monomer

**DOI:** 10.1038/ncomms14943

**Published:** 2017-04-12

**Authors:** Qixun Shi, Tomas Javorskis, Karl-Erik Bergquist, Artūras Ulčinas, Gediminas Niaura, Ieva Matulaitienė, Edvinas Orentas, Kenneth Wärnmark

**Affiliations:** 1 Center for Analysis and Synthesis, Department of Chemistry, Lund University, Lund SE-221 00, Sweden; 2 Institute of Advanced Synthesis, School of Chemistry and Molecular Engineering, Jiangsu National Synergetic Innovation Center for Advanced Materials, Nanjing Tech University, Nanjing 211816, China; 3 Department of Organic Chemistry, Vilnius University, Vilnius LT-03225, Lithuania; 4 Department of Nanoengineering, Center for Physical Sciences and Technology, Vilnius LT-02300, Lithuania; 5 Department of Organic Chemistry, Center for Physical Sciences and Technology, Vilnius LT-10257, Lithuania

## Abstract

The design and synthesis of new stimuli-responsive hydrogen-bonding monomers that display a diversity of self-assembly pathways is of central importance in supramolecular chemistry. Here we describe the aggregation properties of a simple, intrinsically *C*_2_-symmetric enantiopure bicyclic cavity compound bearing a terminally unsubstituted ureidopyrimidinone fragment fused with a pyrrole moiety in different solvents and in the absence and presence of C_60_ and C_70_ guests. The tetrameric cyclic aggregate is selectively obtained in chlorinated solvents, where only part of the available hydrogen bonding sites are utilized, whereas in toluene or upon addition of C_70_ guests, further aggregation into tubular supramolecular polymers is achieved. The open-end cyclic assemblies rearrange into a closed-shell capsule upon introduction of C_60_ with an accompanied symmetry breaking of the monomer. Our study demonstrates that a C_60_ switch can be used to simultaneously control the topology and occupancy of tubular assemblies resulting from the aggregation of small monomers.

## Abstract (web summary)

Hydrogen bonds are powerful supramolecular motifs, owing to their selective and dynamic nature. Here, the authors build orthogonal hydrogen-bonding sites into a single molecule, allowing it to form diverse hierarchical assemblies and exhibit self-sorting behaviour in response to certain stimuli.

The careful design of the shape of the monomer and the relative position of the hydrogen-bonding (H-bonding) motifs are prerequisites for the construction of supramolecular H-bonded assemblies that possess a cavity with tailor-made properties^1^,^2^. To assure a reliable assembly profile leading to the formation of the desired cavity and to lower the entropic cost of the process, covalent synthesis is often used to a large extent to spatially locate the basic constitutional elements and control the dimensions of the cavity. Impressive examples of self-assembling capsules based on both coordination and H-bonds have been reported with the cavity size ranging from almost negligible to giant (>10,000 Å^3^; ref. 3) and with the shape from almost ideally spherical to non-symmetrical, such as the ‘banana'- or ‘S'-shape^4^. At the same time another important and structurally related topology, H-bonded organic nanotubes, has recently been addressed^5^,^6^,^7^,^8^; however, with limited success in terms of the generality and simplicity of the strategy for the self-assembly process. As a rule, more structurally elaborated monomers give more predictable outcomes of the self-assembly, but unfortunately at the expense of synthetic efforts involved in their preparation. This is reasonable taking into account the entropic cost needed to assemble a large number of small monomers and the increase of the number of alternative aggregation pathways when several less preorganized monomers are used. All these challenges make it of highest interest to develop small and thus easily available monomers that self-assemble into open-end cavity aggregates capable of responding to external stimuli and switching between well-defined open, closed or polymeric forms, all possessing a tubular structure. The successful design of such responsive monomers could lead to switchable self-assembling transmembrane channels for drug delivery, shape-selective catalysts or insulator materials for molecular wires, among many other conceivable possibilities.

To accomplish this task, H-bonds are preferred over coordination bonds due to the more expressed dynamic nature of the former, especially when different conformers and tautomeric forms of the H-bonding unit are engaged in the assembly process. Moreover, for the initially formed cavity aggregate to be able to assemble further to a tube, additional H-bonding sites must be available to connect them into a polymer by rim-to-rim self-assembly (hierarchical self-assembly).

The ureidopyrimidinone (UPy) unit, first introduced by Sijbesma and colleagues^9^,^10^, represents the most reliable and useful H-bonding motif, extensively used in areas of supramolecular chemistry as diverse as supramolecular polymers^11^, receptors^12^, capsules^13^, chromophore dyads^14^ and even biological applications^15^. In the vast majority of cases, the UPy unit adopts a quadruple self-complementary H-bonding tautomer, that is, an AADD (A=H-bond acceptor; D=H-bond donor) unit or the corresponding enolic tautomeric form, DADA (Fig. 1a). This preference, however, could be changed when the electron-rich pyrrole ring is directly fused with UPy (herein referred as PUPy)^16^. Owing to electronic factors, the most stable tautomer becomes the one having the 6[*1H*]-pyrimidinone form of the isocytosine unit and existing as single conformer, where the terminal urea proton is H-bonded to an isocytosine nitrogen atom (Fig. 1b). In this form, it cannot participate in self-complementary quadruple H-bonding, but instead it has several 2H-bonding sites positioned at different angles. Although ditopic hydrogen bonds are usually not very strong, they could be significantly strengthened by their incorporation into cyclic arrays. The high level of preorganization of the monomer for cyclic aggregation could even result in a tautomeric form or a conformer otherwise not observed in simpler congeners. Such aggregation-induced tautomer/conformer shift is expected to be extremely favourable in cyclic systems to fulfill the maximum occupancy of H-bonding sites in response to geometric constraints in the monomer. The use of enantiomerically pure building blocks could further secure the tubular aggregation mode over the open linear one^7^.

Herein we report that by using an enantiomerically pure bicyclic H-bonding monomer, it is possible to access a collection of tubular supramolecular aggregates via interconversion of conformers within the hydrogen-bonding PUPy unit. The previously unobserved conformer of PUPy is shown to be essential in realizing efficient recognition of a C_60_ guest. The system described herein represents a unique case of a sophisticated stimulus-responsive, dynamic, one-component supramolecular system.

## Results

### Monomer design

**Figure 1:**
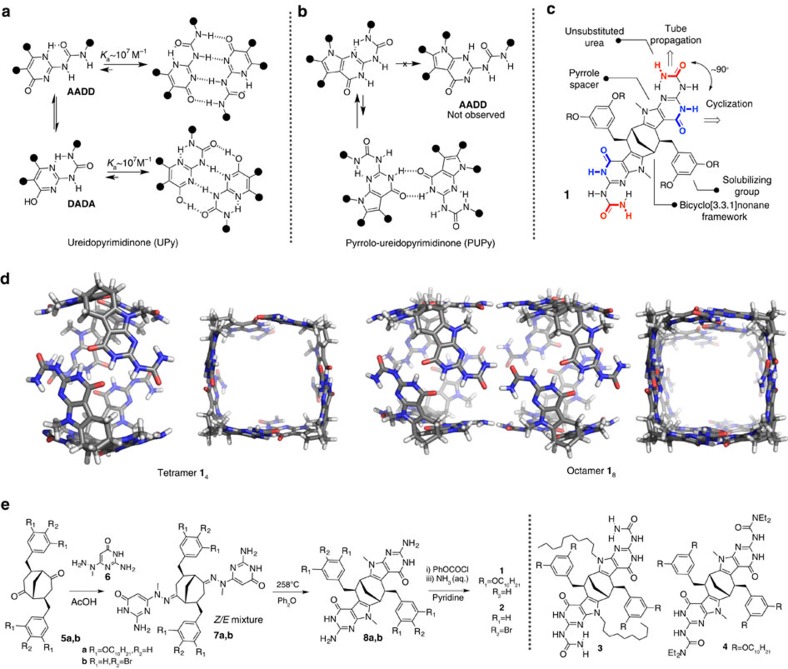
Monomer design and synthesis. (**a**) Two self-complementary AADD and DADA UPy tautomers and their exceptionally strong dimerization. (**b**) The most stable tautomer of PUPy derivatives and their weak dimerization via 2H-bonding. (**c**) Schematic representation of the essential design elements of supramolecular monomer **1**, allowing two orthogonal 2H-bonding directions (marked as red and blue). (**d**) Molecular models of 2H-bonded tetramer **1**_4_ and the corresponding tubular polymer assembly, depicted as octamer **1**_8_. The solubilizing chains were omitted for clarity. (**e**) Synthesis of monomers **1** and **2** having different solubilizing groups and control compounds **3** and **4**.

The design and synthesis of the building blocks, monomers **1** and **2**, and the two control compounds **3** and **4** are outlined in Fig. 1c–e. Each of the monomer comprises the enantiomerically pure bicyclo[3.3.1]nonane (BCN) framework^17^,^18^,^19^,^20^ to which two PUPys, bearing unsubstituted ureas, are attached via fused pyrrole rings. As depicted in Fig. 1c, the use of unsubstituted urea groups is essential to our design, as it introduces a second 2H-bonding site positioned orthogonally to the already existing 2H-bonding site in the isocytosine ring. Although the latter H-bonding site was envisioned to be utilized for the connection of the bicyclic V-shaped monomers into cyclic tetrameric assemblies, the former unsubstituted urea will be required for linking the resulting cyclic tetramers into a 2H-bonded polymeric tube (Fig. 1d). The present design of the monomers aimed at reaching tubular aggregation is built on the results from our previous studies, where we have demonstrated that all attempts to connect cyclic tetrameric units via H-bonds at the rim of the tetramer that are located too close to the bicyclic core were not successful. This is most likely to be due to the steric crowding arising from the bulky solubilizing groups situated on the core^20^. On the other hand, these groups were necessary to ensure solubility of the monomer. We reasoned that these complications could be overcome by placing the H-bonding sites required for tubular polymerization further away from the solubilizing groups using urea functional group as in monomer **1**. In addition, to secure a cyclic aggregation mode of high fidelity, we used an enantiomerically pure monomer. Monomer **1** was synthesized starting from the easily affordable enantiopure ketone **5** having solubilizing groups attached to the bicyclic framework (Fig. 1e)^21^. The straightforward thermal Fischer indolization of hydrazone **7** delivered intermediate **8**, which was then converted to the target compound **1** by the activation of the amino group in the isocytosine ring with phenylchloroformate and the subsequent treatment with ammonia. Monomer **2**, having the smaller *p*-bromobenzyl substituents attached to the BCN framework, was synthesized following the same reaction sequence as above and was needed as a more rigid analogue of **1** for atomic force microscopy (AFM) studies (*vide infra*). The control compound **3**, containing a larger pyrrole nitrogen substituent, and compound **4**, containing fully substituted terminal urea nitrogen atom, were obtained by modified procedures (Supplementary Fig. 1).

### Self-assembly and host–guest studies of monomer **1**

**Figure 2:**
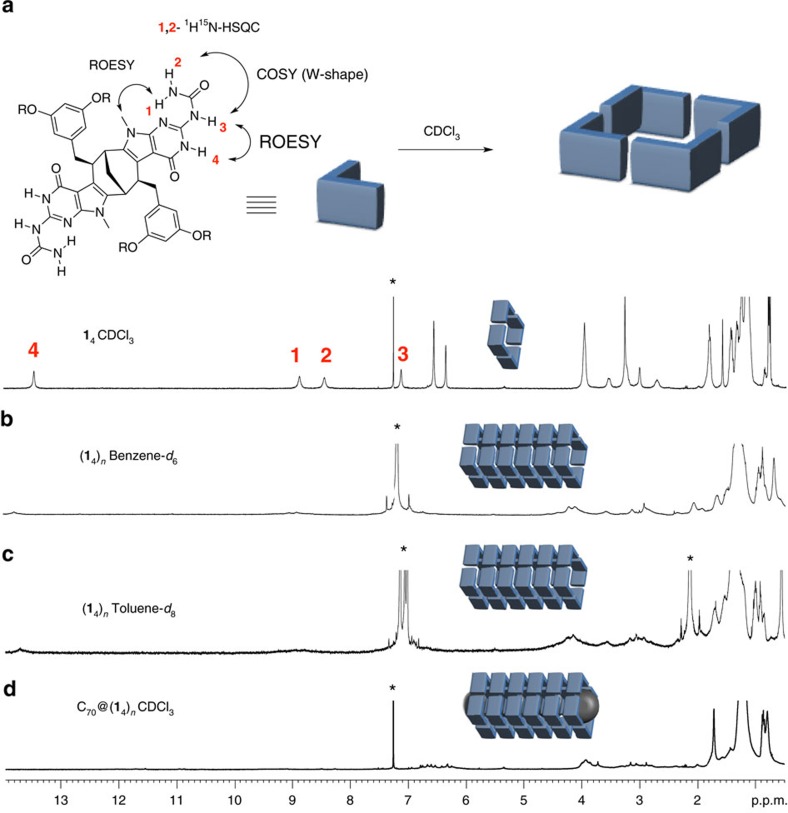
^1^H NMR spectra of monomer 1 in different solvents. (**a**) ^1^H NMR characterization of monomer **1** in CDCl_3_. (**b**) ^1^H NMR spectrum of **1** in benzene-*d*_6_. (**c**) ^1^H NMR spectrum of **1** in toluene-*d*_8_. (**d**) ^1^H NMR spectrum of **1** in CDCl_3_ in the presence of C_70_. *Indicates the residual solvent signal.

Monomer **1** was soluble in a variety of solvents of vastly different polarities, which allowed for the investigation of its aggregation properties in detail. When dissolved in CDCl_3_, **1** displayed a well-defined ^1^H nuclear magnetic resonance (NMR) spectrum, suggestive of both the formation of the anticipated tautomeric form of the PUPy units and the formation of one single type of cyclic aggregate through H-bonding (Fig. 2a). Namely, there are four NH protons corresponding to a *C*_2_-symmetric structure and also indicating restricted rotation of the urea unit due to an intramolecular H-bond (*δ* 8.88 p.p.m.) between the pyrimidine *sp*^2^ N atom and the urea proton 1. The chemical shift of proton 4 (*δ* 13.43 p.p.m.) supports its involvement in intermolecular H-bonding within a cyclic tetramer as originally designed. On the other hand, the chemical shift of the proton 3 (*δ* 7.12 p.p.m.) indicates that this proton is not a part of the H-bonding array (Figs 1c,d and 2a). The unambiguous assignment of all NH protons was made using several NMR techniques (Supplementary Figs 25–34). Hence, protons 1 and 2 were shown to reside on the same nitrogen atom as evidenced by ^1^H–^15^N heteronuclear single quantum coherence spectroscopy and site-selective labelling with the ^15^N isotope (Supplementary Fig. 32). The strong long-range coupling between protons 2 and 3, separated by four single bonds, was observed in the ^1^H–^1^H correlated (correlation spectroscopy) spectrum and is easily explained as a W-coupling, fully consistent with the geometry of the proposed conformer (Fig. 1c). Moreover, the observed rotating-frame nuclear Overhauser effect correlations between isocytosine protons 3 and 4, as well as between urea proton 1 and the protons of *N*-methyl group of the pyrrole moiety further corroborated the AD-DA arrangement of the H-bonding sites positioned in the right direction for envisioned lateral and longitudinal aggregation of monomer.

To confirm that monomer **1** exists as a H-bonded tetramer in CDCl_3_, we performed diffusion-ordered spectroscopy experiments, which showed a correlation of all the ^1^H resonances to a single diffusion coefficient, which translated to a hydrodynamic radius, *R*_H_=10 Å, consistent with an aggregate significantly larger than the monomer and in good agreement with the values previously obtained for related tetrameric assemblies (Supplementary Fig. 35)^12^,^18^. In addition, the high stability of the aggregate in CDCl_3_ was evidenced from the insensitivity of the NH proton resonances to dilution or heating, as well as the observation of negligible tailing of the corresponding peak in the gel permeation chromatogram (GPC) (Supplementary Figs 36, 37 and 48). The latter confirmed the presence of a single aggregate with polydispersity index=1.02.

**Figure 3:**
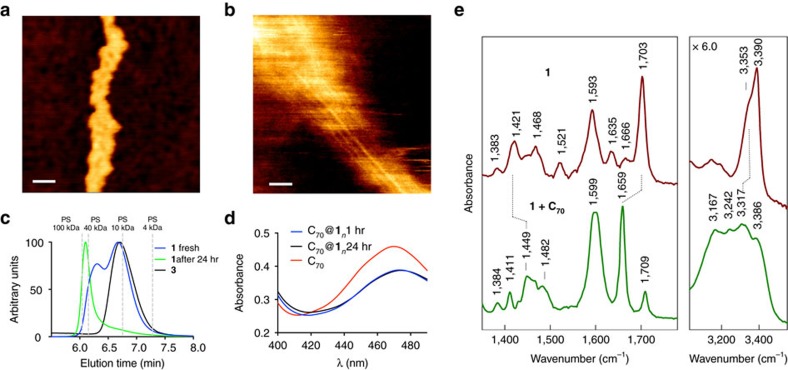
Characterization of supramolecular tubular polymers. (**a**) AFM image of **1** deposited on silicon surface. (**b**) AFM image of **2** deposited on mica surface. (**c**) Normalized GPC trace of the solution of **1** (fresh and aged) and control compound **3** in toluene. The vertical dashed lines correspond to the retention times of polystyrene (PS) standards. (**d**) Ultraviolet–visible spectra of C_70_ and C_70_/**1** 1:4 mixture in CDCl_3_. (**e**) Fourier transform infrared spectra of **1** (top) and C_70_/**1** 1:4 mixture (bottom) in CDCl_3_. Scale bars, 100 nm (**a**) and 100 nm (**b**).

Having shown that compound **1** exists only as a tetramer in CDCl_3_, we further reasoned that the anticipated polymer was not formed due to the much weaker H-bonds between terminal urea units as compared with strong intra-cyclic H-bonds. We therefore decreased the polarity of the solvent switching from chlorinated to aromatic solvents, such as toluene and benzene, to increase the strength of the H-bonds. Indeed, the formation of high-molecular-weight aggregates was first detected by the inspection of the ^1^H NMR spectra, where extreme broadening of the signals and complete disappearance of the NH resonances were observed, typical for polymeric materials (Fig. 2b,c). Most remarkably, the formation of polymeric aggregates could be triggered even in the more polar CDCl_3_, when a C_70_ guest was added (Fig. 2d)^22^. In both cases, the formation of polymeric aggregates was confirmed by AFM (Fig. 3a,b), dynamic light scattering and GPC analysis (Fig. 3c). According to the dynamic light scattering data, oligomeric assemblies with *R*_H_=11 nm and *R*_H_=20 nm were formed in toluene and C_70_/CDCl_3_ solution, respectively, corresponding roughly to 15–20 cyclic tertameric units in the chain (Supplementary Fig. 41). GPC experiments with monomer **1** and control compound **3** in toluene indicated the formation of supramolecular polymers and a discrete cyclic tetramer, respectively. The fresh solution of **1** displayed a bimodal distribution of molecular mass, probably corresponding to a mixture of oligomers and cyclic tetramers (Fig. 3c, blue line). The lower-molecular-weight peak was found to have almost the same retention time as compound **3**, which can only form tetrameric assemblies **3**_4_, due to the steric hindrance between the decyl groups on the pyrrole nitrogen atom of **3**_4_ units within polymer (**3**_4_)_*n*_. During ageing, however, the signal of the cyclic tetramer **1**_4_ disappears completely, leading to the formation of high-molecular-weight supramolecular polymers (Fig. 3c, green line). The characterization of the formed supramolecular tubular polymers casted on surface (silicon and mica) from a toluene or a C_70_/CDCl_3_ solution by AFM revealed fibre-like superstructures where multiple molecular tubes were stacked together side-by-side (Fig. 3a). Decreasing the concentration did not prevent this unwanted aggregation of the tubular aggregates. Therefore, a more rigid analogue of monomer **1**, that is, compound **2** (Fig. 1e) was used instead. Indeed, although the lateral interaction of the tubes was not eliminated completely, it was decreased substantially and long rigid tubular aggregates were clearly observed in this case (Fig. 3b).

To probe the involvement of the terminal urea moiety in the H-bonding, Fourier transform infrared spectroscopy was utilized for the C_70_/**1** mixture in CDCl_3_ (Fig. 3e). The vibrational bands for the terminal N–H and carbonyl groups of the urea moiety were unambiguously assigned using an ^15^N-labelled derivative of **1** and model compound **8a**, which does not possess the urea group (Supplementary Figs 49 and 50). As seen from Fig. 3e, the addition of C_70_ to a CDCl_3_ solution of **1** triggered a remarkable change in the spectrum, namely, the *ν* (C=O) band of the urea group in **1** at 1,703 cm^−1^ downshifted by 44 cm^−1^, whereas the *ν*_a_ (C–N) band at 1,421 cm^−1^ upshifted by 28 cm^−1^. Moreover, the *ν* (N–H) band downshifted from 3,353 to 3,317 cm^−1^. All these changes are consistent with the involvement of both the C=O and NH_2_ groups of the urea moiety of **1** in the H-bonding interactions.

The two control compounds **3** and **4** were tested under identical polymerization conditions as for **1**. In compound **3**, long decyl chains attached to the nitrogen atom of the pyrrole ring should prevent the polymerization of cyclic units by exerting steric hindrance, whereas in compound **4** the terminal NH_2_ group of the urea moiety is fully alkylated and is thus incapable of H-bonding. As expected, both control compounds failed to provide supramolecular polymers in the broad range of tested solvents with or without C_70_ guest added.

Concerning the role of C_70_ to trigger the polymerization of **1**, the exact mechanism remains unknown. However, ultraviolet–visible studies showed that the C_70_ molecule is incorporated into the cavity of the tube as evident from the hypochromic effect and the bathochromic shift of the absorption band of C_70_ at 470 nm by 6 nm (Fig. 3d). In addition, ^1^H NMR titration experiments showed that the tubular polymers are saturated with C_70_ at a molar fraction of 0.25, corresponding to one C_70_ molecule per cyclic tetramer. It is likely to be that the C_70_ guests experience attractive forces inside the tube and brings the cyclic tetrameric units together. In fact, the bathochromic shift observed is consistent with a C_70_–C_70_ interaction^23^,^24^ and is similar to the known attractive interaction of C_60_ molecules in a confined environment^25^. The polymeric peapod inclusion complexes with C_70_ so obtained could be attractive for their possible application in the field of organic photovoltaics.

**Figure 4:**
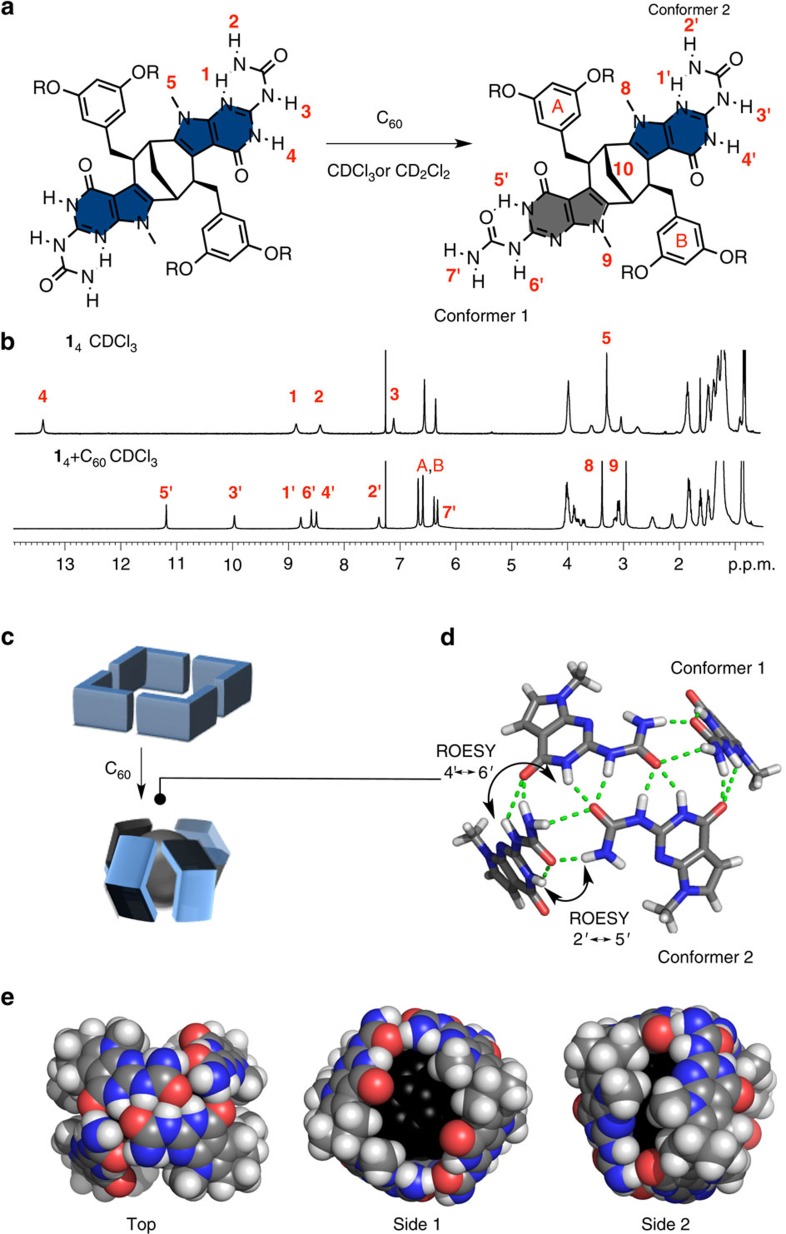
Structure of the insertion complex C_60_@1_4_. (**a**) Symmetry breaking of monomer **1** by conformer redistribution in PUPy units. Conformer 1 (grey) and conformer 2 have the same 6[*1H*]-pyrimidinone tautomeric form but different intramolecular H-bonds. (**b**) ^1^H NMR spectrum of **1**_4_ (top) and C_60_@**1**_4_ (bottom). (**c**) Schematic representation of starting tetramer **1**_4_ and capsular complex C_60_@**1**_4_. (**d**) Top view of H-bonding interface between two conformers of PUPy with rotating-frame nuclear Overhauser effect interactions indicated. H-bonds are presented by dashed green lines. (**e**) Top and side views of the optimized molecular models of C_60_@**1**_4_. Carbons in C_60_ molecule are labelled black.

Intrigued by the role of C_70_ in inducing polymerization of monomer **1** in chlorinated solvents, we also probed the effect of C_60_ guest on the aggregation profile of **1** in chloroform. Surprisingly, based on the NMR data, only one well-defined inclusion complex was obtained as opposed to polymeric aggregates with C_70_. Compared with **1**_4_ in CDCl_3_, each resonance of the NH protons was split into two upon the addition of C_60_ (Fig. 4a,b). A single carbon resonance for the C10 atom provided compelling evidence that symmetry breaking had taken place, resulting in an aggregate composed of one C_1_-symmetric monomer and not a 1:1 mixture of two *C*_2_-symmetric monomers (Fig. 4a). Careful analysis of the ^1^H NMR data revealed two conformers of the PUPy unit, both containing the 6[*1H*]-pyrimidinone tautomer of the isocytosine ring. Namely, conformer 1 having an intramolecular H-bond between an N–H proton in isocytosine and an urea C=O group (Fig. 4a, conformer 1) and conformer 2 having an intramolecular H-bond between a urea N–H donor and an isocytosine *sp*^2^ N acceptor, as in the original tetramer **1**_4_ (Fig. 4a, conformer 2). Conformer 1 has never been observed before for PUPy derivatives, whereas in case of UPy derivatives, it is considered detrimental for the dimerization due to its non-self-complementary nature. The cross-peaks between protons 4′ and 6′, and protons 2′ and 5′ in the rotating-frame nuclear Overhauser effect spectrum indicated that these two conformers are located in close proximity to each other and are therefore indicative of an alternating arrangement of these units within the aggregate. Unfortunately, the resonances assigned to terminal protons in the urea moiety, that is, protons 7′ in conformer 1 were too broad to be clearly visible and overlapped with the aromatic ring protons A and B (Fig. 4b). The latter observation and the value of the chemical shift of protons 7′ (*δ* 6.50–7.00 p.p.m.) suggested that they are loosely bound in the aggregate and are exchanging through a rotation of the CO–NH_2_ bond. Inspection of molecular models showed that the only possible arrangement of the two conformers that would satisfy the above requirement is in a capsule-like structure, where monomers are arranged side-to-side rather than end-to-end, as in the original tetramer **1**_4_ (Fig. 4c). In this way, two terminal urea groups in conformer 2 are 2H-bonded with an additional stabilization by a secondary H-bond from the isocytosine N–H donor located in the proximity. To this dimer, two conformers 1 are attached at both ends via H-bond between the urea proton 6′ and the carbonyl group of the isocytosine ring and between the carbonyl group of the urea moeity and proton 2′ in the other conformer (Fig. 4d). The protons 7′ are only weakly bound to the carbonyl groups of isocytosine and urea moieties of conformer 2 and as a result of geometric constrains they are slightly bended out from the plane of the H-bonding interface, in agreement with the broadening of the corresponding NH resonances observed in ^1^H NMR (Fig. 4b). In addition, the large upfield shift of the isocytosine NH proton 4′ of conformer 2 in the complex C_60_@**1**_4_ is in accord with its much weaker out-of-plane intermolecular H-bonding with the carbonyl group of the urea moiety as opposed to nearly optimal H-bonding in the free tetramer **1**_4_ (Fig. 4b,d). The 4:1 (**1**:C_60_) stoichiometry of the complex was determined from molar-ratio method using ^1^H NMR (Supplementary Fig. 61) and is also in line with an even number of monomers required for their symmetric alternating arrangement within the aggregate. Moreover, the control compound **3** with bulkier decyl groups on the pyrrole ring was not able to form the corresponding capsule-like insertion complex due to very unfavourable intermolecular steric interactions between decyl chains (Fig. 4e, Side 2). The other control compound **4** did not interact with C_60_ either, because the corresponding capsule cannot form without H-bond donors on the terminal urea group (Fig. 4d). According to molecular modelling (Supplementary Fig. 62), the capsule embraces the C_60_ guest (*d*=0.71 nm) so tightly that having the same diameter but being only slightly longer C_70_ (*d*_1_=0.712 nm, *d*_2_=0.796 nm) is already too large to fit into the cavity and thus the formation of this type of capsule is no longer possible. For this reason, an inclusion complex with C_70_ was first formed with an open form of the **1**_4_ tetramer, which ultimately led to polymers (C_70_@**1**_4_)_*n*_ via rim-to-rim aggregation of C_70_@**1**_4_ (Fig. 2d). The affinity of the host **1** for C_60_ and C_70_ was found to be very similar based on the liquid–solid extraction studies (Supplementary Table 2). The system described above represents a responsive and sensitive supramolecular system for the fine-tuning between different open and closed tubular topologies. The distinctive spatial arrangement of the H-bonding sites and the possibility for the interconversion of conformers are the hallmarks of this system.

### Systems chemistry of tubular aggregates

**Figure 5:**
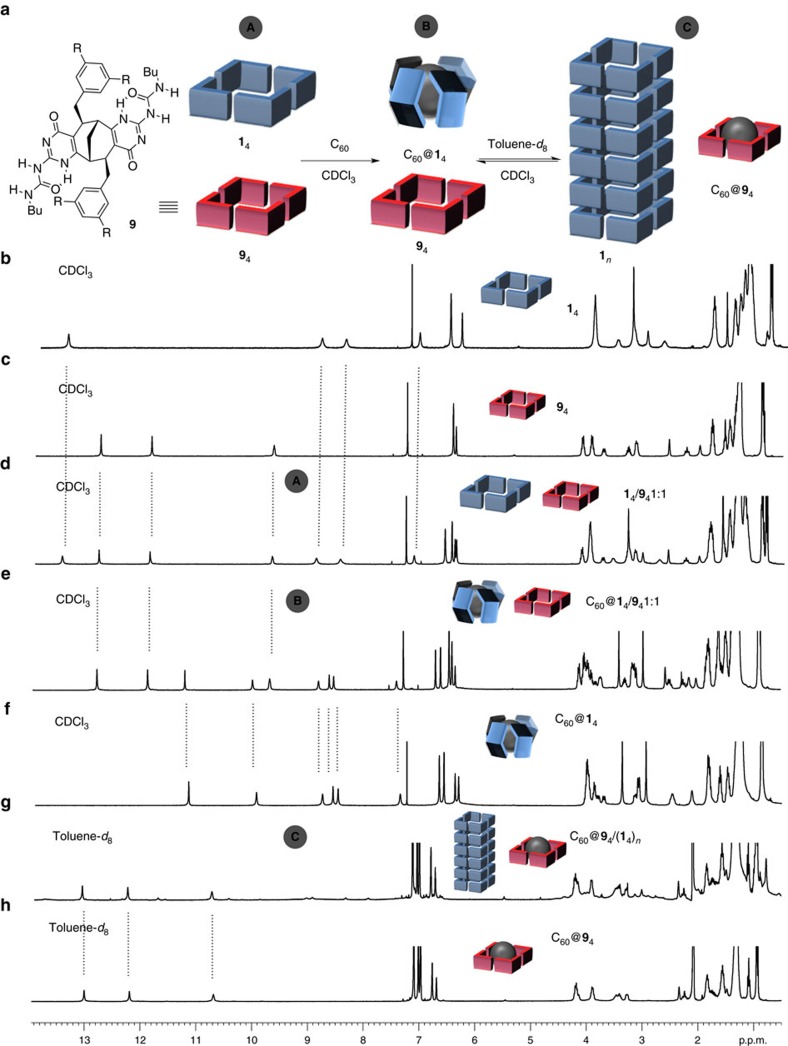
Self-sorting studies and ^1^H NMR spectra of three-component supramolecular system. (**a**) Chemical structure of 4H-bonding monomer **9** and schematic representation of self-sorting (process A), selective encapsulation of C_60_ guest (process B) and capsule-tube isomerization (process C). (**b**) ^1^H NMR spectrum of **1**_4_ in CDCl_3_. (**c**) ^1^H NMR spectrum of **9**_4_ in CDCl_3_. (**d**) ^1^H NMR spectrum of 1:1 mixture of **1**_4_ and **9**_4_ in CDCl_3_ (process A). (**e**) ^1^H NMR spectrum of C_60_@**1**_4_ and **9**_4_ in CDCl_3_ after addition of 0.25 eq. of C_60_ (process B). (**f**) ^1^H NMR spectrum of C_60_@**1**_4_ in CDCl_3_. (**g**) ^1^H NMR spectrum of (**1**_4_)_*n*_ and C_60_@**9**_4_ in toluene-*d*_8_ (process C). (**h**) ^1^H NMR spectrum of C_60_@**9**_4_ in toluene-*d*_8_.

The facile switch between polymeric supramolecular tubes and well-defined capsules by guest or solvent stimuli encouraged us to devise an even more complex multicomponent supramolecular system with an external modulator of the guest^26^,^27^. For this purpose, the known *C*_2_-symmetric bicyclic monomer **9** (Fig. 5), composed of the BCN scaffold and two UPy derivatives fused to its termini was used^12^. From our previous studies, it is well-established that monomer **9** forms a cyclic tetrameric aggregate **9**_4_, which does not interact with a C_60_ guest in CDCl_3_ (ref. 12). However, after changing the solvent to toluene, **9**_4_ becomes a very efficient host for fullerenes and thus it might act as a C_60_ scavenger. Cycling between the solvents chloroform and toluene for the **9**_4_/C_60_ mixture would then represent a convenient switching system for C_60_ catch-release (ON/OFF) events provided that aggregate **9**_4_ is narcissistically self-sorted^28^,^29^ in the presence of monomer **1** and has higher affinity for C_60_ compared with tubular polymers (**1**_4_)_n_.

To implement the above design elements into a functional system, we then first probed the self-sorting properties of monomer **1** and **9** in CDCl_3_ solution. As seen from Fig. 5d (process A), the ^1^H NMR experiment revealed that no exchange of monomers between two tetrameric assemblies **1**_4_ and **9**_4_ (1:1 mixture) took place, indicating high fidelity of the UPy and PUPy units for homoaggregation. The addition of 0.25 eq. of C_60_, resulted in the selective formation of a capsular inclusion complex C_60_@**1**_4_, leaving **9**_4_ intact (Fig. 5e, process B). The identity of all species was confirmed by comparing the corresponding ^1^H NMR spectrum of the mixture to the ones of separately prepared samples (Fig. 5c,e). When CDCl_3_ was replaced for toluene-*d*_8_, the inclusion capsule C_60_@**1**_4_ rearranged into the tubular polymer of **1**_4_, whereas concomitantly the C_60_ guest moved into the cavity of **9**_4_ (Fig. 5g, process C). The emergence of characteristic resonances attributed to C_60_@**9**_4_ were clearly observed in the ^1^H NMR spectrum, whereas the formation of (**1**_4_)_*n*_ was evidenced by the broadening and the eventual disappearance of all NH resonances corresponding to C_60_@**1**_4_ (Fig. 5g,e). The higher stability of complex C_60_@**9**_4_ in comparison with possible peapod complexes (C_60_)_*m*_(**1**_4_)_*n*_, is a direct effect of a perfect match of sizes between C_60_ and the cavity of **9**_4_. The whole process is fully reversible and after switching the solvent from toluene-*d*_8_ back to CDCl_3_, the initial state, C_60_@**1**_4_/**9**_4_, was recovered. The swapping of the C_60_ guest in the present supramolecular system guarantees that only one host is empty (disregarding the solvent molecules) at the time and may find applications as a selective switch in a future generation of more complex systems where a larger number of guests are present.

## Discussion

In conclusion, this study provides an example of a sophisticated dynamic tubular H-bonded system generated from one single type of small, highly preorganized monomer (**1**). The monomer easily changes its aggregation mode in response to the solvent or guest in a unique manner, resulting in an open-ended cyclic structure, a closed shell capsule or polymeric tubular assemblies. For the first time, molecular tubes were assembled between small organic molecules, where both the cyclic framework and the polymerization were accomplished by H-bonding. This was achieved using a monomer containing a non-self-complementary form of UPy derivative fused with an electron-rich pyrrole ring. Besides the control of the polymerization by the nature of the solvent, also the formation of tubular polymeric inclusion complex was demonstrated by the addition of C_70_ guest molecules. Owing to the ordered arrangement of electron conductive C_70_ molecules within the tube, these assemblies can be considered for their potential implementation into organic photovoltaic devices. In addition, the highly dynamic nature of monomer **1** was revealed in the formation of a capsule-like complex with a C_60_ guest, where two different conformers embedded within a single monomer were interacting. These modes for the self-assembly of structurally simple monomers open new possibilities to easily create stimuli-responsive, complex multicomponent systems of different topologies.

## Methods

### Materials and characterization

All chemicals were used as received from commercial suppliers. All moisture-sensitive reactions were carried out under an atmosphere of dry nitrogen using oven-dried glassware. ^1^H NMR (400 MHz) and ^13^C NMR (100 MHz) were recorded on a Bruker Ascend spectrometer. Chemical shifts are given in parts per million relative to the residual solvent signals at *δ*=7.27 (^1^H NMR) and 77.16 (^13^C NMR) p.p.m. in CDCl_3_ and *δ*=7.00 (^1^H NMR) and 128.33 (^13^C NMR) p.p.m. in toluene-*d*_8_. Enantiomeric excess was determined with a Perkin-Elmer Autosystem XL Gas Chromatograph using Alpha DEXTM 120 fused silica capillary column. Elemental analysis was performed at A. Kolbe Mikroanalytisches Laboratorium, Germany, and Microanalysis Laboratory in Department of Organic Chemistry, Vilnius University.

### Synthesis of **7a**

A suspension of compound **5a** (3.17 g, 3.31 mmol, 1.0 eq.) and 2-amino-6-(1-methylhydrazinyl)-4(3H)-pyrimidinone **6** (1.32 g, 8.51 mmol, 2.6 eq.) in acetic acid (185 ml) was stirred at room temperature. After 19 h, the solvent was removed under reduced pressure at 30 ^°^C. Toluene was used to remove residues of acetic acid. Then, it was dried under high vacuum overnight and subjected to flash chromatography. Elution with gradient eluent system (CH_2_Cl_2_/MeOH+1% Et_3_N, 100/1, 60/1, 40/1, 30/1, 20/1) afforded 2.81 g (69% yield) of compound **7a**. Compound **7a** was obtained as a mixture of *E* and *Z* isomers, which gave a very complicated ^1^H NMR spectrum. For this reason, full characterization of **7a** was not attempted and the compound was used directly in the next step.

HRMS (ESI) calcd. for ([M+H]^+^): C_73_H_119_N_10_O_6_ 1231.9314; Found: 1231.9343.

### Synthesis of **8a**

A solution of compound **7a** (2.50 g, 2.0 mmol) in diphenyl ether (95 ml) was heated to reflux with an air condenser passing nitrogen stream through the mixture via a long cannula. After 29 h, the evolution of ammonia ceased and the reaction mixture was cooled to room temperature. The reaction mixture was directly loaded on the silica gel column and diphenyl ether was removed by using petroleum ether as eluent. Then, the eluent was changed to CH_2_Cl_2_/MeOH (10/1), to afford 2.34 g of partially purified product. The crude product was further purified by flash chromatography with gradient eluent system (CH_2_Cl_2_, then CH_2_Cl_2_/MeOH 100/1, 80/1, 50/1, 30/1, 20/1) to afford 1.65 g (68% yield) of compound **8a** as a yellowish glass. ^1^H NMR (400 MHz, CDCl_3_) *δ* 12.90 (br. s, 1H), 6.60 (d, *J*=4.0 Hz, 2H), 6.34 (s, 1H), 4.71 (s, 2H), 3.93 (t, *J*=8.0 Hz, 4H), 3.52 (d, *J*=8.0 Hz, 1H), 3.02–3.08 (m, 2H), 2.98 (s, 3H), 2.42 (t, *J*=12 Hz, 1H), 2.08 (s, 1H), 1.72–1.79 (m, 4H), 1.40–1.44 (m, 4H), 1.26–1.31 (m, 24H), 0.87 (t, *J*=8.0 Hz, 6H); ^13^C NMR (100 MHz, CDCl_3_) *δ* 161.55, 160.39, 151.47, 151.12, 144.45, 131.5, 113.04, 108.40, 98.72, 98.57, 68.16, 41.59, 41.59, 32.03, 29.73, 29.71, 29.58, 29.54, 29.46, 27.66, 27.33, 26.24, 22.82, 21.37, 14.26; IR *ν*_max_/cm^−1^ (in CDCl_3_) 3,507, 3,406, 1,664, 1,634, 1,596, 1,460; HRMS (ESI) calcd. for ([M+H]^+^):C_73_H_113_N_8_O_6_ 1197.8783; Found: 1197.8761; Anal. calcd. for C_73_H_112_N_8_O_6_·1/5 CH_2_Cl_2_: C, 72.38; H, 9.33; N, 9.22; Found: C, 72.54; H, 9.34; N, 8.83.

### Synthesis of **1**

Compound **8a** (0.53 g, 0.44 mmol, 1.0 eq.) was dissolved in anhydrous pyridine (30 ml) under N_2_ and the solution obtained was cooled in an ice bath. Then PhOCOCl (0.61 ml, 4.86 mmol, 11.0 eq.) was added dropwise. After 10 min, the ice bath was removed and the reaction mixture was stirred at room temperature for 22 h. Then it was cooled to 0 ^°^C again and 25% NH_3_ (aq.) (2.8 ml) was added dropwise. The resulting mixture was allowed to reach room temperature and stirred overnight. The suspension was quenched with excess of 10% HCl and extracted with CH_2_Cl_2_. The combined organic phase was washed with sat. NaHCO_3_, dried with Na_2_SO_4_ and evaporated. The residue was triturated with MeOH (300 ml) and sonicated for several minutes. The suspension was filtered to give 0.43 g (77% yield) of compound **1** as an off-white solid. ^1^H NMR (400 MHz, CDCl_3_) *δ* 13.43 (s, 1H), 8.88 (s, 1H), 8.44 (s, 1H), 7.12 (s, 1H), 6.56 (s, 2H), 6.36 (s, 1H), 3.97 (br. s, 4H), 3.55 (d, *J*=8.0 Hz, 1H), 3.28 (m, 4H), 3.02 (s, 1H), 2.72 (br. s, 1H), 1.81–1.84 (m, 5H), 1.15–1.45 (m, 28H), 0.80 (t, *J*=8.0 Hz, 6H); ^13^C NMR (100 MHz, CDCl_3_) *δ* 160.29, 160.01, 156.72, 147.80, 145.20, 143.46, 132.67, 114.01, 108.15, 101.35, 99.18, 68.09, 41.44, 40.18, 31.99, 29.87, 29.75, 29.70, 29.53, 29.43, 28.30, 28.30, 26.24, 22.77, 20.99, 14.18; IR ν_max_/cm^−1^ (in CDCl_3_) 3,390, 3,353, 1,703, 1,593, 1,468, 1,421; HRMS (ESI) calcd. for ([M+H]^+^): C_75_H_115_N_10_O_8_ 1283.8899; Found: 1283.8911; Anal. calcd. for C_75_H_114_N_10_O_8_: C, 70.17; H, 8.95; N, 10.91; Found: C, 70.01; H, 8.92; N, 10.86.

For further information on Methods, see the Supplementary Information.

### Data availability

The authors declare that the data supporting the findings of this study are available within the paper and its Supplementary Information file.

## Additional information

**How to cite this article:** Shi, Q. *et al*. Stimuli-controlled self-assembly of diverse tubular aggregates from one single small monomer. *Nat. Commun.* **8,** 14943 doi: 10.1038/ncomms14943 (2017).

**Publisher's note**: Springer Nature remains neutral with regard to jurisdictional claims in published maps and institutional affiliations.

## Supplementary Material

Supplementary Information Supplementary Figures, Supplementary Tables, Supplementary Methods and Supplementary References.
