# Supplementary material for: Stimuli-controlled self-assembly of diverse tubular aggregates from one single small monomer
Source: Nat Commun. 2017 Apr 12;8:14943. doi: 10.1038/ncomms14943 (PMC5394284; doi:10.1038/ncomms14943)
Supplement: Supplementary Information — Supplementary Figures, Supplementary Tables, Supplementary Methods and Supplementary References. [file ncomms14943-s1.pdf]

Supplementary Methods

General information. All chemicals were used as received from commercial suppliers. All moisture sensitive reactions were carried out under an atmosphere of dry nitrogen using oven-dried glassware. Flash column chromatography was performed on Matrex (25-70µm). TLC was done on aluminum sheets precoated with silica gel 60 F_{254} (Merck). The TLC plates were visualized with UV light (general), aq. $KMnO_4$ solution (for unsaturated compounds) and vanilin solution (general). Melting points were determined in Electrothermal IA9000 SERIES Digital Melting Point Aparatus and were uncorrected. ^{1}H and ^{13}C spectra were recorded either on a Bruker 400 MHz or 500 MHz spectrometers. Chemicals shifts are given in parts per million relative to TMS using the residual solvent peaks at δ= 7.27 (^{1}H NMR) and 77.16 (^{13}C NMR) ppm in $CDCl_3$ and δ= 7.00 (^{1}H NMR) and 128.33 (^{13}C NMR) ppm in toluene-d_8. Enantiomeric excess was determined with a Perkin-Elmer Autosystem XL Gas Chromatograph using Alpha DEXTM 120 fused silica capillary column (30 m×0.25 mm×0.25µm film thickness). Elemental analyses were performed at A. Kolbe Mikroanalytisches Laboratorium, Germany and Microanalysis Laboratory, Department of Organic Chemistry, Vilnius University.

Synthesis

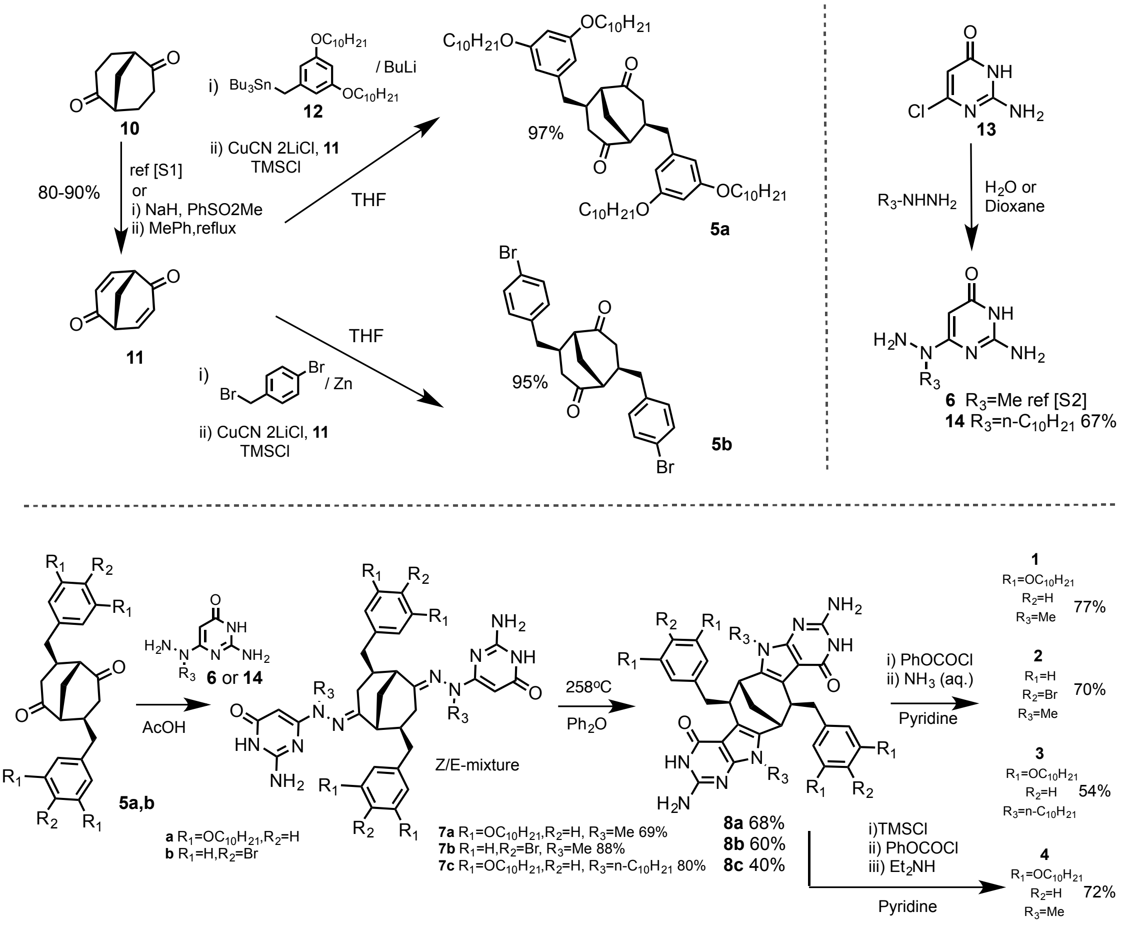

Supplemetary Figure 1. Synthesis of compounds 1-4. The synthesis of the target compounds **1-4** is based on the Fischer indolization reaction between corresponding bicyclic ketones and pyrimidine hydrazines . The starting enantiopure diketone (+)-(1R,5R)-**10** was obtained in multigram quantities by kinetic resolution of racemic **10** with bakers yeast [3]. Diketone **10** was converted into dienone **11** either via one-pot selenation-selenoxide elimination using Barton conditions[1] (milligram scale) or via two step procedure based on sulfoxide elimination (multigram scale). Dienone **11** was further used for the synthesis of diketones having the required solubilizing group. For compound **5** having bulky bis(decyloxy)benzyl groups tin-lithium exchange was used to obtain, first benzylic organolithium intermediate, which was then transmetalated to corresponding cuprate using soluble CuCN·2LiCl salt. Direct synthesis of benzyllithium or benzylmagnesium derivative was not possible due to extensive Wurtz coupling. On the other hand, p-bromobenzyl derivative **5b** was obtained without problems using direct oxidative insertion of zinc followed by transmetalation with copper (I). Diketones 5a,b were treated with hydrazine derivatives 6 and 14 containing isocytosine moeity at rt in AcOH to provide corresponding bis-hydrazones, which were used directly in the next step. The acid catalysed low-temperature Fischer indolization is not efficient with heteroaromatic hydrazones, therefore thermal conditions were utilized [4]. The heating mantle was used to provide high temperature (>300°C) and argon stream was constantly passed through the reaction mixture throughout the course of the reaction (**Supplemetary Fig. 2**). The amino group of the pyrrolo-isocytosine derivatives 8a-c obtained were activated with phenyl chloroformate before the addition of ammonia (for compounds 1-3) or diethylamine (for compound 4). In the first version of this transformation, the temporal protection of OH-groups with TMSCl was used (see synthetic procedure for compound 4), however, later it was found that redundant phenyloxycarbonyl functionalities on isocytosine oxygen atoms were readily cleaved with an excess of ammonia or amine used for urea synthesis.

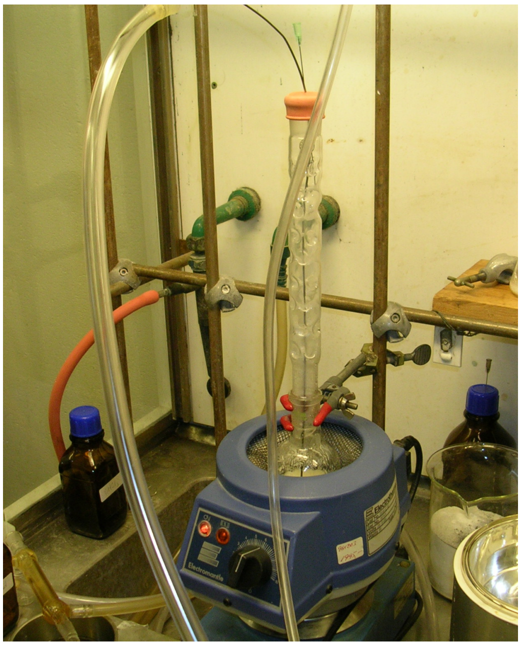

Supplementary Figure 2. Typical reaction setup for thermal Fischer indolization.

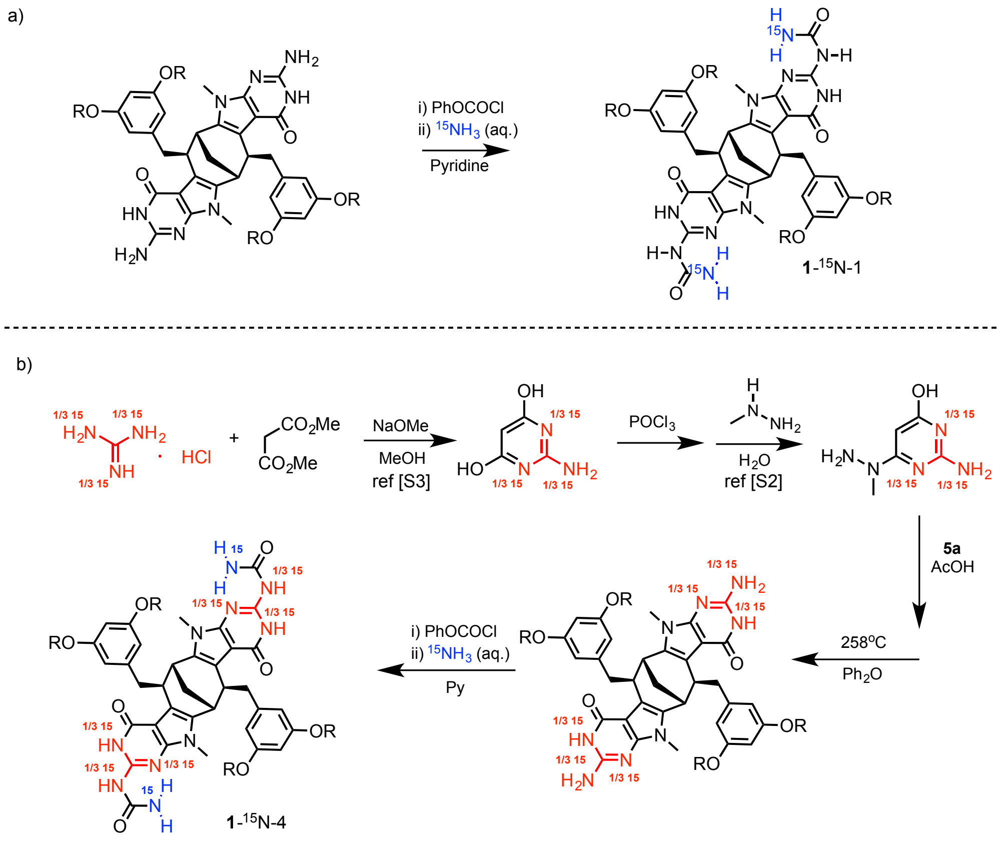

Supplementary Figure 3. Synthesis of ^{15}N labelled compounds 1-^{15}N-1 and 1-^{15}N-4. ^{15}N single-site (1-^{15}N-1) and multi-sites (1-^{15}N-4) labelled versions of 1 were synthesized either by adopting exactly the same synthetic procedures as for non-labelled compounds or using methods reported in literature.

Synthetic procedures

Compound 11

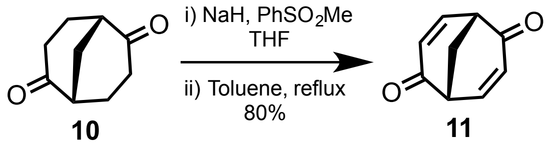

Under nitrogen, to a stirred suspension of sodium hydride (7.85 g, 0.20 mol, 60 % dispersion in mineral oil) in dry THF (30 mL) was added methyl benzenesulphinate (11.3 g, 9.50 mL, 77.0 mmol) at once. A solution of (+)-(1R,5R)-**10** (5.00 g, 32.9 mmol) in dry THF (40 mL) was added dropwise and the reaction mixture was stirred at rt overnight. The green-yellow suspension was quenched with 4% phosphoric acid until pH 3-4. After addition of water (50 mL), the aqueous phase was extracted with ethyl acetate. The combined organic fractions were washed with brine, dried with Na_2SO_4 and concentrated under reduced pressure yielding 18.1 g of a crude yellow oil, which was subjected to flash chromatography (ethyl acetate). Besides unreacted methylbenzene sulfinate (R_f 0.84), two other fractions, consisting of the compounds 3,7-bis(phenylsulfinyl)bicyclo[3.3.1]nonane-2,6-dione (R_f 0.28) and 7- (phenylsulfinyl)-bicyclo[3.3.1]non-3-ene-2,6-dione (R_f 0.45) were obtained. To the mixture of above compounds in toluene (80 mL), sodium carbonate (17.4 g, 0.16 mol) was added and the reaction mixture was brought to reflux. After 10 min., TLC (petrol ether/ethyl acetate-6:1) showed complete conversion and the reaction mixture was cooled to rt and filtered. The filter cake was washed with toluene (60 mL) and the green clear filtrate was evaporated. The residue was purified by flash chromatography (petrol ether/ ethyl acetate-6:1), yielding 3.90 g (80 %) of (+)-(1R,5R)-**11** as an yellowish solid, which was stored in the fridge under nitrogen, protected from ambient light.

Racemic **11** was obtained using the same procedure starting from racemic **10**.

m.p. 105°C (transition at 90°C);

^{1}**H NMR** (400 MHz, $CDCl_3$) δ 7.02 (dd, J = 10.0, 6.8 Hz, 2H), 5.91 (d, J = 10.0 Hz, 2H), 3.37, (dt, J = 6.8, 2.9 Hz, 2H), 2.79 (t, J = 2.9 Hz); ^{13}**C NMR** (100 MHz, $CDCl_3$) δ 192.4, 145.7, 125.6, 46.0, 34.8. **IR** ν_{max}/cm^{-1} 3040, 2941, 1653, 1559; **HRMS** (ESI) calcd. for [M+H]: $C_9H_8O_2$: 149.0603; Found: 149.0208; Anal. calcd for $C_9H_8O_2$: C 72.94, H 5.45; Found: C 73.18, H 5.43.

Compound 5a

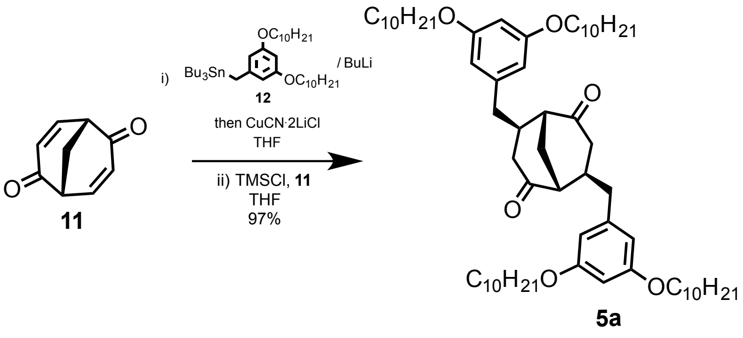

A solution of (3,5-bis(decyloxy)benzyl)tributylstannane **12**[5] (7.87 g, 11.3 mmol, 3eq.) in anhydrous THF (90 mL) was cooled to -78°C. n-BuLi (4.53 mL, 11.3 mmol, 3eq. 2.5 M in hexane) was added dropwise under N_2. The mixture was stirred for another 40 min at -78°C. Then, CuCN·2LiCl solution (12.1 mL, 12.1 mmol, 3.2eq, 1M in THF) was added dropwise over 40 min under vigorous stirring. The resulting reaction mixture was allowed to reach -60°C and then, it was cooled down again to -78°C and a solution of **11** (0.56 g, 3.8 mmol, 1eq.) and freshly distilled TMSCl (1.44 mL, 11.3 mmol, 3eq.) in anhydrous THF (16 mL) was added dropwise. The reaction mixture was allowed to reach rt slowly and stirred at rt overnight. The reaction mixture was quenched with excess of 10% HCl while cooling in an ice bath and then further stirred at rt for 2h. The mixture was diluted with H_2O and extracted with ethyl acetate. The combined organic phases were stirred with large amount of H_2O which resulted in the precipitation of a white solid. After removing the solid by filtering through CELITE®, organic phase was separated, dried with Na_2SO_4, evaporated and subjected to flash chromatography. Elution with gradient eluent system (petrol ether/ethyl acetate 100/1, 50/1, 30/1, 25/1, 20/1) afforded 3.50 g (97%) of compound **5a** as colourless oil.

^{1}**H NMR** (400 MHz, $CDCl_3$) δ 6.30 (t, *J* = 4.0 Hz, 1H), 6.25 (d, *J* = 4.0 Hz, 2H), 3.89 (t, *J* = 8.0 Hz, 4H), 2.56-2.70 (m, 2H), 2.50 (s, 1H), 2.24-2.47 (m, 3H), 2.21 (s, 1H), 1.71-1.78 (m, 4H), 1.40-1.45 (m, 4H), 1.27-1.35 (m, 24H), 0.88 (t, *J* = 8.0 Hz, 6H); ^{13}**C NMR** (100 MHz, $CDCl_3$) δ 213.31, 160.58, 140.47, 107.61, 99.49, 68.14, 47.79, 41.82, 41.74, 37.86, 32.03, 29.72, 29.70, 29.55, 29.46, 29.41, 26.19, 23.22, 22.82, 14.26; **IR** ν_{max}/cm^{-1} 2923, 2853, 1699, 1596, 1157, 1057; **HRMS** (ESI) calcd. for ($[M+Na]^+$): $C_{63}H_{104}O_6Na$ 979.7731; Found: 979.7712; Anal. calcd. for $C_{63}H_{104}O_6$: C, 79.03; H, 10.95; Found: C, 79.02; H, 10.96;

Compound 5b

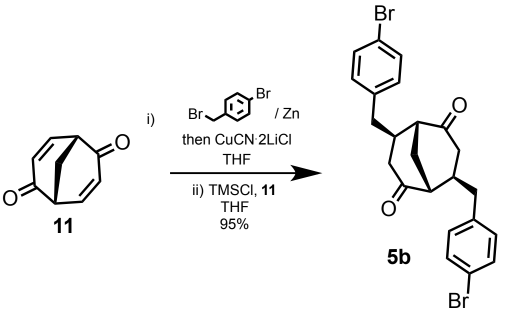

Zinc dust (468 mg, 7.2 mmol, 10 eq.) in THF (20 ml) was activated with dibromoethane (135 mg, 0.72 mmol, 1.0 eq.) five times by heating to boiling and cooling down to rt. TMSCl (50 µl, 0.36 mmol, 0.5 eq.) was then added and the mixture was stirred for 30 min at rt. To so obtained activated zinc, 4-bromobenzyl bromide (1.07 g, 4.3 mmol, 6.0 eq.) in THF (6 ml) was added dropwise slowly at 0°C. After addition, the mixture was stirred 20 min at 0°C. The stirring was stopped and grey solution was transferred via syringe to a solution of CuCN·2LiCl complex (4.3 ml 1.0 M in THF, 4.3 mmol, 6.0 eq.) in dry THF (8 ml) at -78°C. After addition, the mixture was warmed to -30°C, and then cooled back to-78°C. A solution of enone **11** (106 mg, 0.72 mmol, 1.0 eq.) and TMSCl (0.55 ml, 4.3 mmol, 6.0 eq.) in THF (4 ml) was added dropwise. The reaction mixture was allowed to reach rt slowly and stirred at rt overnight. The reaction mixture was quenched with excess of 10% HCl cooling with an ice bath and the resulting mixture was stirred at rt for 2h. Then, it was diluted with H_2O and extracted with ethyl acetate. To the combined organic phase large amount of H_2O was added, which resulted in the precipitation of a white solid. After removing the solid by filtering through CELITE®, organic phase was separated, dried with Na_2SO_4, evaporated and purified by flash chromatography (petrol ether/ethyl acetate 5/1) to give **5b** (332 mg, 95%) as a white solid.

m.p. 169-171°C

^{1}H NMR (400 MHz, $CDCl_3$) δ 7.45 (d, J = 8.2 Hz, 4H), 7.02 (d, J = 8.2 Hz, 4H), 2.73-2.62 (m, 4H), 2.48 (br. s., 2H), 2.46-2.35 (m, 4H), 2.29-2.22 (m, 2H), 2.20 (br. s., 2H); **^{13}C NMR** (100 MHz, $CDCl_3$) δ 212.6, 137.3, 131.9, 130.8, 120.8, 47.6, 41.6, 40.7, 37.8, 23.1; **IR** ν_{max}/cm^{-1} 2924, 1711, 1695, 1487, 1010; **HRMS** (ESI) calcd. for ($[M+H]^+$): $C_{23}H_{23}Br_2O_2$ 489.0065; Found: 489.0068;

Compound 14

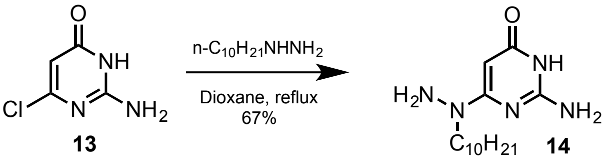

A mixture of 2-amino-6-chloropyrimidin-4-ol **13** (0.50 g, 11.1 mmol) and 1-decylhydrazine (1.4 mL) in dry dioxane (25 mL) was refluxed for 90 min. The contents from the flask were poured into H_2O (200 mL). The precipitate was filtered, washed with Et_2O and dried under reduced pressure to afford **14** as a brownish solid (0.65 g, 67%).

^{1}H NMR (300 MHz, DMSO-d_6) 9.67 (s, 1H), 6.14 (s, 2H), 4.97 (s, 1H), 4.36 (s, 1H), 3.53 (t, J = 7.5 Hz, 2H), 1.57–1.46 (m, 2H), 1.26 (m, 18H), 0.87 (t, J = 6.7 Hz, 3H); **^{13}C NMR** (75 MHz, DMSO-d_6) δ 166.2, 163.8, 154.9, 76.3, 50.6, 32.0, 29.8, 29.7, 29.4, 27.2, 27.0, 22.8, 14.7; **IR** ν_{max}/cm^{-1} 3496, 3305, 3170, 2923, 1697, 1646; **HRMS** (ESI) calcd. for ($[M+H]^+$): 282.2294; Found: 282.2284.

Compound 8a

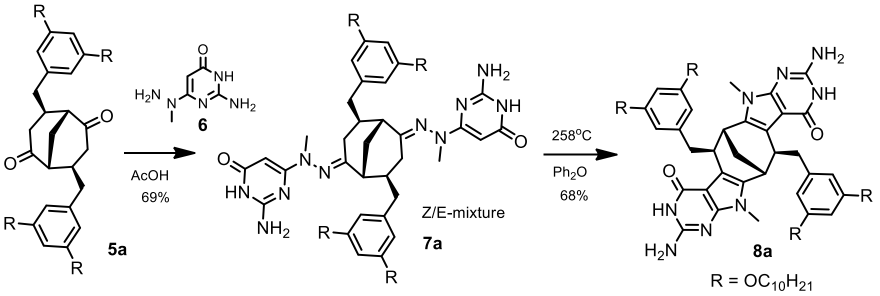

A suspension of compound **5a** (3.17 g, 3.31 mmol, 1.0 eq.) and 2-amino-6-(1-methylhydrazinyl)-4(3H)-pyrimidinone **6** (1.32 g, 8.51 mmol, 2.6 eq.) in acetic acid (185 mL) was stirred at rt. After 19 h, the solvent was removed under reduced pressure at 30°C. Toluene was used to remove residues of acetic acid. Then, it was dried under high vacuum overnight and subjected to flash chromatography. Elution with gradient elution system (CH_2Cl_2/MeOH + 1% Et_3N, 100/1, 60/1, 40/1, 30/1, 20/1) afforded 2.81 g (69% yield) of compound **7a**. Compound **7a** was obtained as a mixture of E and Z isomers which gave a very complicated ^{1}H NMR spectrum. For this reason, full characterization of **7a** was not attempted and the compound was used directly in the next step.

HRMS (ESI) calcd. for ([M+H]$^+$): $C_{73}H_{119}N_{10}O_6$ 1231.9314; Found: 1231.9343; Anal. calcd. for $C_{73}H_{118}N_{10}O_6$·1/6 CH_2Cl_2: C, 70.53; H, 9.57; N, 11.24; Found: C, 70.52; H, 9.56; N, 11.26;

A solution of compound **7a** (2.50 g, 2 mmol) in diphenyl ether (95 mL) was heated to reflux with an air condenser, passing argon through the mixture via a long cannula (Supplementary Fig. 2). After 29 h, the evolution of ammonia ceased and the reaction mixture was cooled to rt. The reaction mixture was directly loaded on the silica gel column and diphenyl ether was removed by using petroleum ether as eluent. Then, the eluent was changed to CH_2Cl_2/MeOH (10/1) to afford 2.34 g of partially purified product. The crude product was further purified by flash chromatography with gradient eluent system (CH_2Cl_2, then CH_2Cl_2/MeOH 100/1, 80/1, 50/1, 30/1, 20/1) to give 1.65 g (68% yield) of compound **8a** as a yellowish glass.

^{1}H NMR (400 MHz, $CDCl_3$) δ 12.90 (br. s, 1H), 6.60 (d, J = 4.0 Hz, 2H), 6.34 (s, 1H), 4.71 (s, 2H), 3.93 (t, J = 8.0 Hz, 4H), 3.52 (d, J = 8.0 Hz, 1H), 3.02-3.08 (m, 2H), 2.98 (s, 3H), 2.42 (t, J = 12 Hz, 1H), 2.08 (s, 1H), 1.72-1.79 (m, 4H), 1.40-1.44 (m, 4H), 1.26-1.31 (m, 24H), 0.87 (t, J = 8.0 Hz, 6H); **^{13}C NMR** (100 MHz, $CDCl_3$) δ 161.55, 160.39, 151.47, 151.12, 144.45, 131.5, 113.04, 108.40, 98.72, 98.57, 68.16, 41.59, 41.59, 32.03, 29.73, 29.71, 29.58, 29.54, 29.46, 27.66, 27.33, 26.24, 22.82, 21.37, 14.26; **IR** ν_{max}/cm^{-1} (in $CDCl_3$) 3507, 3406, 1664, 1634, 1596, 1460; **HRMS** (ESI) calcd. for ([M+H]$^+$): $C_{73}H_{113}N_8O_6$ 1197.8783; Found: 1197.8761; Anal. calcd. for $C_{73}H_{112}N_8O_6$·1/5 CH_2Cl_2: C, 72.38; H, 9.33; N, 9.22; Found: C, 72.54; H, 9.34; N, 8.83;

Compound 1

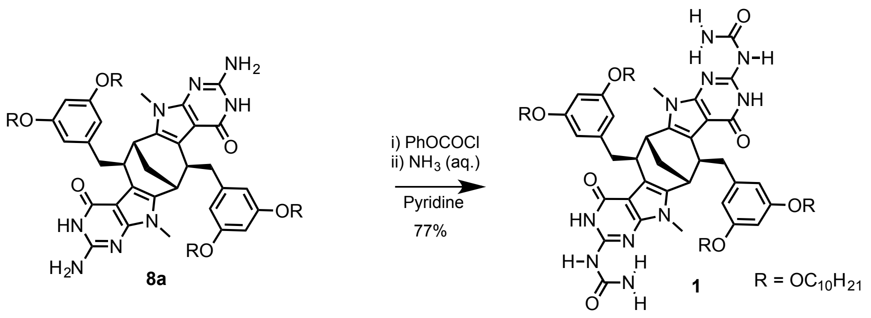

8a (0.53 g, 0.44 mmol, 1.0 eq.) was dissolved in anhydrous pyridine (30 mL) under N_2 and the solution obtained was cooled in an ice bath. Then PhOCOCl (0.61 mL, 4.86 mmol, 11.0 eq.) was added dropwise. After 10 min, the ice bath was removed and the reaction mixture was stirred at rt for 22h. Then it was cooled to 0 °C again and 25% NH_3·H_2O (2.8 mL) was added dropwise. The result mixture was allowed to reach rt and was stirred at rt overnight. The suspension was

quenched with excess of 10% HCl and extracted with CH_2Cl_2. The combined organic phase was washed with sat. $NaHCO_3$, dried with Na_2SO_4 and evaporated. The residue was triturated with MeOH (300 mL) and sonicated for several minutes. The suspension was filtered to give 0.43 g (77% yield) of compound **1** as an off-white solid.

^{1}H NMR (400 MHz, $CDCl_3$) δ 13.43 (s, 1H), 8.88 (s, 1H), 8.44 (s, 1H), 7.12 (s, 1H), 6.56 (s, 2H), 6.36 (s, 1H), 3.97 (br. s, 4H), 3.55 (d, *J* = 8.0 Hz, 1H), 3.28 (m, 4H), 3.02 (s, 1H), 2.72 (br. s, 1H), 1.81-1.84 (m, 5H), 1.15-1.45 (m, 28H), 0.80 (t, *J* = 8.0 Hz, 6H); **^{13}C NMR** (100 MHz, $CDCl_3$) δ 160.29, 160.01, 156.72, 147.80, 145.20, 143.46, 132.67, 114.01, 108.15, 101.35, 99.18, 68.09, 41.44, 40.18, 31.99, 29.87, 29.75, 29.70, 29.53, 29.43, 28.30, 28.30, 26.24, 22.77, 20.99, 14.18; **IR** ν_{max}/cm^{-1} (in $CDCl_3$) 3390, 3353, 1703, 1593, 1468, 1421; **HRMS** (ESI) calcd. for ($[M+H]^+$): $C_{75}H_{115}N_{10}O_8$ 1283.8899; Found: 1283.8911; Anal. calcd. for $C_{75}H_{114}N_{10}O_8$: C, 70.17; H, 8.95; N, 10.91; Found: C, 70.01; H, 8.92; N, 10.86;

Compound 8b

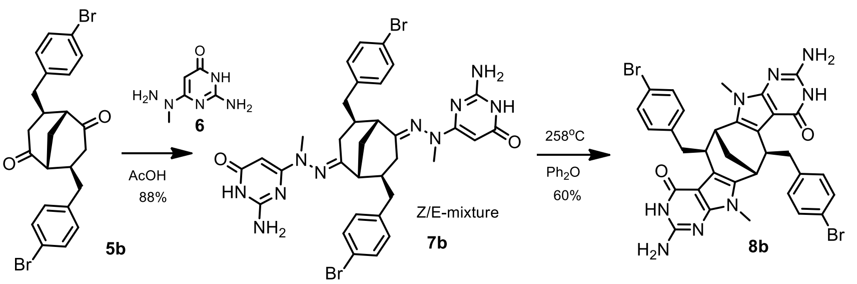

Mixture of **5b** (210 mg, 0.428 mmol, 1.0 eq.) and 2-amino-6-(1-methylhydrazinyl)pyrimidin-4(3H)-one (145 mg, 0.934 mmol, 2.2 eq.) in glacial acetic acid (11 ml) was stirred for 16 h at rt and then concentrated to dryness. Column chromatography on silica gel using gradient eluent system $CHCl_3$/MeOH/NH_4OH (100:8:1 to 100:6:1) afforded 288 mg (88%) of **7b** as a mixture of Z/E stereoisomers, which was used in the next step without further characterization.

A suspension of **7b** (288 mg) in diphenylether (8 ml) was refluxed for 10.5 h. with argon gas passing through the reaction mixture via long cannula (see Supplementary Fig. 2). After cooling to r.t., the crude product was precipitated with hexane (40 ml). Column chromatography on silica gel using gradient eluent system CH_2Cl_2/MeOH (20:1 -10:1 - 8:1) afforded 165 mg (60%) of **8b** as white solid.

m.p. 280°C (decomp.).

^{1}**H NMR** (400 MHz, d_6-DMSO) δ 10.13 (s, 2H), 7.56 (d, *J* = 8.0 Hz, 4H), 7.42 (d, *J* = 8.0 Hz, 4H), 6.13 (br s, 4H), 3.35–3.41 (m, 2H), 2.75–2.90 (m, 10H), 2.47–2.59 (m, 2H), 2.03 (br s, 2H); ^{13}**C NMR** (100 MHz, d_6-DMSO) δ 158.4, 152.0, 150.5, 141.0, 131.5, 131.1, 128.9, 119.0, 112.6, 97.2, 40.8, 26.9, 26.7; **IR** ν_{max}/cm^{-1} 3382, 2928, 2360, 1663, 1625, 1537, 1486, 1459, 1341, 1071, 1011, 831, 782, 574; **HRMS** (ESI) calcd. for ($[M+H]^+$): $C_{33}H_{31}Br_2N_8O_2$ 731.0916; found: 731.0908.

Compound 2

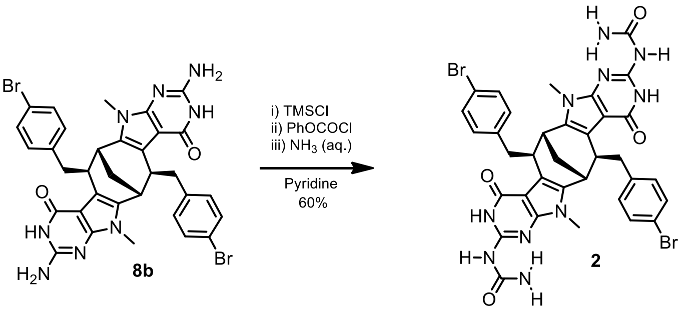

8b (42 mg, 0.057 mmol, 1.0 eq.) in freshly distilled pyridine (3 ml) was stirred with TMSCl (0.04 ml, 0.316 mmol, 5.5 eq.) for 3 h and then, phenyl chloroformate (0.04 ml, 0.318 mmol, 5.5 eq.) was added in one portion. The mixture was further stirred for 6 h. at r.t. 25% ammonia solution (2.5 ml) was added and the mixture was allowed to stir overnight. Reaction mixture was cooled in an ice bath and quenched first with 1M HCl solution and then with conc. HCl. The product was extracted with $CHCl_3$ (containing few % of MeOH), dried and concentrated in vacuum. Column chromatography on silica gel DCM/MeOH/NH_4OH (110:15:1) afforded 33 mg (70%) of **2** as an off-white solid.

m.p. 290°C (decomp.).

^{1}**H NMR** (400 MHz, d_6-DMSO) δ 11.52 (s, 2H), 9.70 (s, 2H), 7.58 (d, *J* = 8.0 Hz, 4H), 7.44 (d, *J* = 8.0 Hz, 4H), 7.06 (very br s, 2H), 6.42 (very br s, 2H), 3.34-3.42 (m, 2H), 2.82-3.01 (m, 10H), 2.59 (t, *J* = 12.0 Hz, 2H), 2.08 (br s, 2H); ^{13}**C NMR** (100 MHz, d_6-DMSO) δ 156.7, 156.0, 147.9, 146.8, 140.7, 131.4, 131.2, 130.9, 119.1, 113.1, 100.0, 40.7, 27.0, 26.8; **IR** ν_{max}/cm^{-1} 3346, 2932, 1710, 1670, 1593, 1407, 1249, 1069, 1011, 803, 780, 586; **HRMS** (ESI) calcd. for ($[M+H]^+$): $C_{35}H_{33}N_{10}O_4$ 817.1033; Found: 817.1029;

Compound 8c

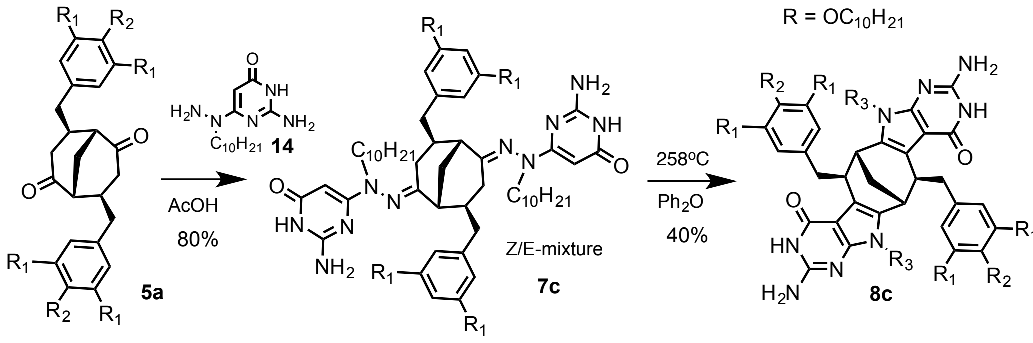

Mixture of **5a** (140 mg, 0.146 mmol, 1.0 eq.) and 2-amino-6-(1-decylhydrazinyl)pyrimidin-4(3H)-one **14** (90 mg, 0.321 mmol, 2.2 eq.) in glacial acetic acid (5.0 ml) was stirred for 16 h at rt and then concentrated to dryness. The crude mixture was dissolved in minimum amount of chloroform and diluted with methanol. The precipitate formed was filtered and used in the next step without further purification. Yield: 174 mg (80%).

7c (150 mg) in diphenyl ether (8 ml) was refluxed for 6 h. with argon gas passing through the reaction mixture via long cannula (see Supplementary Fig. 2). After cooling to rt, the reaction mixture was directly loaded on the silica gel column and diphenyl ether was removed by using petroleum ether as eluent. Then, the eluent was changed to CH_2Cl_2/MeOH (50/1) and elution was continued to afford **8c** (59 mg, 40%) as a yellowish glass.

^{1}H NMR (400 MHz, $CDCl_3$) δ 12.62 (br. s 2H), 6.62 (s, 4H), 6.34 (s, 2H), 4.72 (br. s, 4H), 3.93 (t, *J* = 6.5 Hz, 8H), 3.82-3.65 (m, 2H), 3.62-3.43 (m, 2H), 3.10 (d, *J* = 10.7 Hz, 2H), 3.06-2.90 (br. m, 4H), 2.43 (t, *J* = 11.2 Hz, 2H), 2.11 (br. s, 4H), 1.87-1.68 (m, 8H), 1.51-1.05 (m, 86H), 0.97-0.77 (m, 18H); **^{13}C NMR** (100 MHz, $CDCl_3$) δ 161.3, 160.4, 150.9, 150.8, 144.4, 130.7, 113.1, 108.1, 98.7, 98.6, 68.0, 41.7, 32.0, 31.9, 30.6, 29.76, 29.73, 29.72, 29.62, 29.59, 29.49, 29.48, 29.46, 29.45, 29.3, 27.6, 26.7, 26.1, 22.72, 22.68, 14.10, 14.09; **IR** ν_{max}/cm^{-1} 2924, 2854, 1668, 1594, 1462, 1163; **HRMS** (ESI) calcd. for ($[M+H]^+$): $C_{91}H_{149}N_8O_6$ 1451.1627; found: 1451.1624.

Compound 3

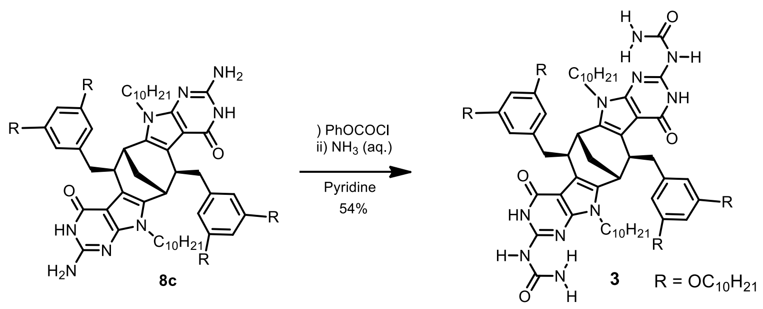

8c (70 mg, 0.046 mmol, 1.0 eq.) was dissolved in anhydrous pyridine (3.0 mL) under Ar. Then PhOCOCl (43 mg, 0.28 mmol, 6.0 eq.) was added at once at rt. The reaction mixture was stirred at rt for 5 h. Then, 25% $NH_3 \cdot H_2O$ (0.6 mL) was added dropwise. The result mixture was allowed to stir at rt overnight. The suspension was quenched with excess of 10% HCl and extracted with CH_2Cl_2. The combined organic phase was washed with sat. $NaHCO_3$, dried with Na_2SO_4 and evaporated. The residue was triturated with MeOH and sonicated for several minutes. The suspension was filtered to give 57 mg (77% yield) of compound **3** as an off-white solid.

^{1}H NMR (400 MHz, $CDCl_3$) δ 13.42 (s 2H), 8.86 (s, 2H), 8.44 (s, 2H), 7.12 (s, 2H), 6.52 (s, 4H), 6.37 (s, 2H), 3.95 (br. s, 10H), 3.64-3.3.4 (m, 4H), 3.26 (s, 2H), 3.00 (s, 2H), 2.87 (s, 2H), 1.86 (s, 8H), 2.00-1.67 (m, 8H), 1.67-0.96 (m, 82H), 0.96-0.68 (m, 18H); **^{13}C NMR** (100 MHz, $CDCl_3$) δ; 160.4, 160.3, 160.0, 156.6, 147.3, 144.9, 143.2, 132.5, 113.6, 107.9, 101.5, 99.0, 68.0, 42.4, 41.7, 40.5, 32.0, 31.93, 31.89, 30.6, 29.8, 29.71, 29.69, 29.59, 29. 55, 29.53, 29.43, 29.40, 29.37, 28.6, 26.7, 22.7, 22.65, 14.10. 14.05; **HRMS** (ESI) calcd. for ($[M+H]^+$): $C_{93}H_{151}N_{10}O_8$ 1536.1711; found: 1536.1718.

Compound 4

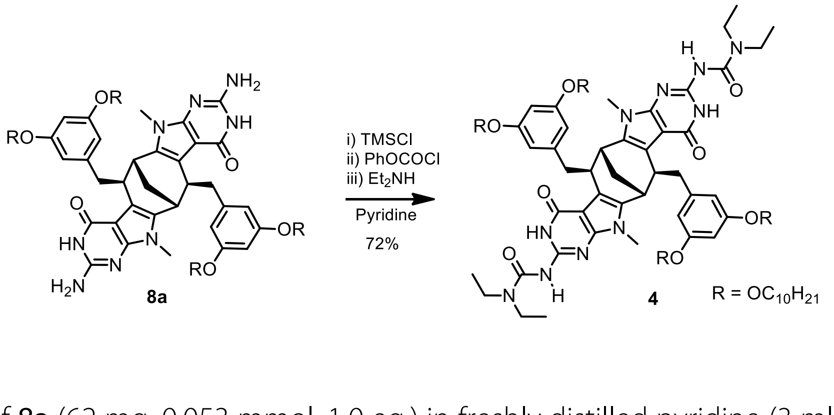

To a solution of **8a** (62 mg, 0.053 mmol, 1.0 eq.) in freshly distilled pyridine (3 ml) TMSCl (40 µl, 0.32 mmol, 6 eq.) was added at rt. The mixture was stirred for 2.5 h and then, phenyl chloroformate (40 µl, 0.32 mmol, 6.0 eq.) was added in one portion. After the mixture had

been stirred for 6 h, diethylamine (274 µl, 2.65 mmol, 50.0 eq.) was added and the orange mixture was stirred overnight. The reaction mixture was quenched with 1M HCl and extracted with dichloromethane. The combined organic phases were dried with Na_2SO_4, filtered and concentrated in vacuo. Purification of the crude product by flash chromatography (CH_2Cl_2/MeOH 60/1) afforded **4** (53 mg, 72%) as a yellowish glass.

^{1}H NMR (400 MHz, $CDCl_3$) δ 11.80 (s, 2H), 7.05 (s, 2H), 6.68 (d, *J* = 2.1 Hz, 4H), 6.37 (t, *J* = 2.1 Hz, 2H), 3.98 (td, *J* = 6.5, 2.2 Hz, 8H), 3.64 (dd, *J* = 12.9, 2.6 Hz, 2H), 3.36 (q, *J* = 7.1 Hz, 4H), 3.19 (dd, *J* = 11.3, 2.6 Hz, 2H), 2.45 (t, *J* = 12.9 Hz, 2H), 2.13 (br. t, *J* = 2.9 Hz, 2H), 1.81-1.74 (m, 4H), 1.52-1.42 (m, 4H), 1.41-1.26 (m, 28H), 1.23 (t, *J* = 7.1 Hz, 6H), 0.89 (t, *J* = 7.0 Hz, 6H); **^{13}C NMR** (100 MHz, $CDCl_3$) δ 160.3, 157.6, 153.5, 147.9, 146.2, 144.1, 132.0, 114.0, 108.0, 101.6, 99.3, 68.1, 41.7, 41.4, 40.8, 31.9, 29.61, 29.59, 29.48, 29.42, 29.35, 27.5, 26.1, 22.7, 14.1, 13.7; **IR** ν_{max}/cm^{-1} 3180, 2926, 2854, 1660, 1597, 1459, 1266, 1162; **HRMS** (ESI) calcd. for ($[M+H]^+$): $C_{83}H_{131}N_{10}O_8$ 1396.0151; found: 1396.0164.

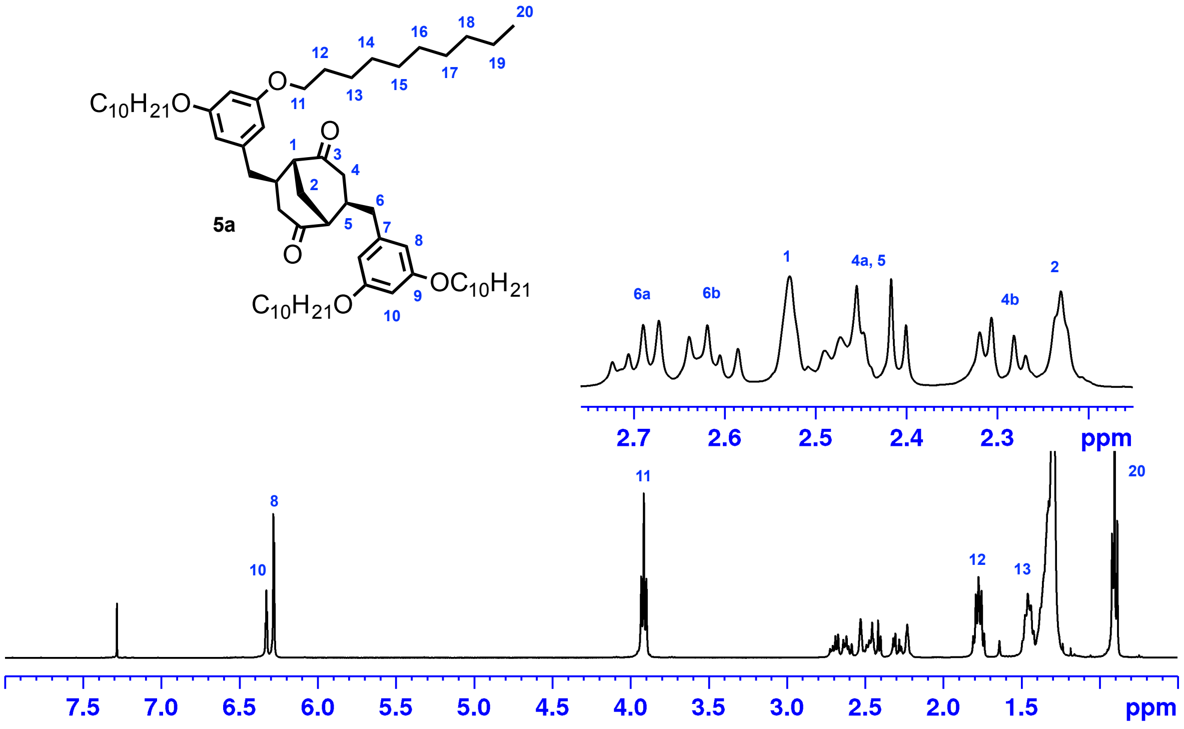

Supplementary Figure 4. ^{1}H NMR spectrum of **5a** in $CDCl_3$.

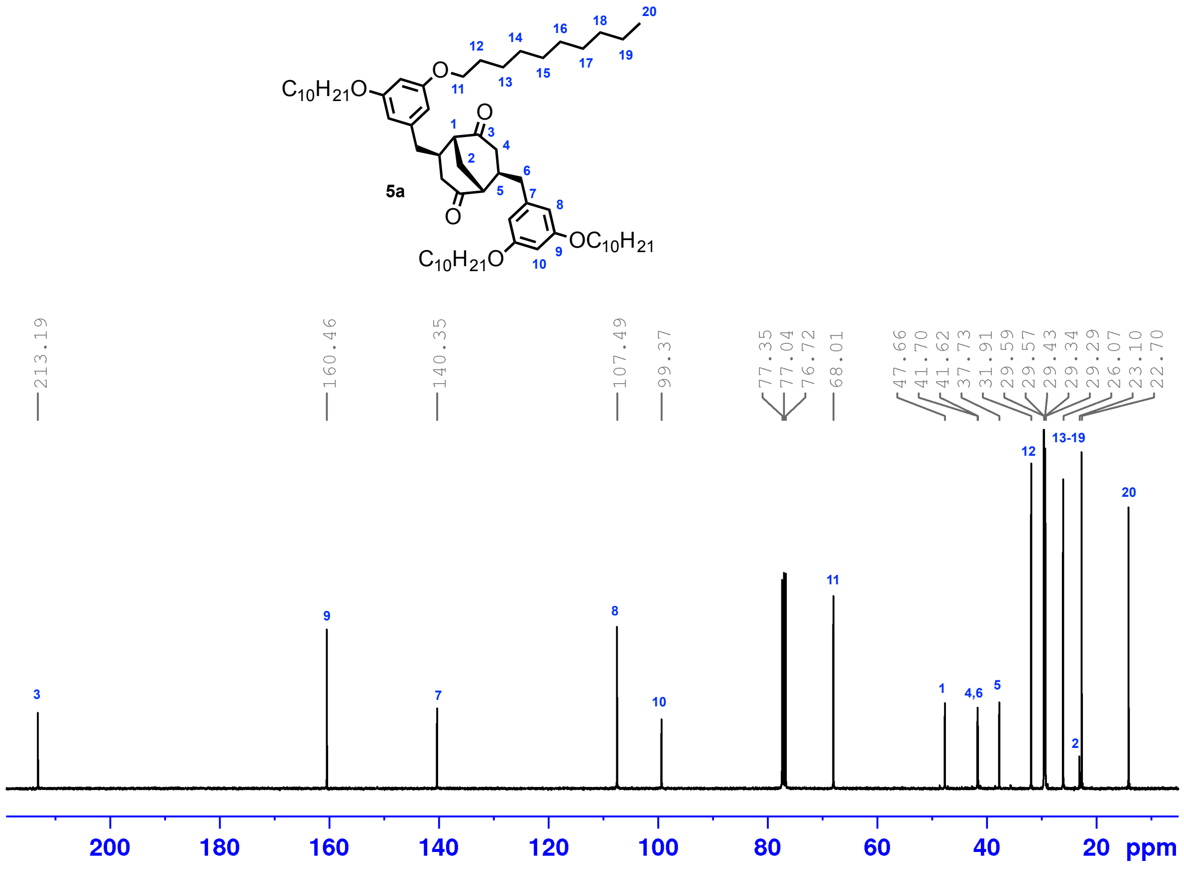

Supplementary Figure 5. ^{13}C NMR spectrum of **5a** in $CDCl_3$.

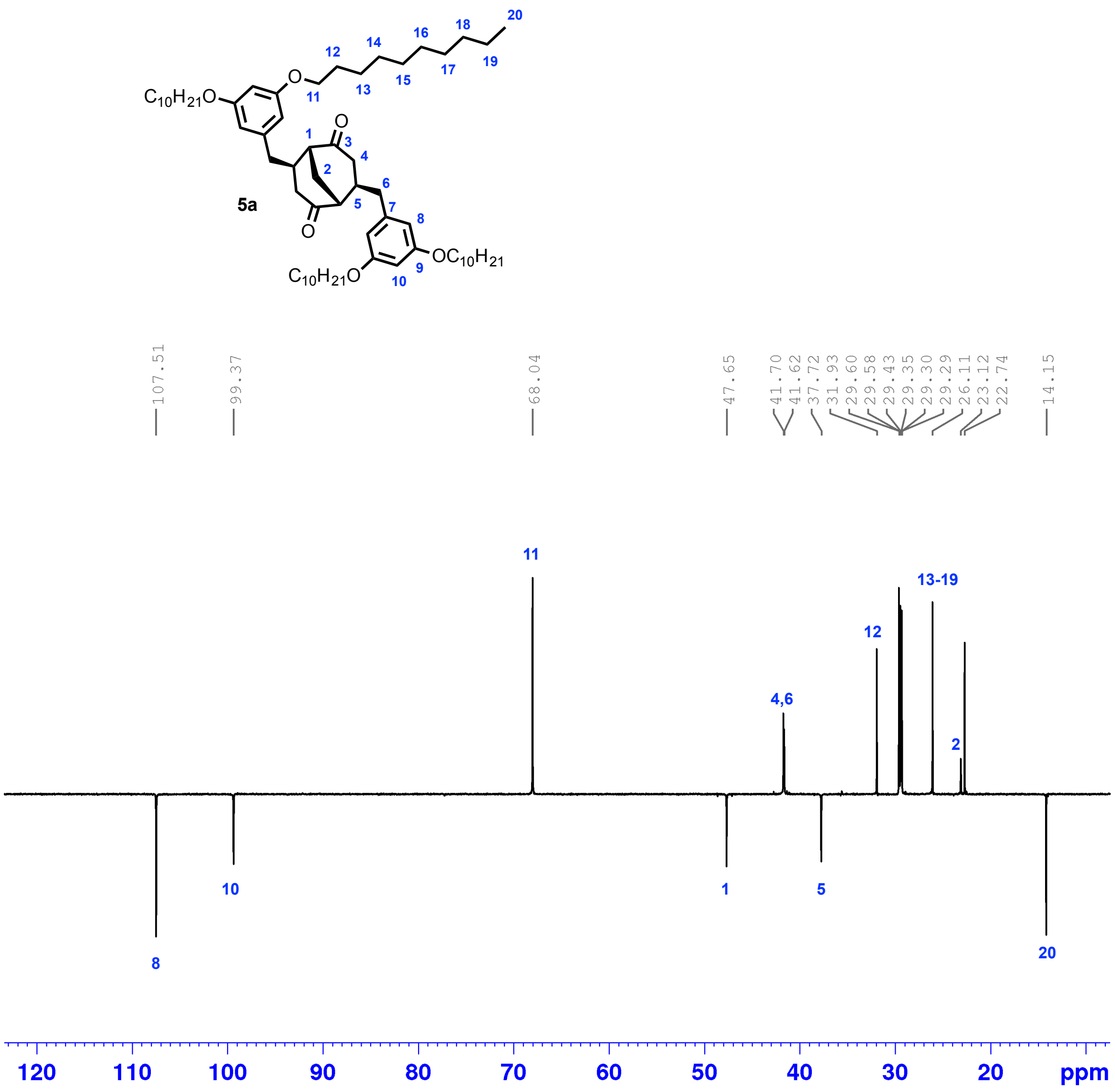

Supplementary Figure 6. DEPT spectrum of **5a** in $CDCl_3$.

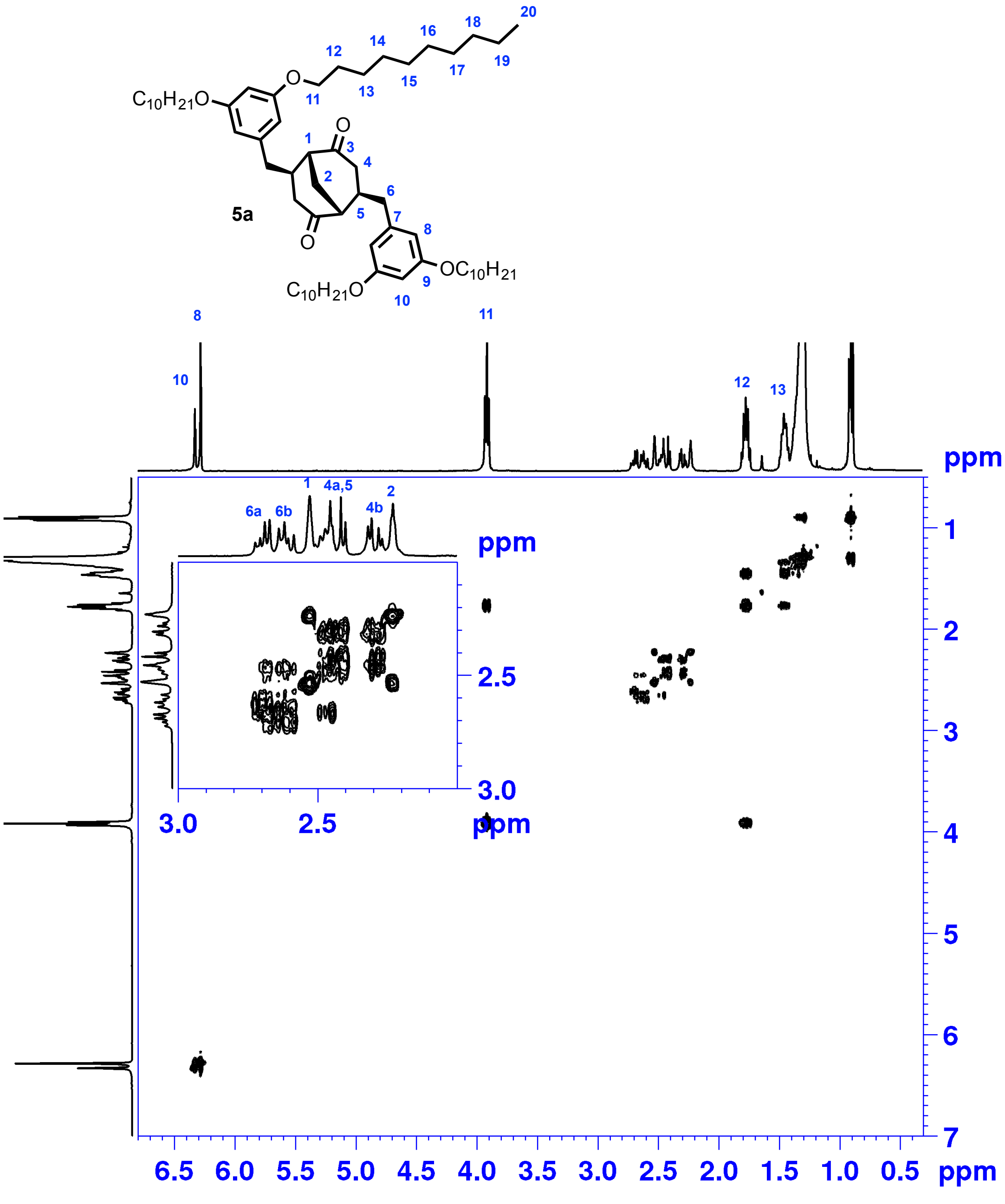

Supplementary Figure 7. COSY spectrum of **5a** in $CDCl_3$.

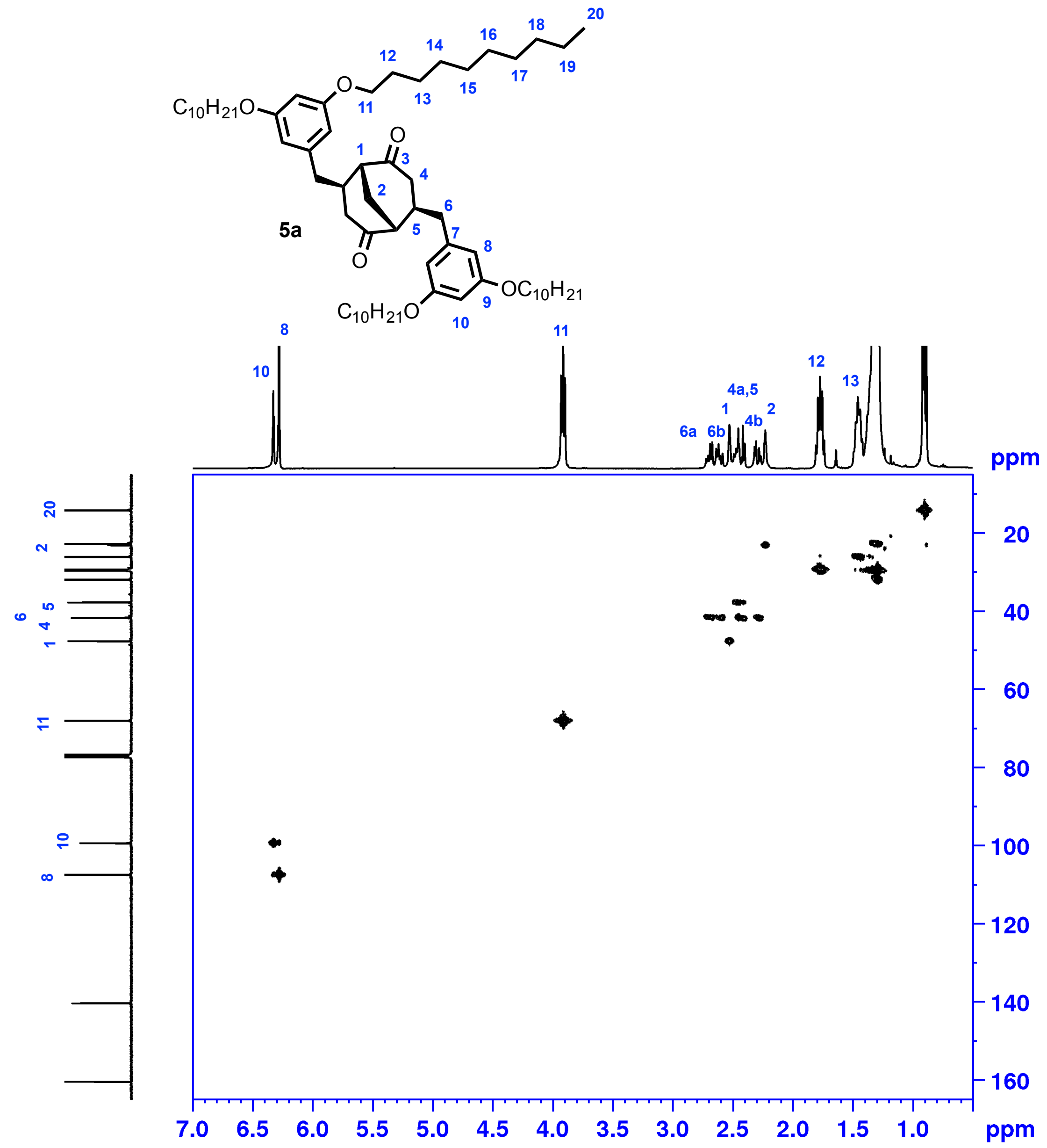

Supplementary Figure 8. ^{1}H-^{13}C HMQC spectrum of **5a** in $CDCl_3$.

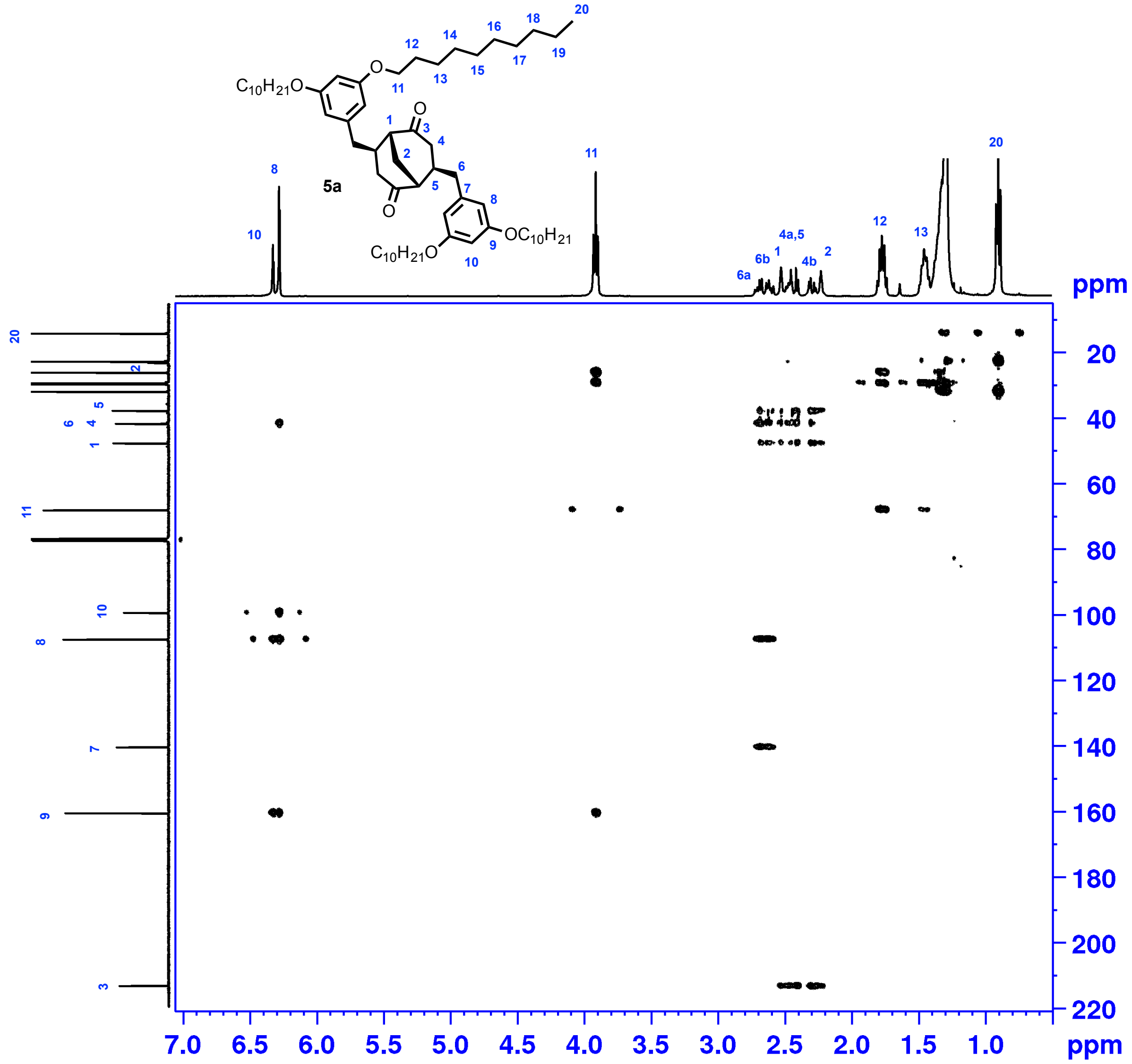

Supplementary Figure 9. HMBC spectrum of **5a** in $CDCl_3$.

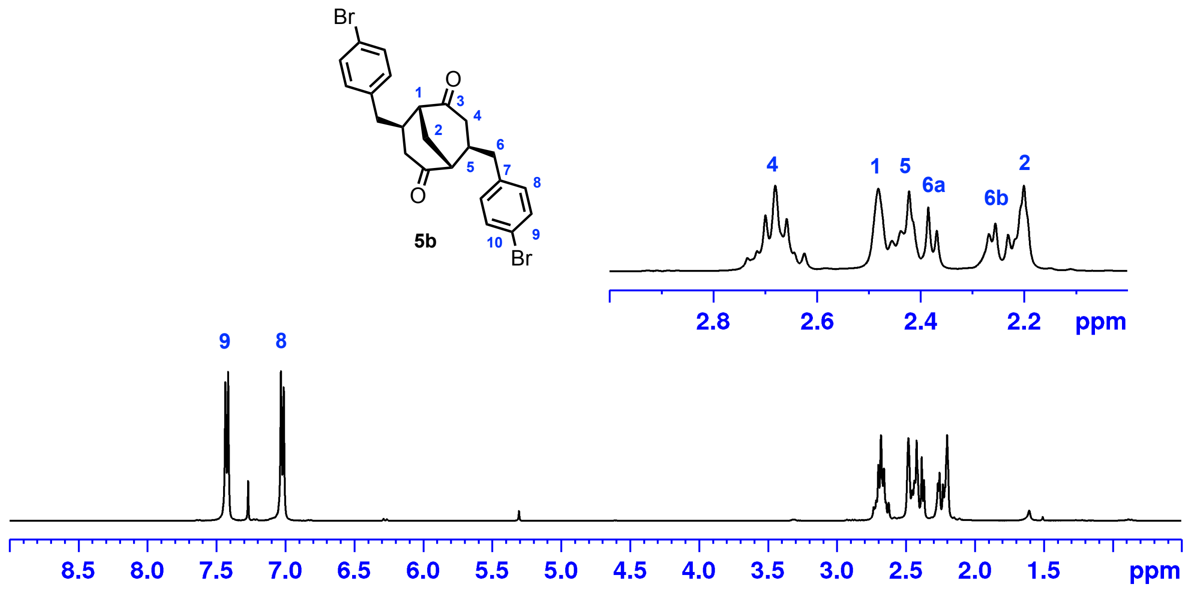

Supplementary Figure 10. ^{1}H NMR spectrum of **5b** in $CDCl_3$.

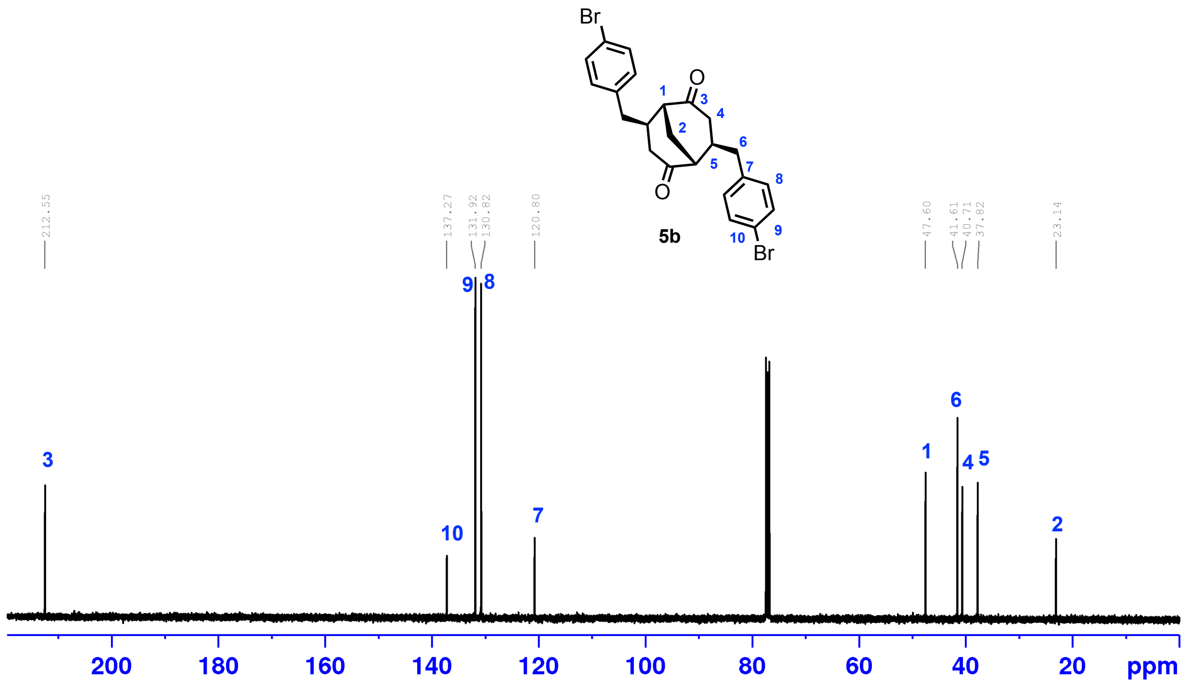

Supplementary Figure 11. ^{13}C NMR spectrum of **5b** in $CDCl_3$.

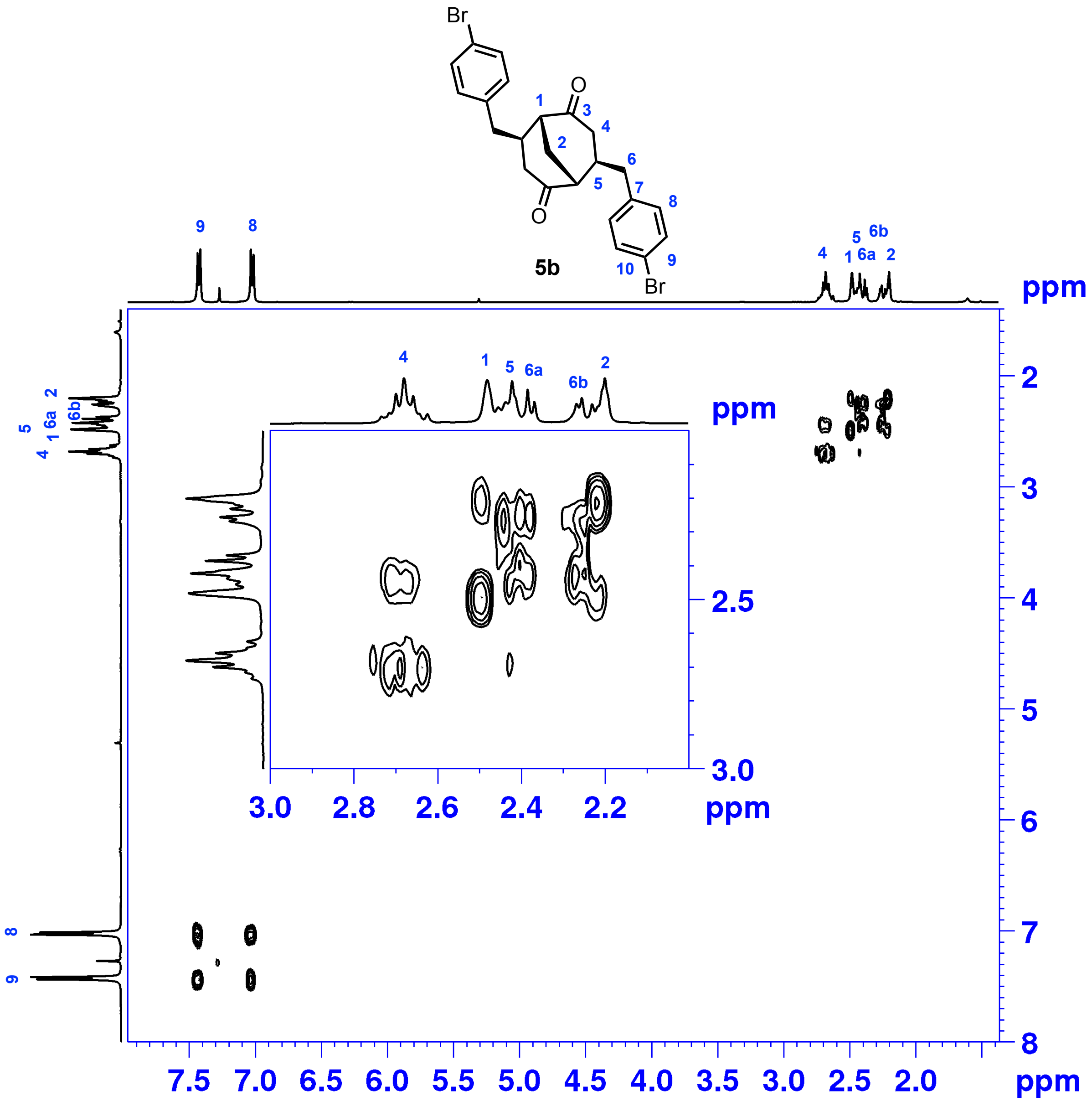

Supplementary Figure 12. COSY NMR spectrum of **5b** in $CDCl_3$.

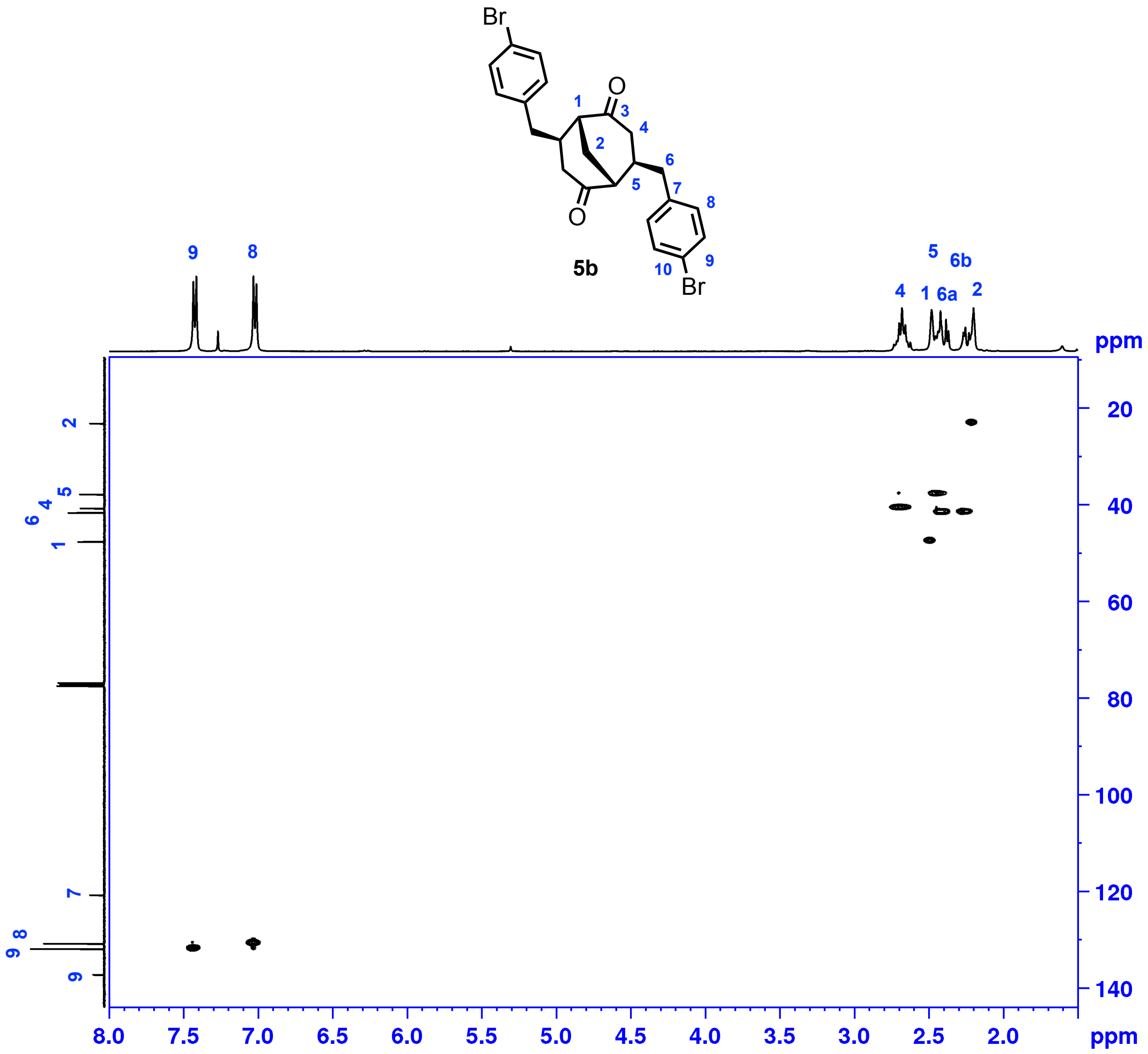

Supplementary Figure 13. ^{1}H-^{13}C HMQC spectrum of **5b** in $CDCl_3$.

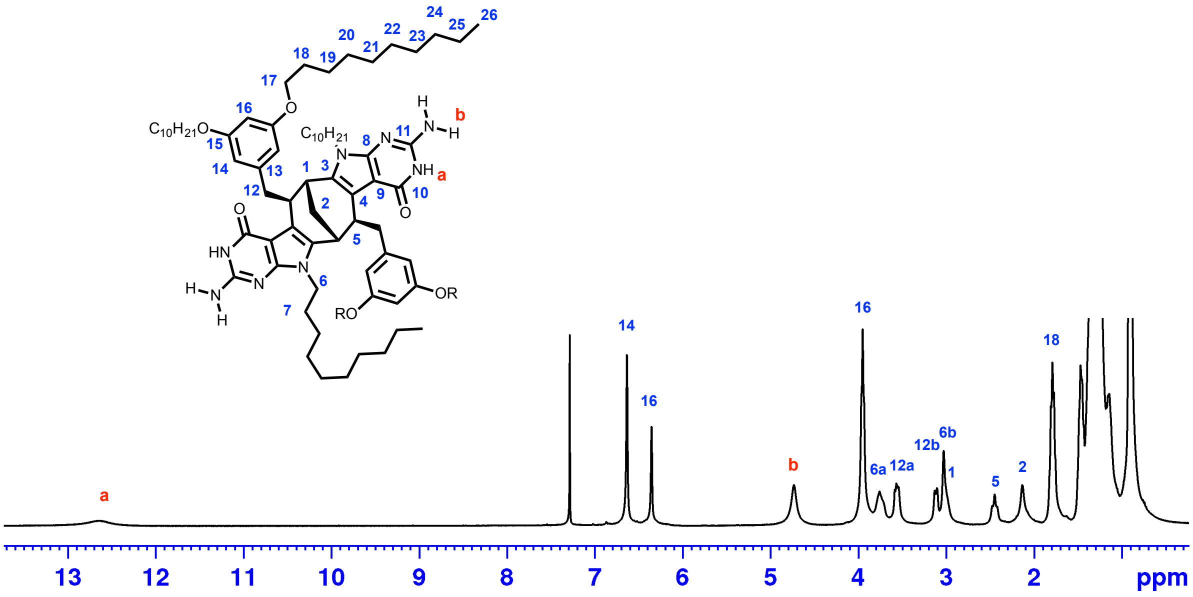

Supplementary Figure 14. ^{1}H NMR spectrum of **8c** in $CDCl_3$

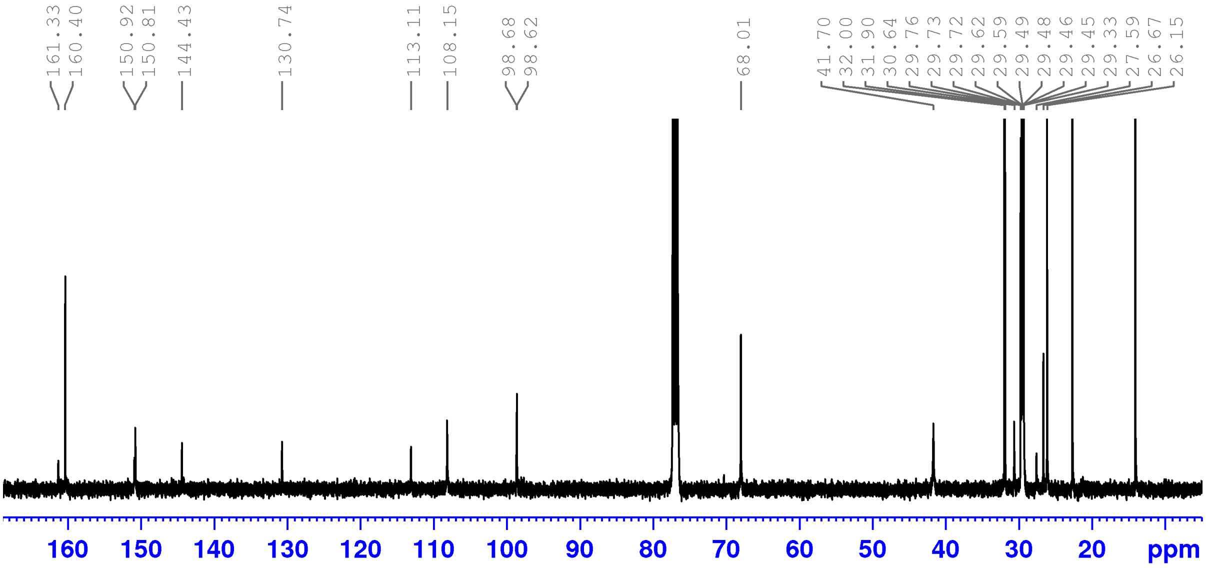

Supplementary Figure 15. ^{13}C NMR spectrum of **8c** in $CDCl_3$.

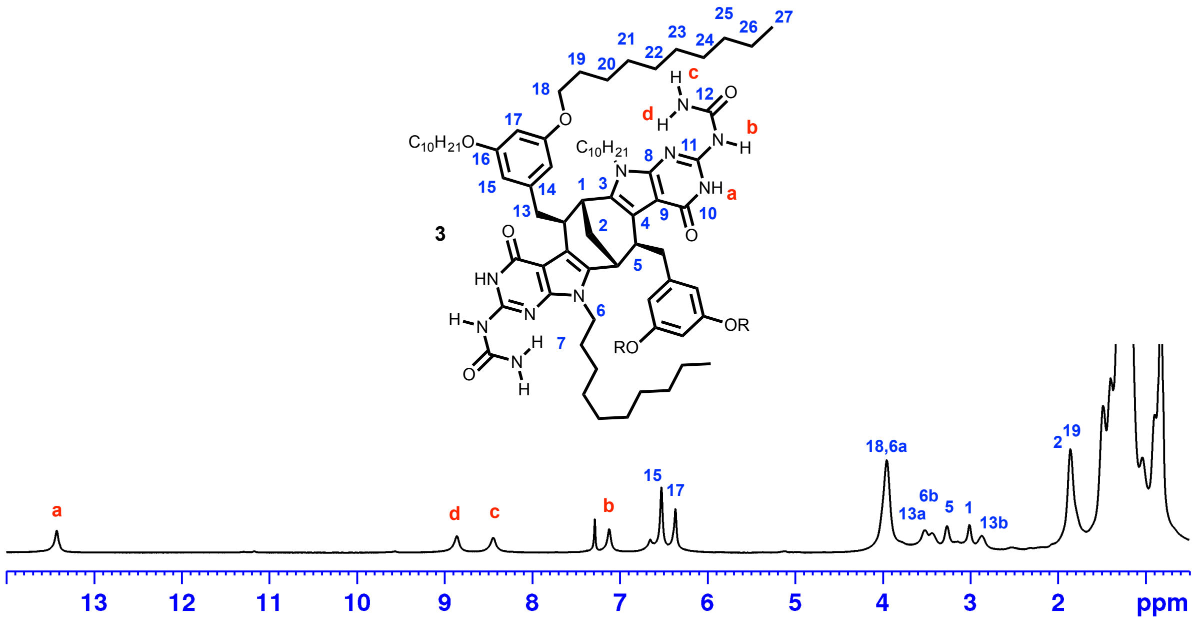

Supplementary Figure 16. ^{1}H NMR spectrum of **3** in $CDCl_3$.

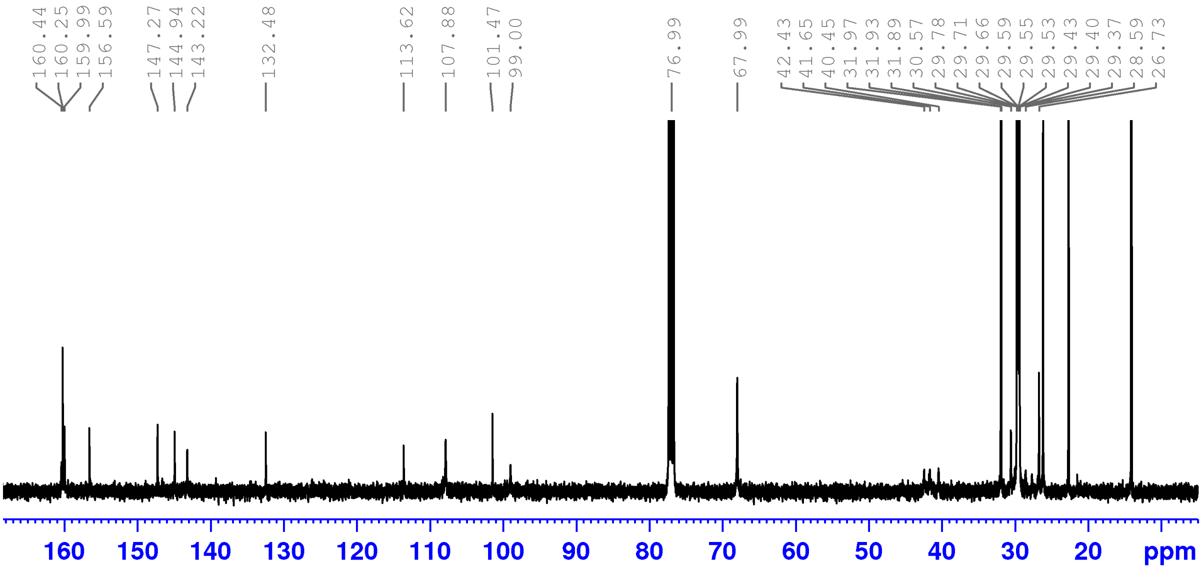

Supplementary Figure 17. ^{13}C NMR spectrum of **3** in $CDCl_3$.

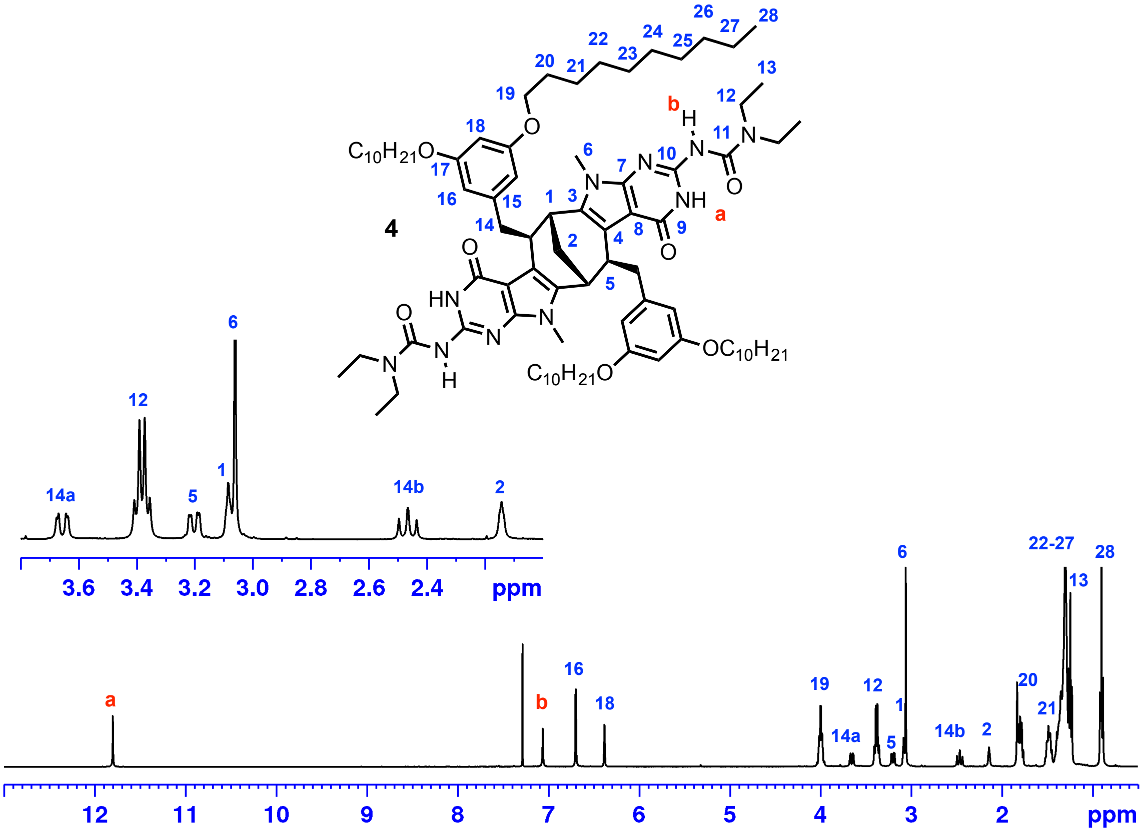

Supplementary Figure 18. ^{1}H NMR spectrum of **4** in $CDCl_3$.

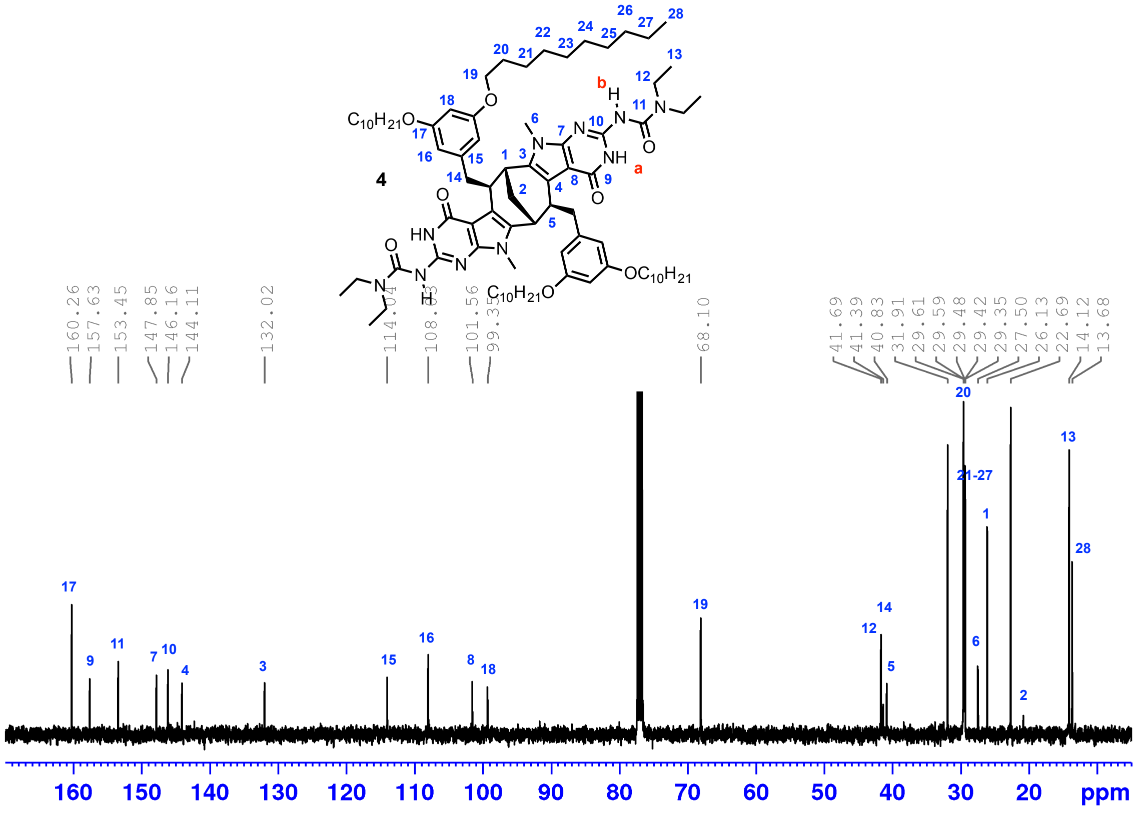

Supplementary Figure 19. ^{13}C NMR spectrum of **4** in $CDCl_3$.

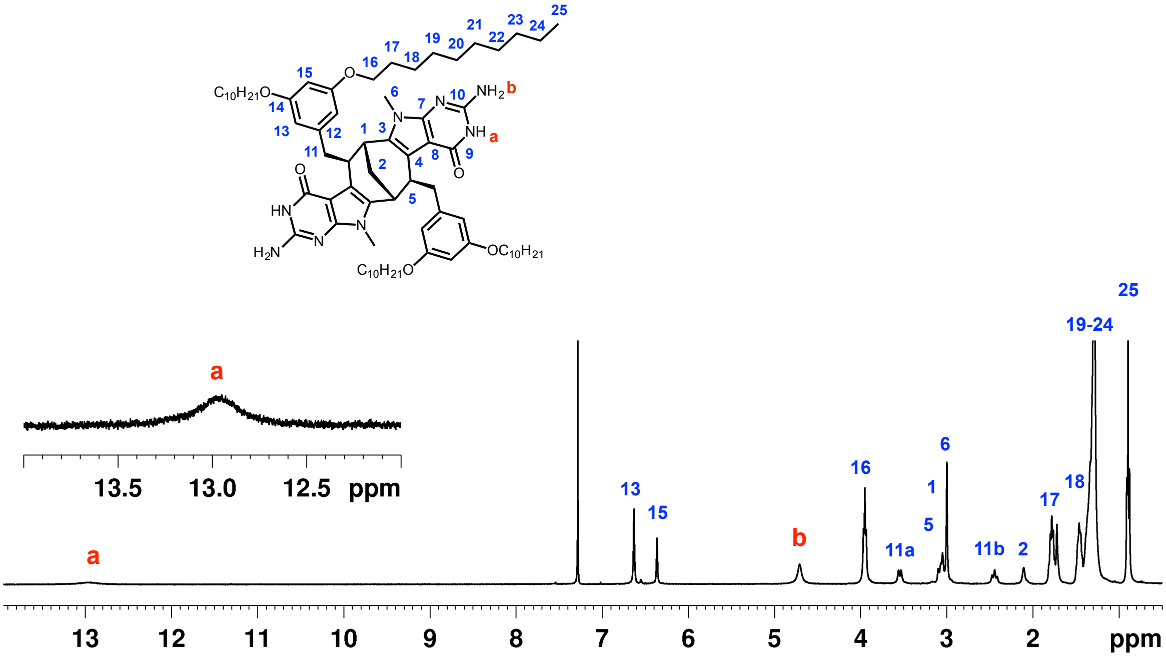

Supplementary Figure 20. ^{1}H NMR spectrum of **8a** in $CDCl_3$.

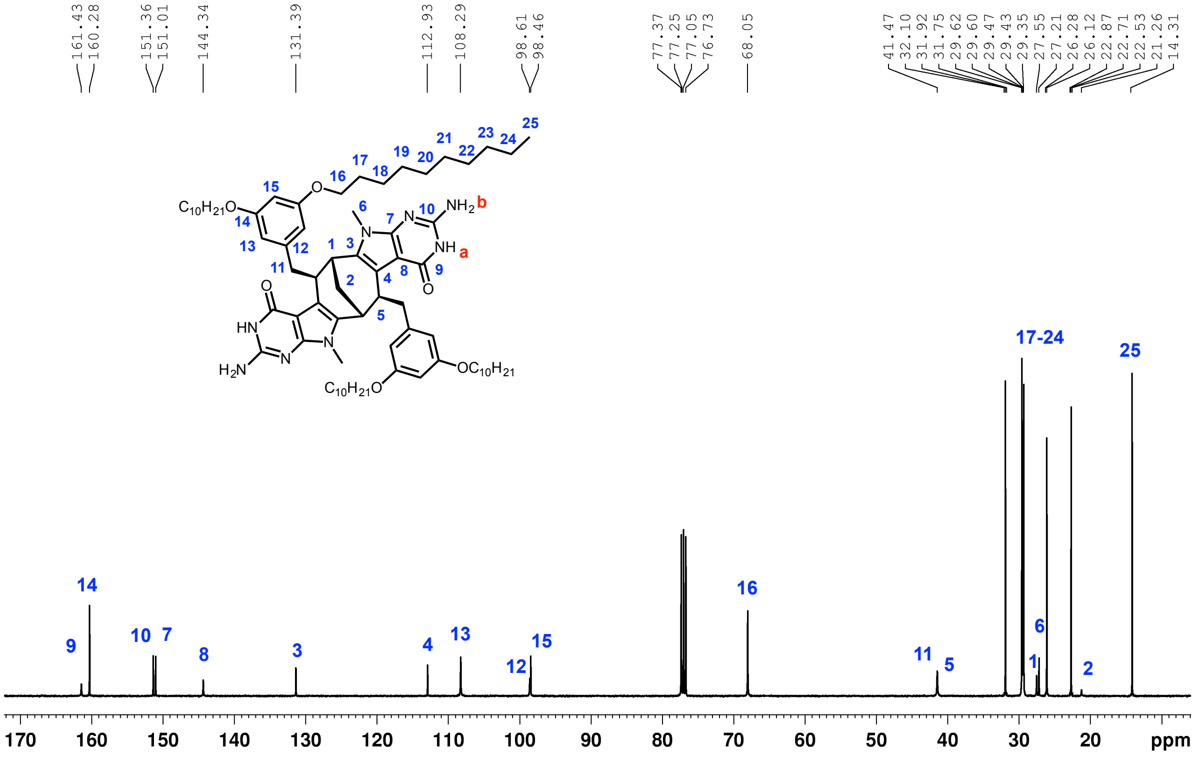

Supplementary Figure 21. ^{13}C NMR spectrum of **8a** in $CDCl_3$.

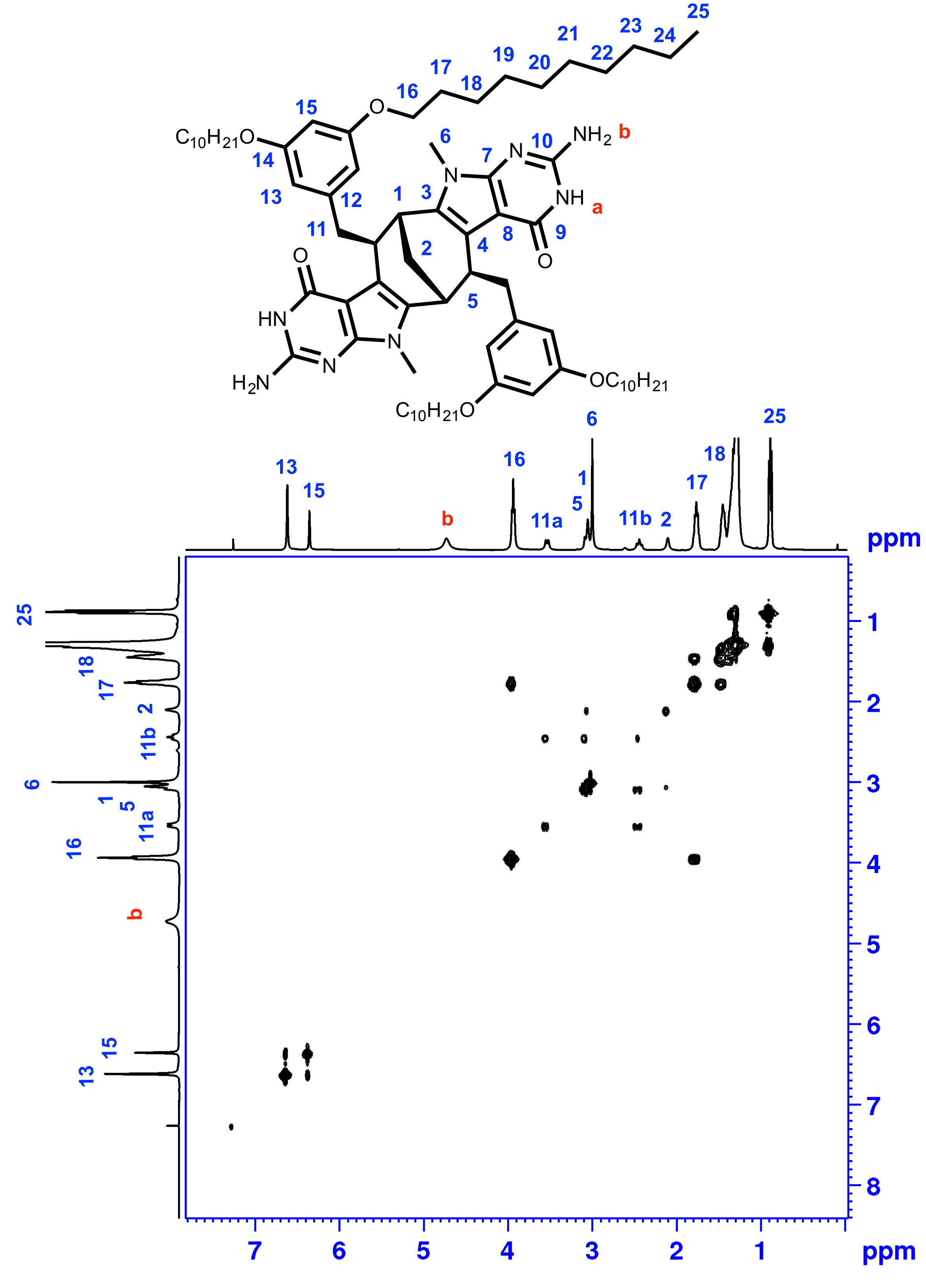

Supplementary Figure 22. COSY spectrum of **8a** in $CDCl_3$.

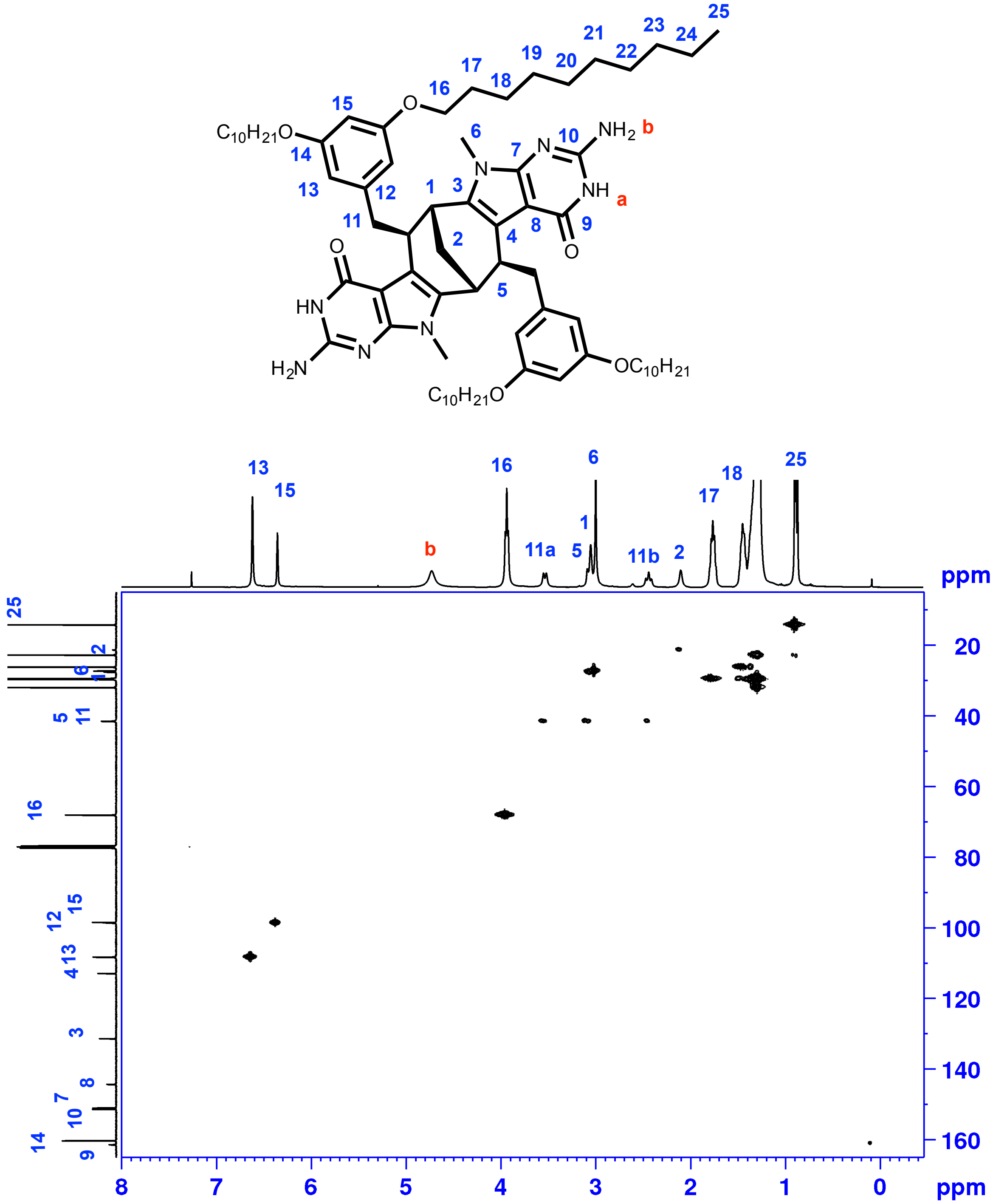

Supplementary Figure 23. ^{1}H-^{13}C HMQC spectrum of **8a** in $CDCl_3$.

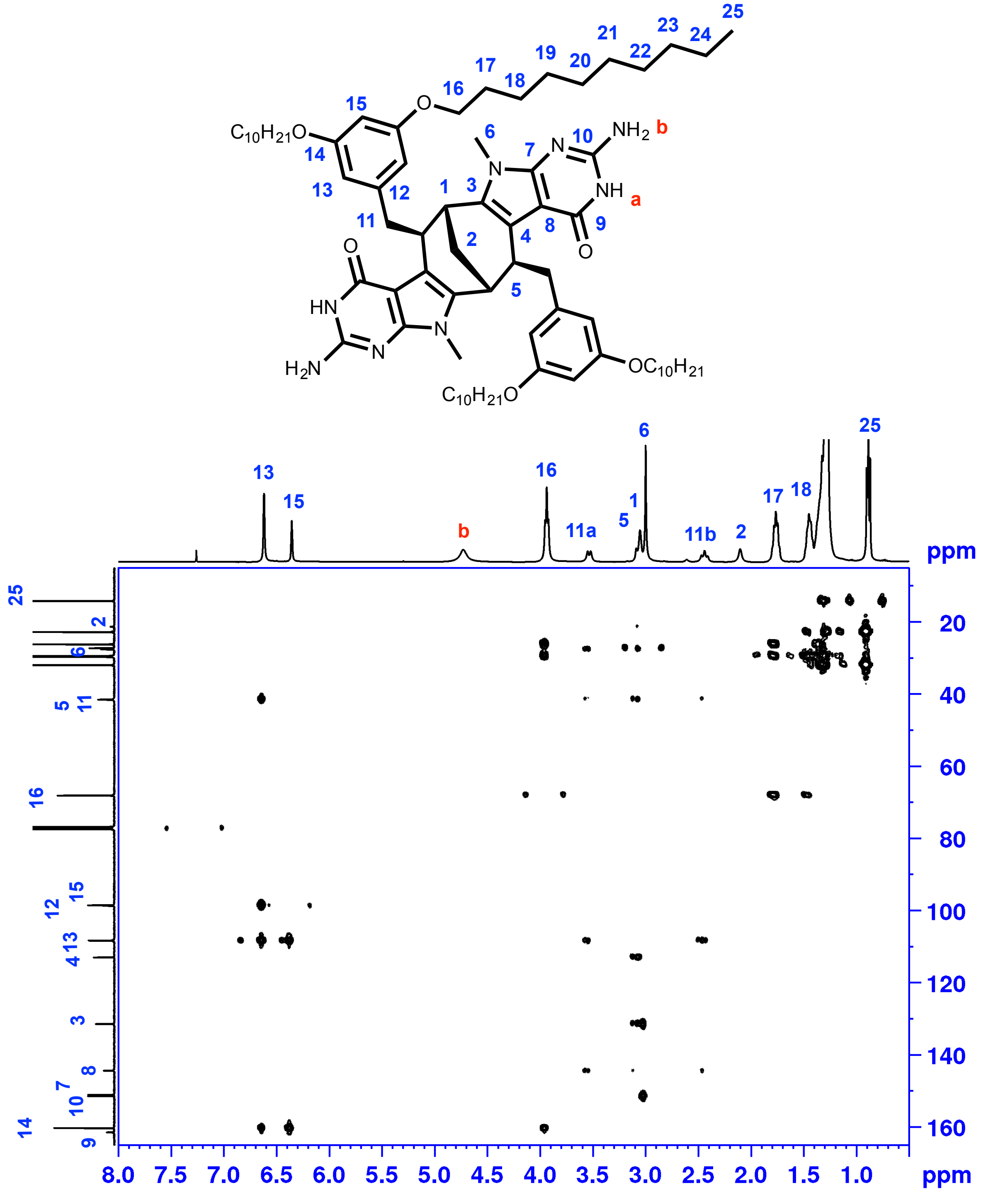

Supplementary Figure 24. HMBC spectrum of **8a** in $CDCl_3$.

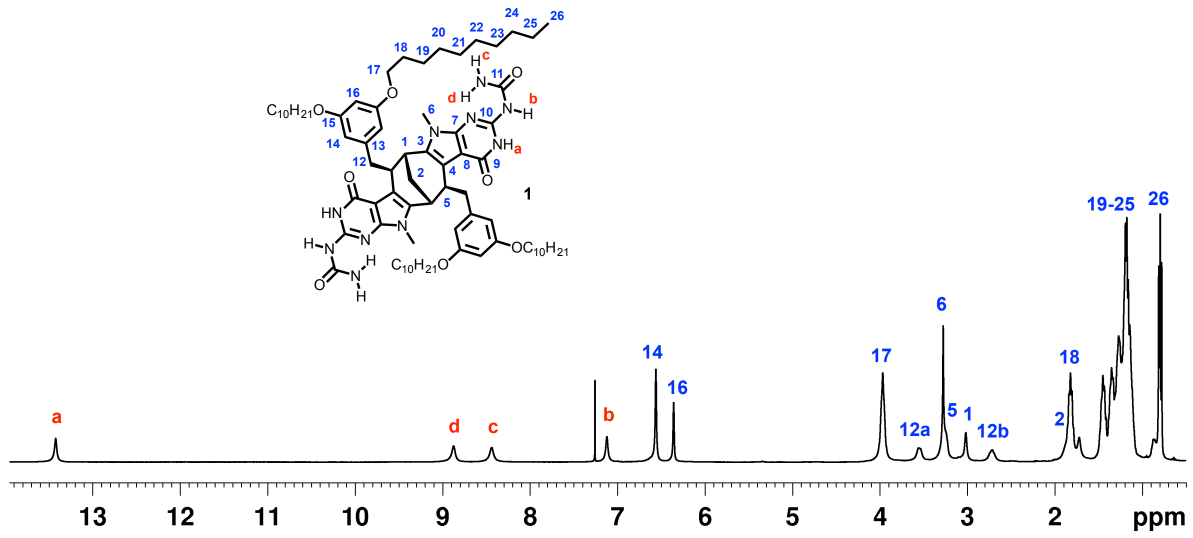

Supplementary Figure 25. ^{1}H NMR spectrum of **1** in $CDCl_3$.

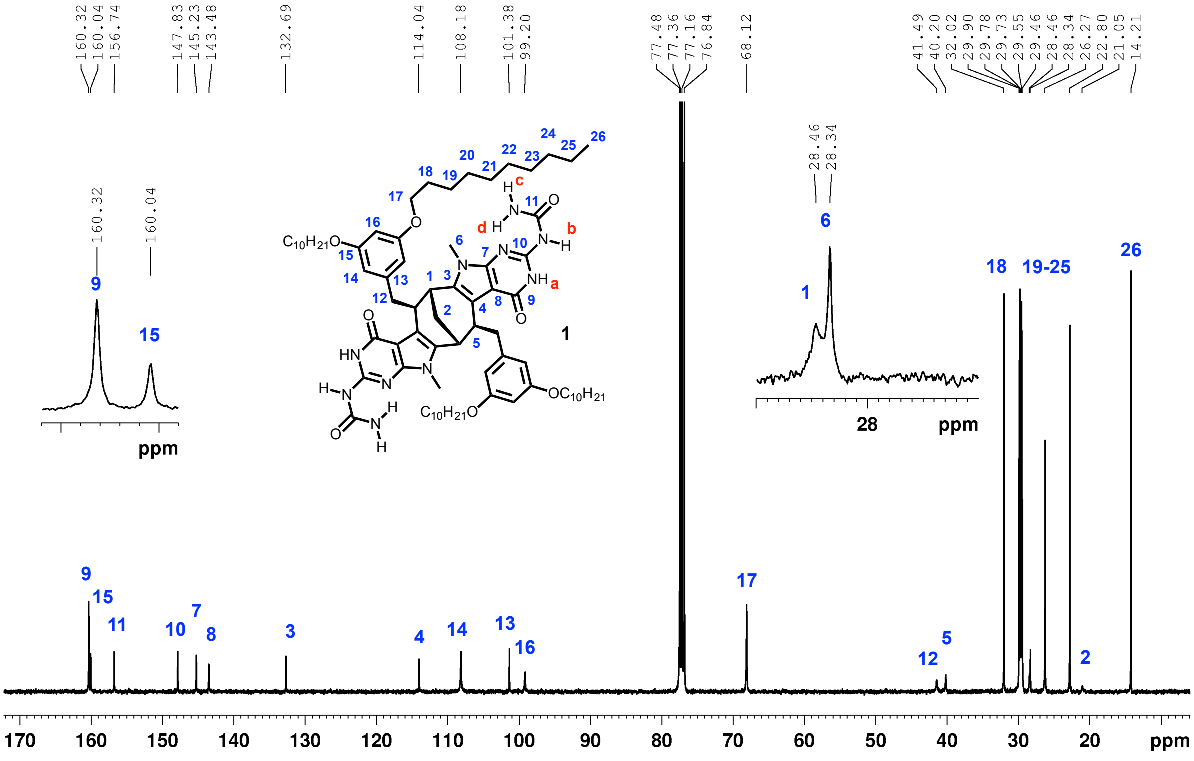

Supplementary Figure 26. ^{13}C NMR spectrum of **1** in $CDCl_3$.

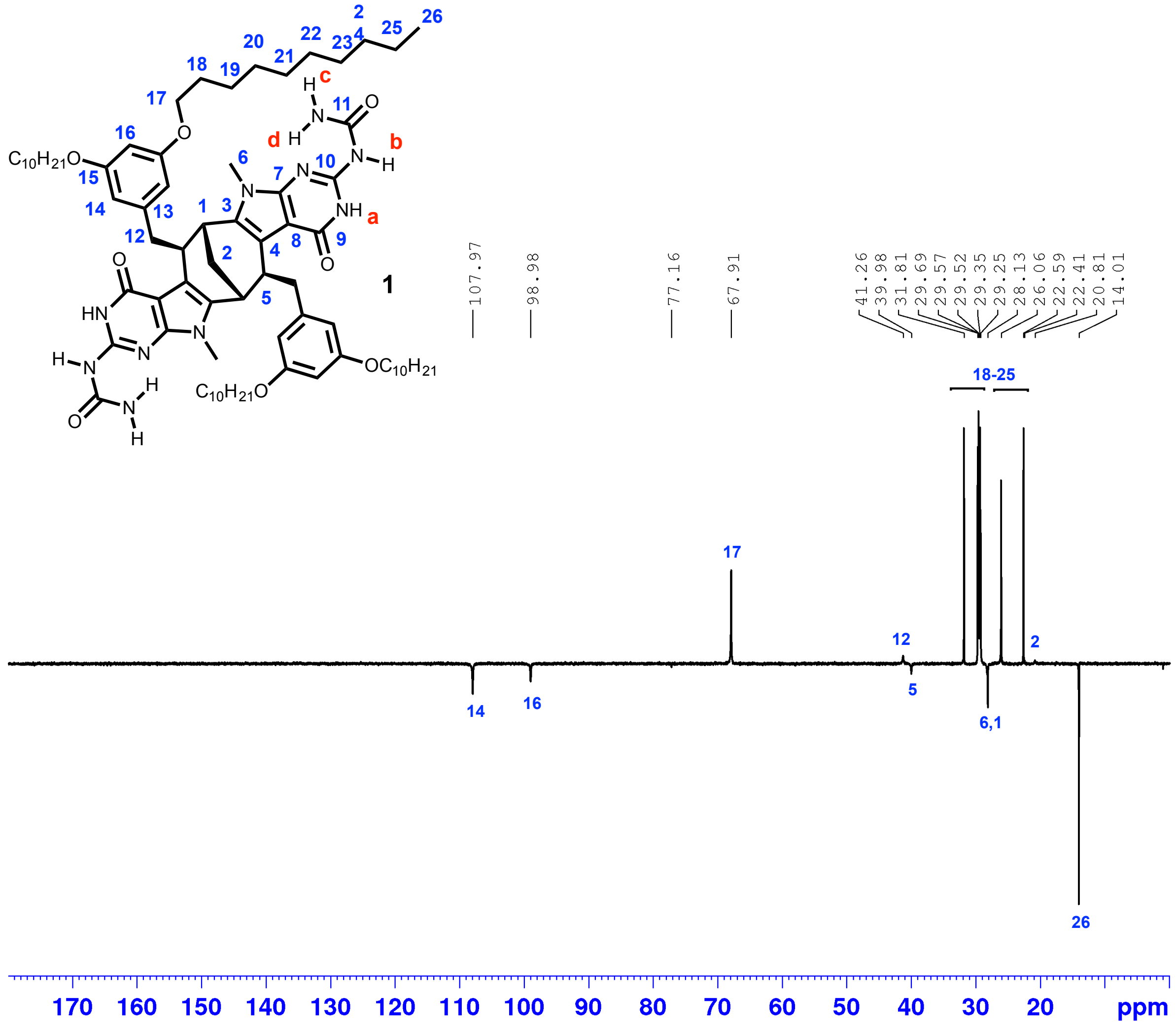

Supplementary Figure 27. DEPT spectrum of **1** in $CDCl_3$.

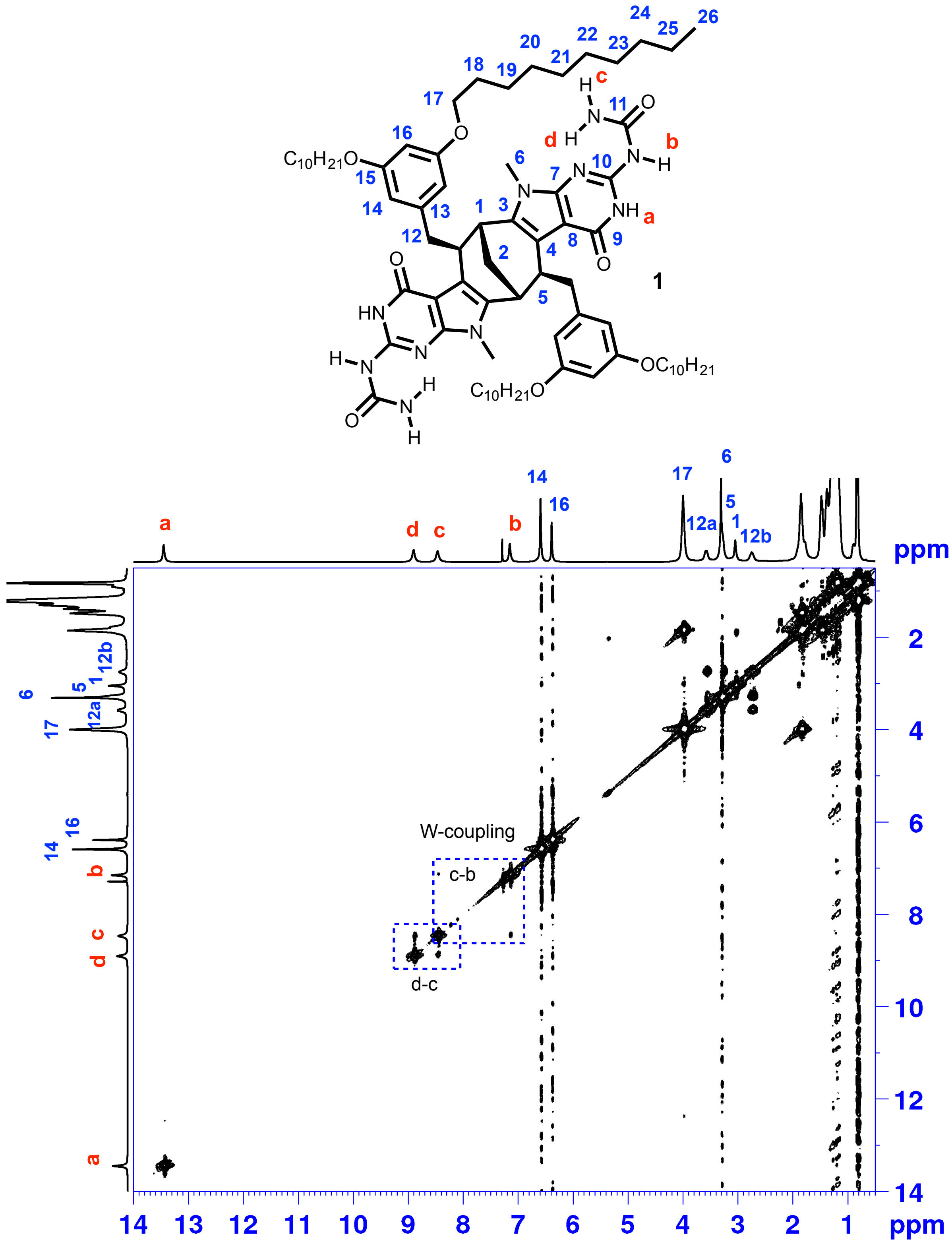

Supplementary Figure 28. COSY spectrum of **1** in $CDCl_3$.

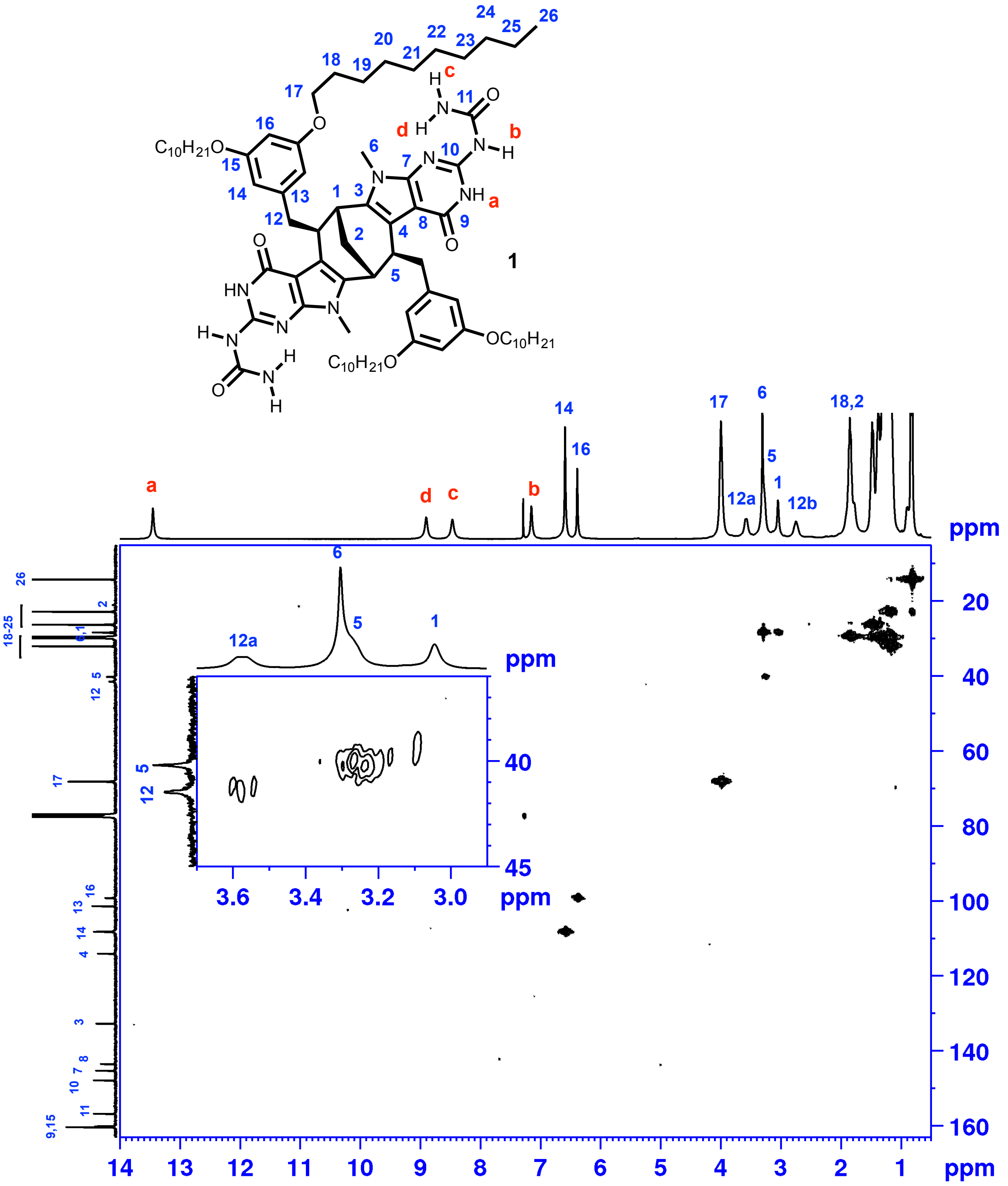

Supplementary Figure 29. ^{1}H-^{13}C HMQC spectrum of **1** in $CDCl_3$.

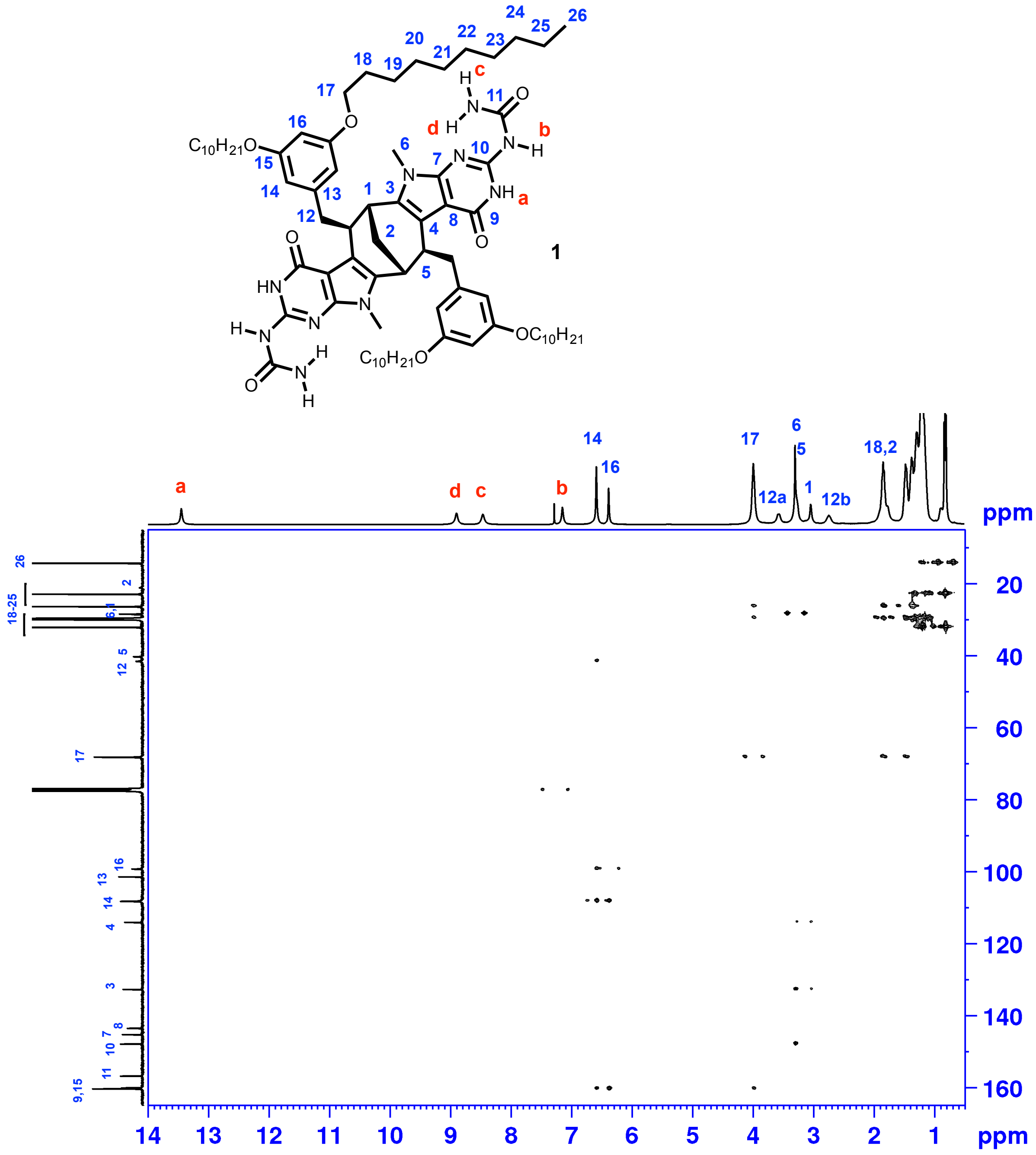

Supplementary Figure 30. HMBC spectrum of 1 in $CDCl_3$.

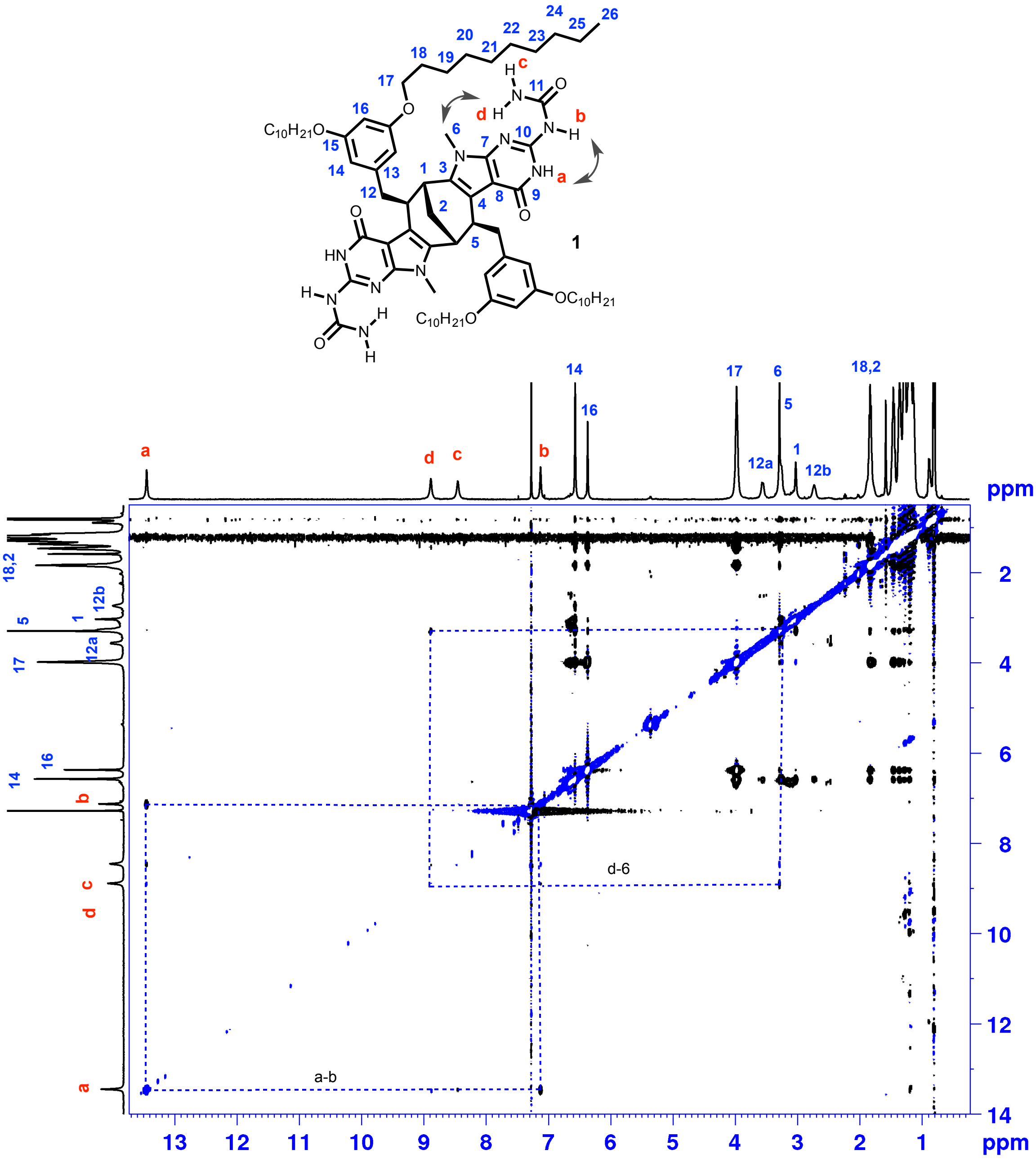

Supplementary Figure 31. ROESY spectrum of **1** in $CDCl_3$.

a)

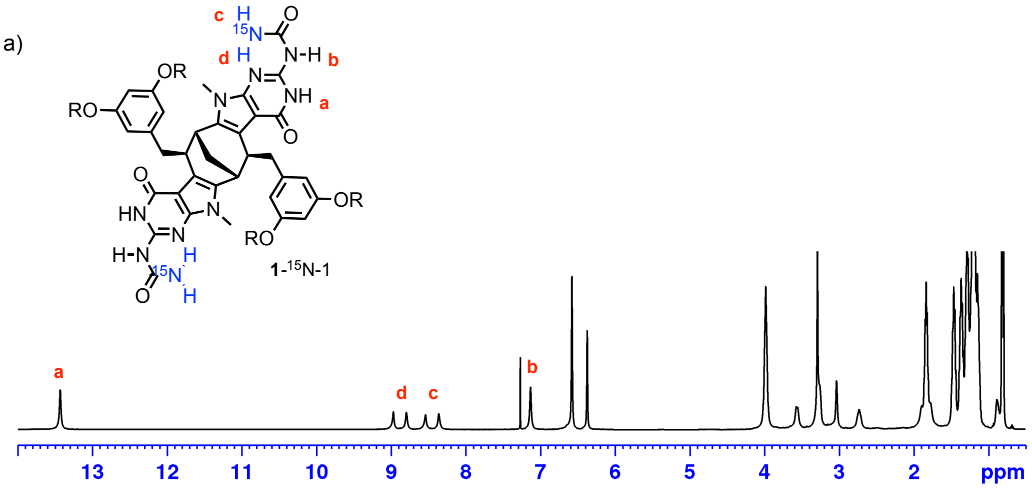

b)

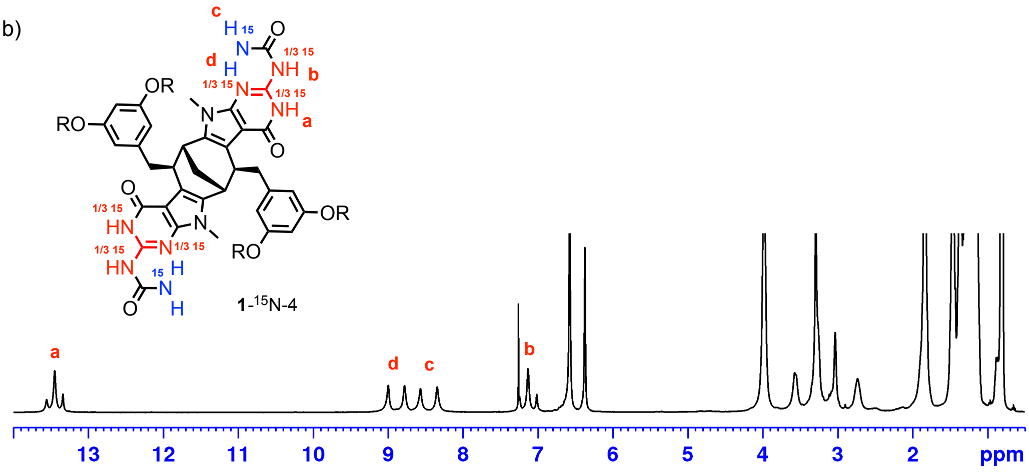

Supplementary Figure 32. (a) ^{1}H NMR spectrum of **1**-^{15}N-1 and (b) **1**-^{15}N-4 in $CDCl_3$. The triplet-like appearance of the resonances of protons a and b in **1**-^{15}N-4 is a result of an overlap of doublet (^{15}N-H) and singlet (^{14}N-H).

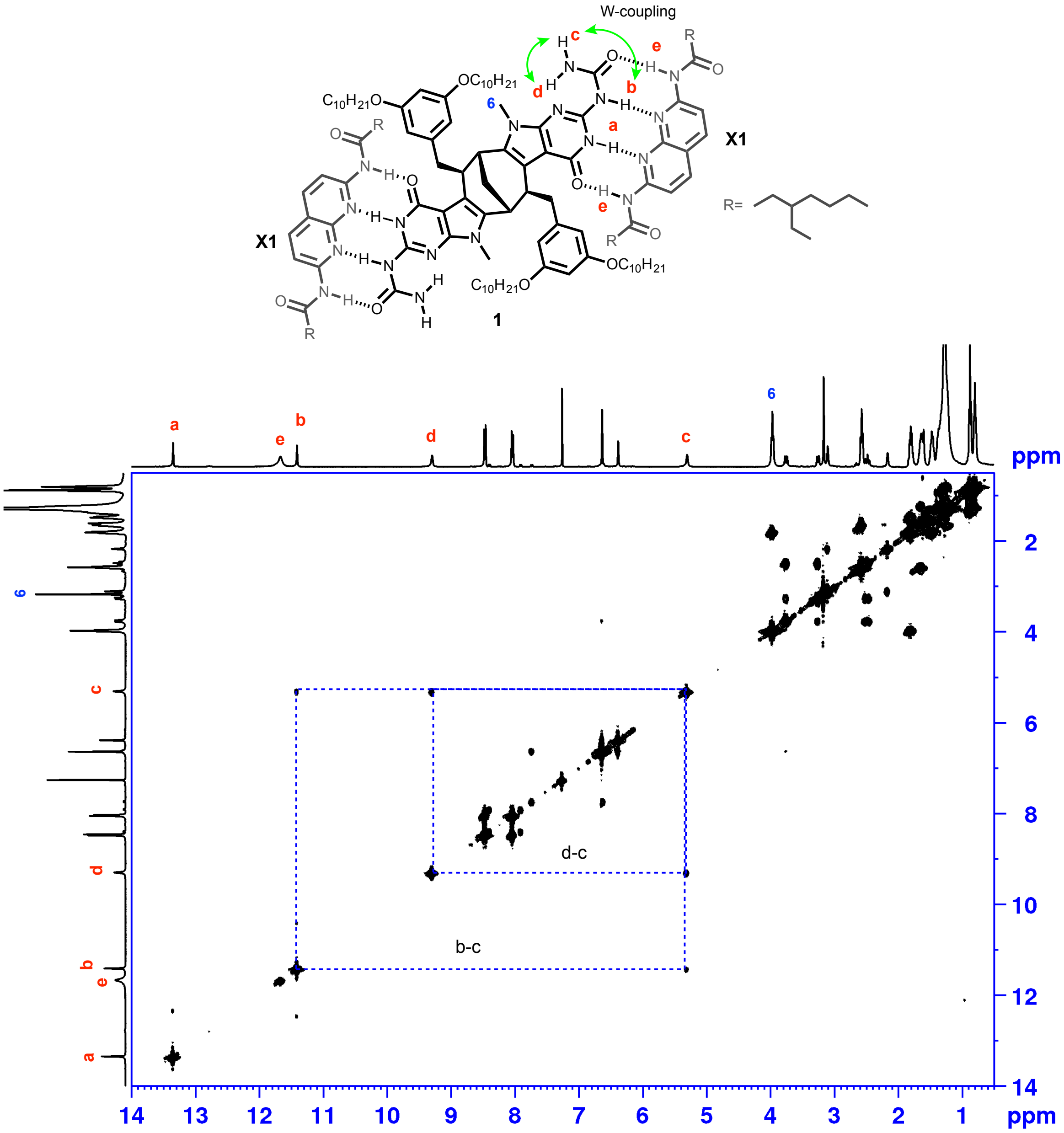

Supplementary Figure 33. COSY spectrum of **1-DAN (X1)** in $CDCl_3$.In order to additionally confirm that all previous NMR assignments made for **1** in $CDCl_3$ corresponds to a proposed conformer and tautomeric form of the isocytosine unit in **1**, we prepared H-bonded 1:2 heterocomplex of **1** with 2,7-diamido-1,8-naphthyridine derivative (DAN). It is well established that DAN forms 4H-bonded heterocomplex with ureidopyrimidinone derivatives using ADDA-DAAD H-bonding mode with remarkable fidelity[6]. The same correlations as in $\mathbf{1}_4$ aggregate where found within complexed PUPY unit, corroborating the assignment of the conformer of **1**.

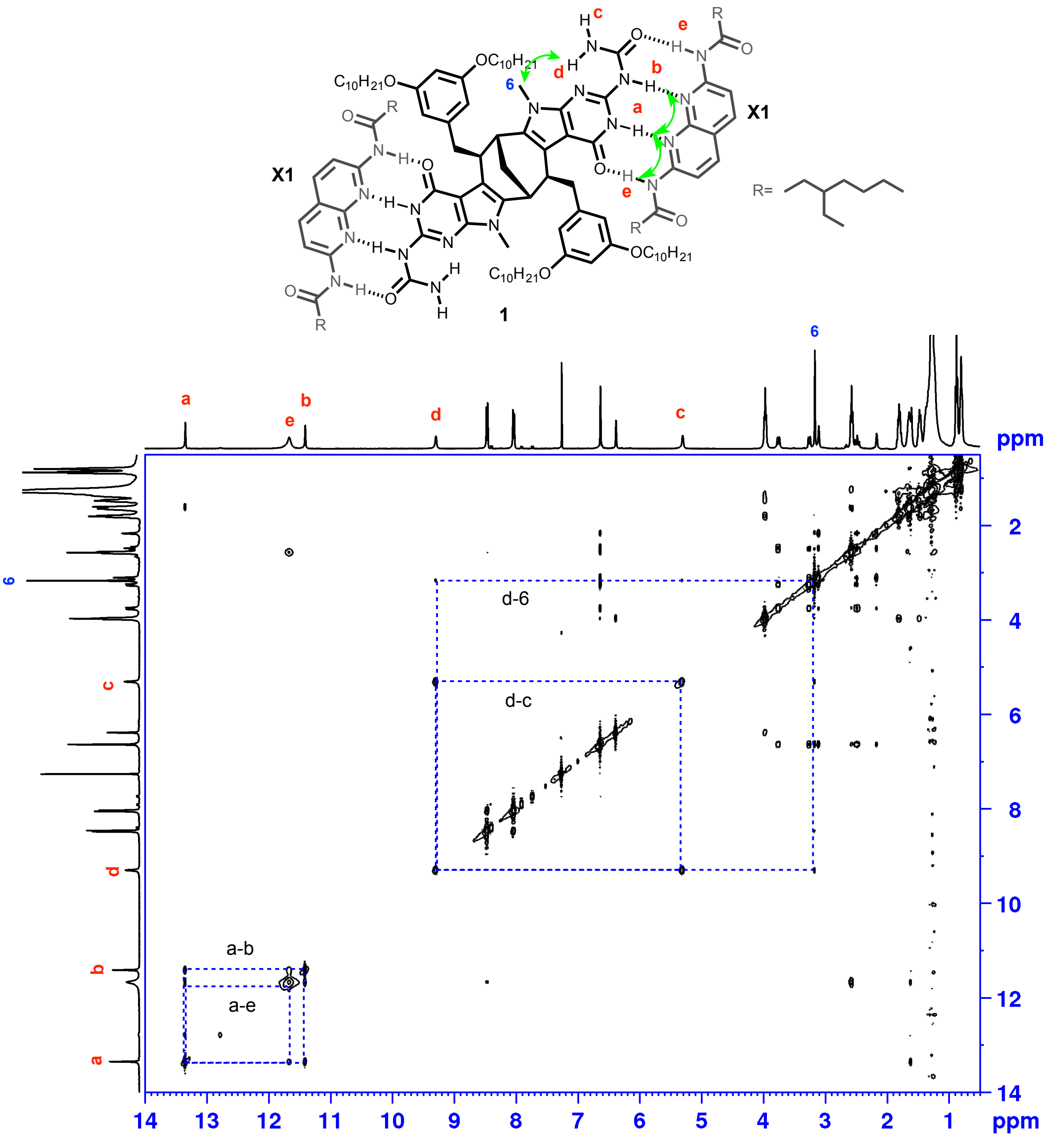

Supplementary Figure 34. NOESY spectrum of **1-DAN (X1)** in $CDCl_3$.

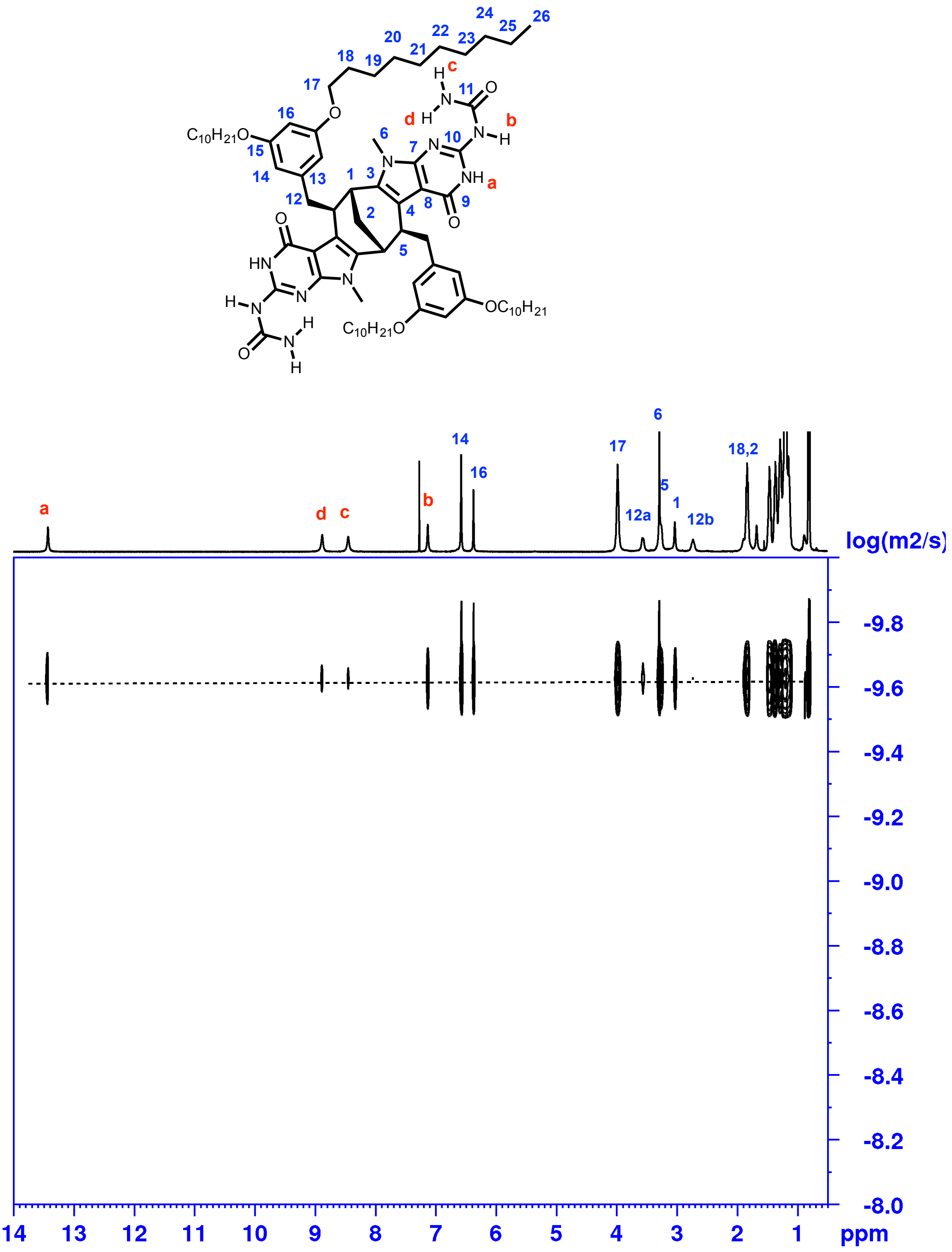

Supplementary Figure 35. DOSY spectrum of **1** in $CDCl_3$. The spectrum indicates the presence of a single aggregate and the value of diffusion coefficient obtained D= $2.39 \cdot 10^{-10}$ m^2s^{-1} agrees well with proposed cyclic tetramer as compared with related systems [5].

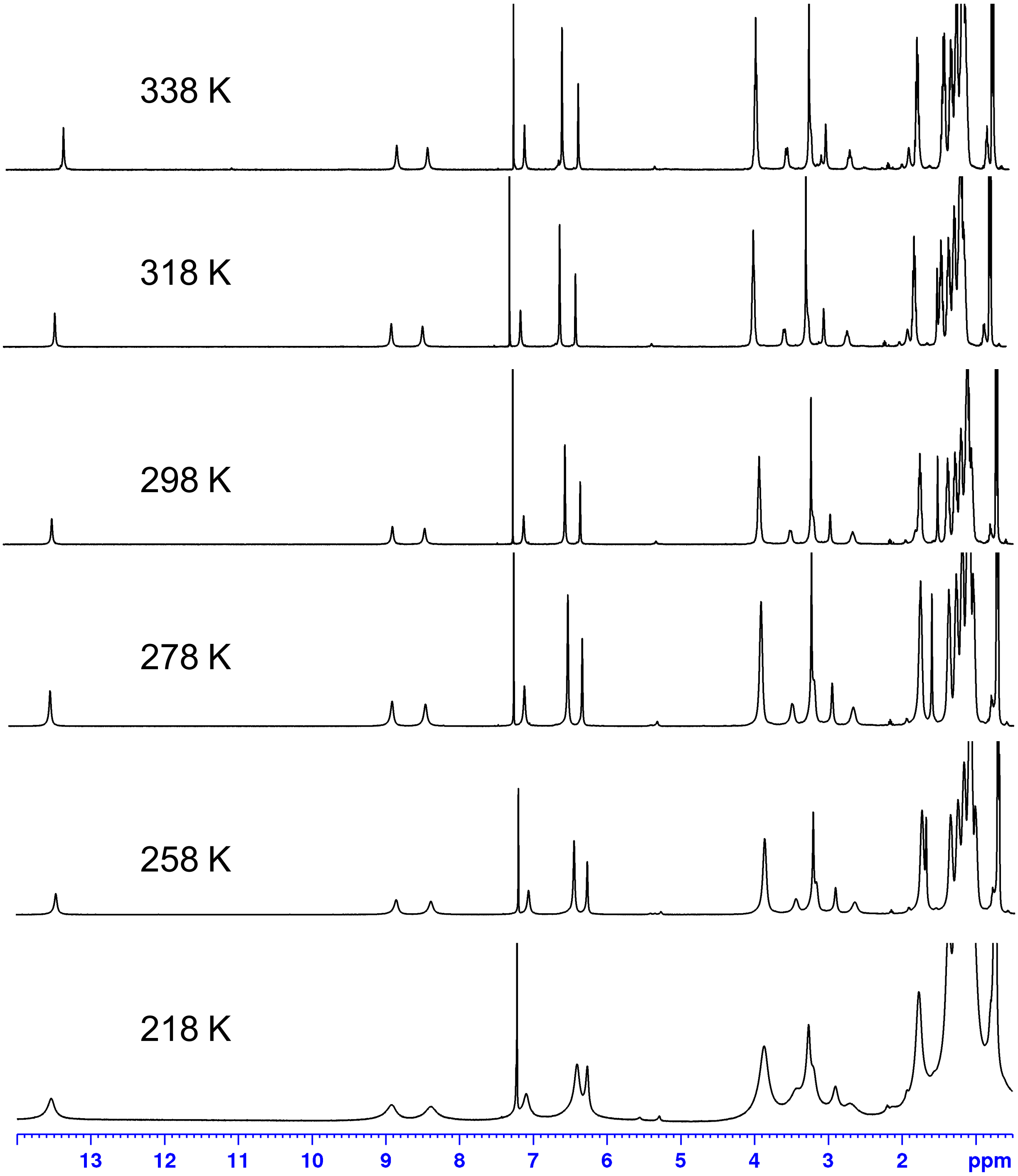

Supplementary Figure 36. Variable temperature ^{1}H NMR of **1**$_4$ in $CDCl_3$. No shift of resonances was observed over the temperature range of 120K.

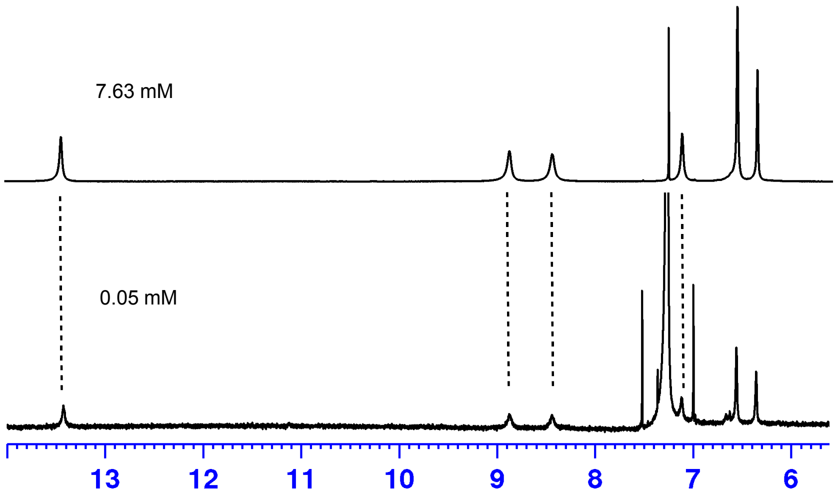

Supplementary Figure 37. Dilution ^{1}H NMR of **1** in $CDCl_3$. No shift of resonances was observed upon sample dilution in $CDCl_3$.

Vapour pressure osmometry (VPO). VPO measurements were done on a KNAUER-7000 instrument equipped with EuroOsmo® v.1.5 software. The chamber temperature was set to 37°C and the head temperature to 38°C. The measurement time was from 2.5 min to 3.5 min. The baseline was obtained by adding drops of pure solvent on both thermistors and auto-zeroed after 30 min. All samples were prepared gravimetrically. Chloroform was washed several times with water to remove ethanol, dried with anhydrous $CaCl_2$ and distilled from powdered 4 Å molecular sieves. Benzil (M_w=210.23) was used as standard. Solutions of **1** in chloroform were used. For each solution at least 3 reproducible measurements were obtained. The slopes of the linear fits of machine response vs concentration for **1** and benzyl solution, respectively, were compared. The ratio 4.8 was obtained corresponding to the formation of cyclic tetramer. Higher than theoretical value might be explained by the limited precision of the instrument or more likely, by further aggregation of cyclic aggregates due to very high concentrations used (up to 90 mM). The formation of a cyclic pentamer is hardly possible due to geometry constrains.

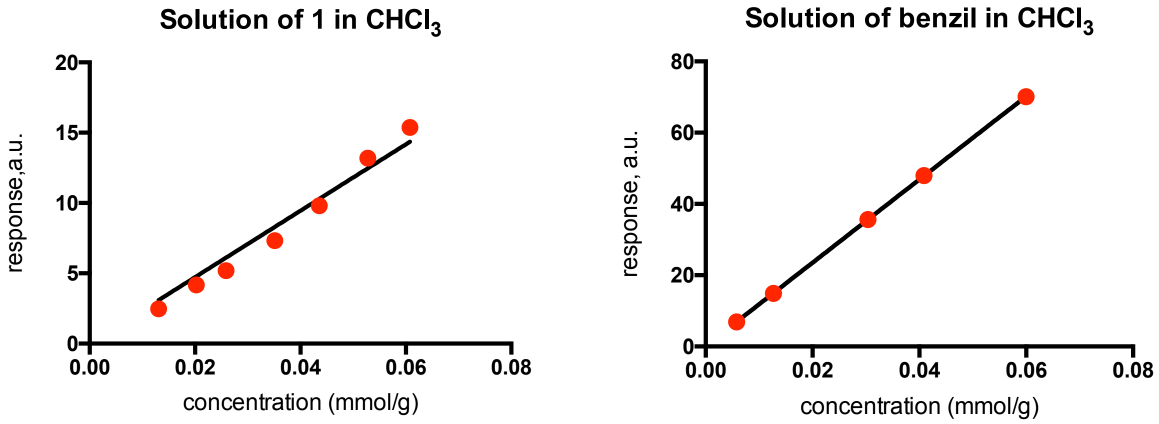

Supplementary Figure 38. VPO response vs concentration for 1 (left) and non-aggregating standard (right).

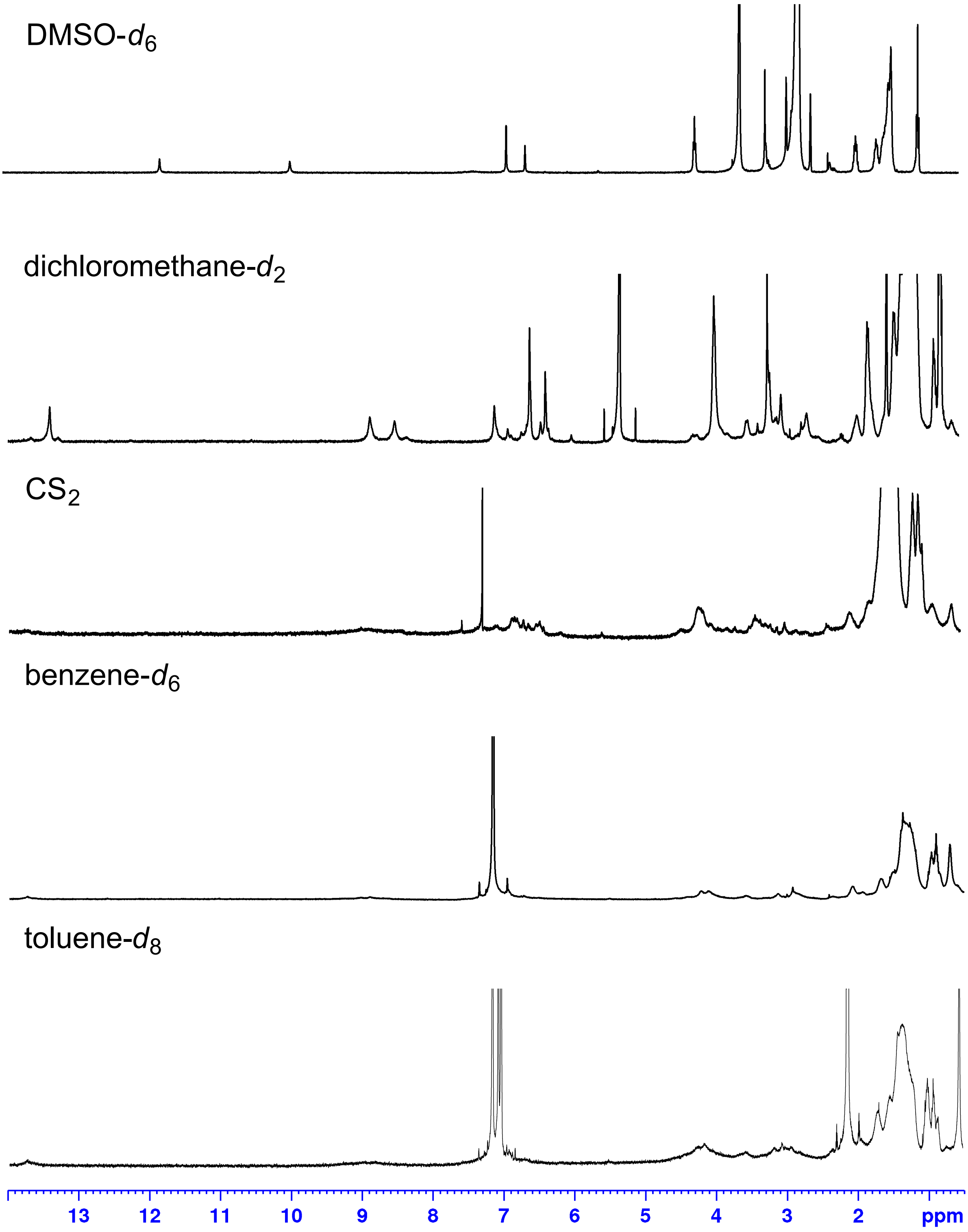

Supplementary Figure 39. ^1H NMR spectra of **1** in solvents of different polarity. Formation of polymeric aggregates was observed in CS_2 and aromatic solvents.

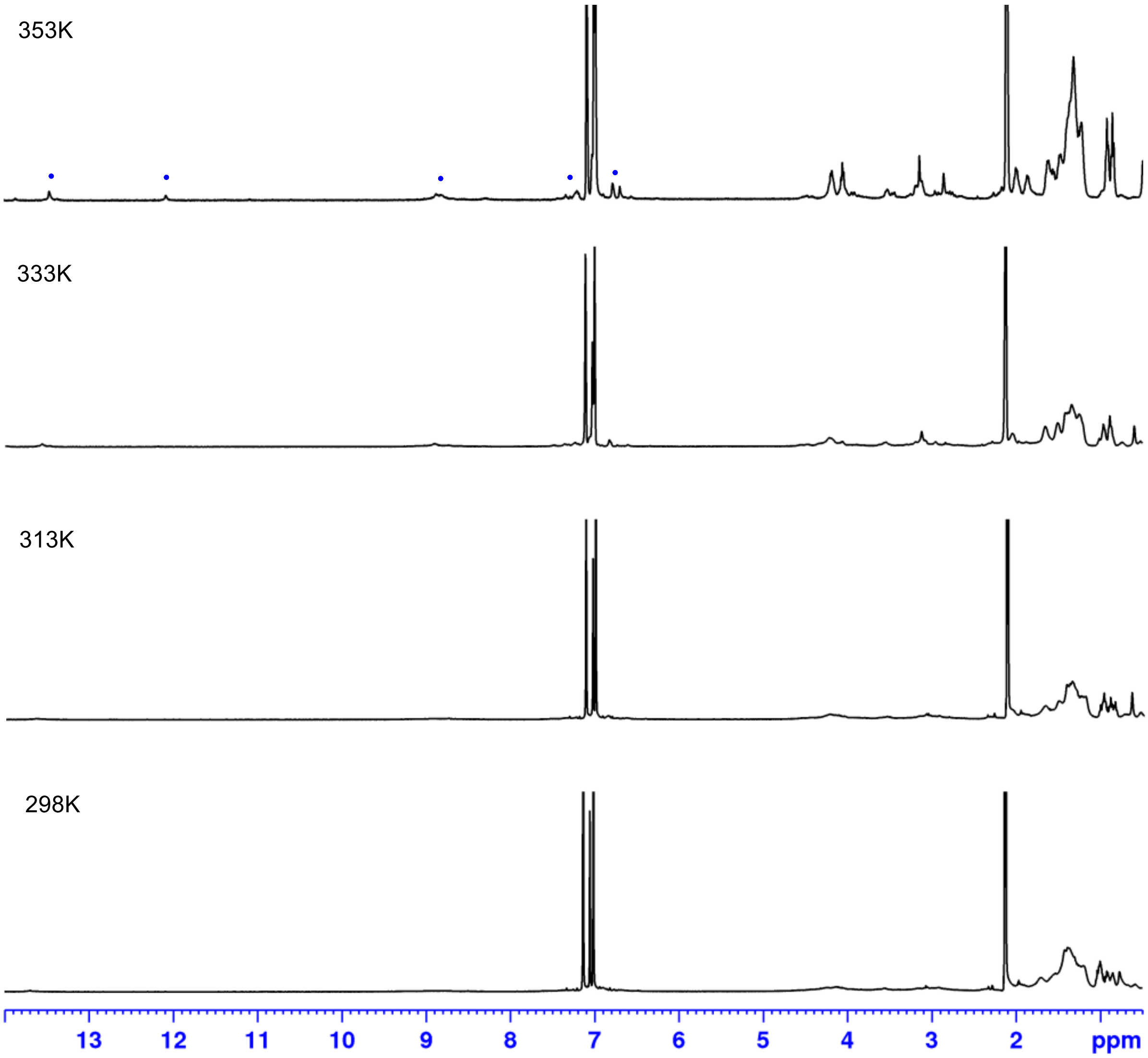

Supplementary Figure 40. Variable temperature ^{1}H NMR of $\mathbf{1}_n$ in toluene-d_8. Notable dissociation of polymer into tetrameric units occurs only at 353 K (blue circles).

Dynamic Light Scattering. These experiments were carried out on a Zetasizer Nano Z (Malvern) instrument at 298K. The sample of 1_n in toluene was prepared by dissolving 15.0 mg of **1** in 3 ml of toluene and aging for 2 days. The sample of C_{70}@$\mathbf{1}_n$ was prepared by dissolving 7.0 mg of **1** and 1.1 mg of C_{70} (1:C_{70}-4:1) in 3 ml $CDCl_3$ and aging for 2 days. All samples were filtered through PTFE membrane filter (AcroDisc, 0.12 micron) before measurements.

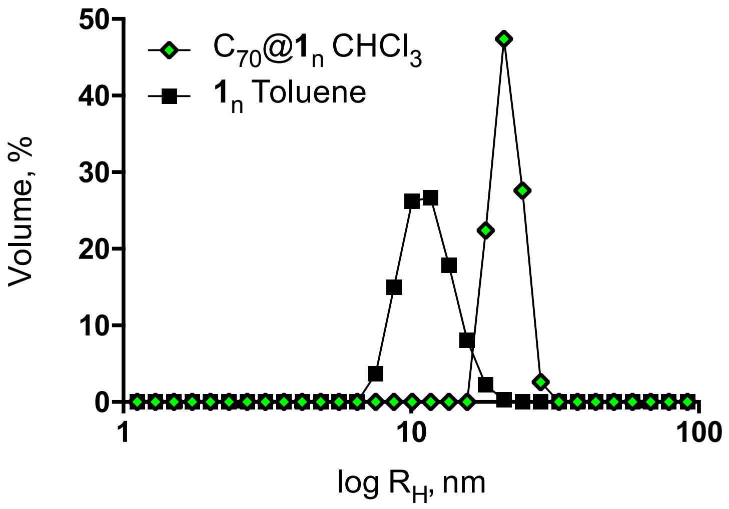

Supplementary Figure 41. Dynamic light scattering data for a solution of **1** in toluene (black squares) and C_{70}/**1** mixture in $CDCl_3$ (green squares).

UV-vis spectroscopy. UV-vis spectra were recorded at 298K on Perkin Elmer Lambda 35 spectrometer equipped with Peltier temperature control module. The stock solution of C_{70} in $CDCl_3$ was prepared by dissolving 0.70 mg of C_{70} in 2 ml of $CDCl_3$. The sample of C_{70}@$\mathbf{1}_n$ was prepared by dissolving of 2.10 mg of **1** in 1.0 ml of C_{70} stock solution. The first measurement was done after 1 h and repeated after 3h, 24 h and 48 h. (1 mm cell, 700-360 nm range, 1 nm slit, 60 nm/min, UV-vis lamp switch at 360 nm). No further changes were observed after 24 h.

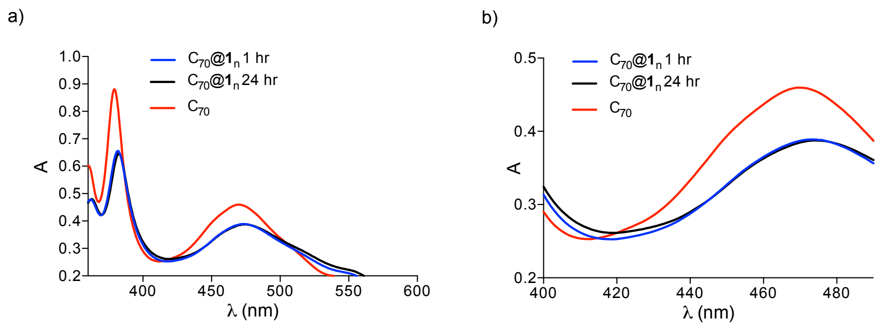

Supplementary Figure 42. Time-dependent UV-vis spectrum of C_{70}/**1** mixture in $CDCl_3$.

Gel permeation chromatography (GPC). GPC analysis was performed on Agilent 1260 Infinity GPC/SEC system. (Mobile phase: toluene; Column temperature: 25°C; Flow rate: 1 ml/min; Injection volume: 20 μl; Detector: RID). The column was calibrated against the set of polystyrene standards (4-100 kDa) (Supplementary Fig. 43).

Please note, the calibration curve obtained was used for the calculation of M_w and polydispersity index (PDI). The hydrodynamic properties of polystyrene chain and mass density along the chain is very different from the hollow supramolecular polymer $\mathbf{1}_n$. Therefore, the molecular weight calculated in this way is a very rough estimate and can be used for qualitative discussion only.

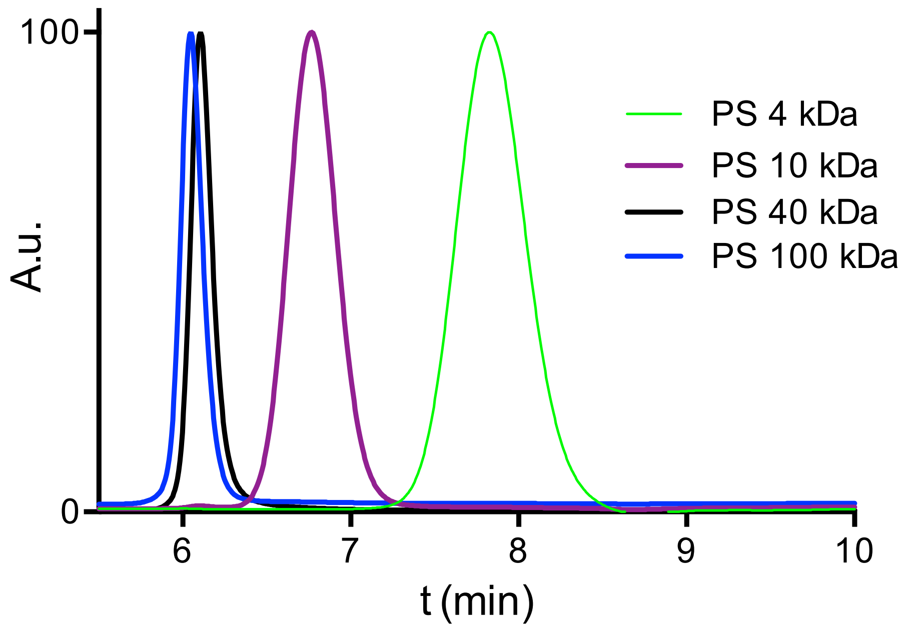

Supplementary Figure 43. Normalized GPC traces for polystyrene standards.

The sample of compounds **1**, **3** and **4** were prepared by dissolving 2.5 mg of corresponding compound in 1 ml of toluene. The sample of **1** was analysed immediately after the preparation and then after 24 hr. The fresh sample showed bimodal distribution in GPC trace, which was attributed to a mixture of tetrameric and short olygomeric assemblies (Supplementary Fig. 44). After aging, the low molecular mass peak disappeared completely resulting in chromatogram displaying a single high molecular mass peak of PDI=1.26 (M_w=45990 Da, M_n=36213 Da) This peak, corresponding to polymer $\mathbf{1}_n$, shows slight tailing

caused by dissociation of the polymeric aggregate during passage through the column. These findings were in agreement with ^{1}H NMR spectroscopy data where at least 24 hr. aging was required to achieve full polymerization. The low molecular mass component of the bimoidal peak was nearly identical in hydrodynamic radius to control compound **3**, which could only form tetrameric assemblies, thus corroborating slow aggregation of tetrameric units of $\mathbf{1}_4$ into polymer $\mathbf{1}_n$ (Supplementary Fig. 44).

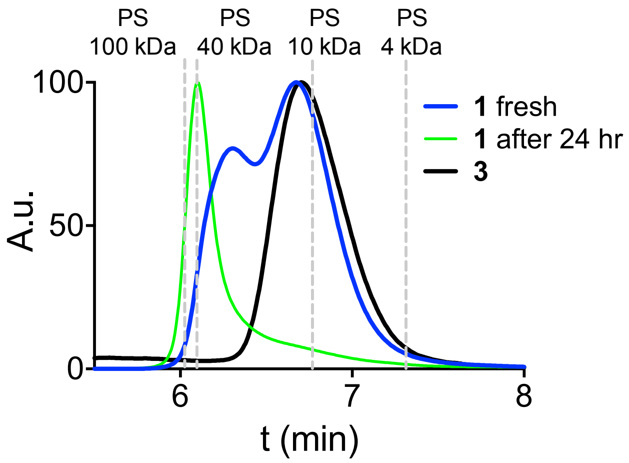

Supplementary Figure 44. Normalized GPC traces of **1** and **3** in toluene. The vertical dashed lines indicate the elution time of polystyrene standards (see Supplementary Fig. 43)

The control compounds **3** and **4** gave well-defined peaks by GPC (Supplementary Fig. 45). According to retention time, compound **3** forms stable tetrameric assemblies (PDI = 1.13) that are not destroyed during analysis. In contrast, a well-defined peak (PDI = 1.10) in the trace of **4** corresponds to fully monomeric compound.

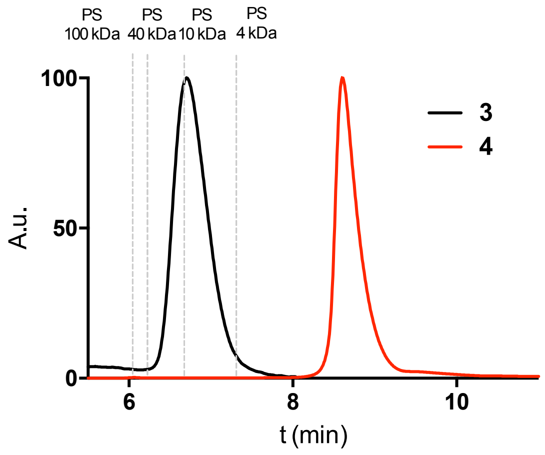

Supplementary Figure 45. Normalized GPC traces of **3** and **4** in toluene. The vertical dashed lines indicate the elution time of polystyrene standards.

Lower stability of the cyclic aggregates of compound **4** is most likely attributed to repulsive dipole-dipole interaction in H-bonded aggregate (Supplementary Fig. 46). NMR spectroscopy data confirmed that compound **4** indeed exist as the conformer shown below.

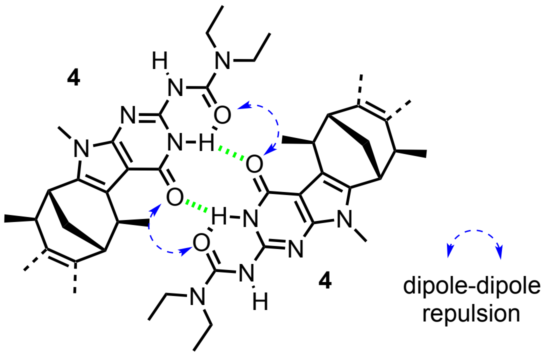

Supplementary Figure 46. Repulsive secondary interactions between adjacent carbonyl groups in 2H-bonded aggregate of **4**.

In order to get additional proof that compound **1** exists as a H-bonded tetramer in chloroform solution, we performed GPC analysis of **1** in chloroform using a set of functionalized cyclodextrins as calibration standards (Viscotek DM-400 SEC system equipped with three Shodex columns (KF-805, KF-804 and KF-802.5) connected in series; Mobile phase: chloroform; Column temperature: 25°C; Flow rate: 1 ml/min; Injection volume: 20 μl; Detector: RID) (Supplementary Fig. 47). These cyclic standards appended with alkyl chains were expected to mimic the hydrodynamic properties of $\mathbf{1}_4$ more closely as compared to linear polystyrene[7]. Polydispersity of the peak (PDI=1.09) indicated the presence of a well defined supramolecular aggregate with a molecular mass M_w =5666 Da, in good agreement with the one of tetrameric aggregate (M_w(calcd)= 5136 Da) (Supplementary Fig. 48).

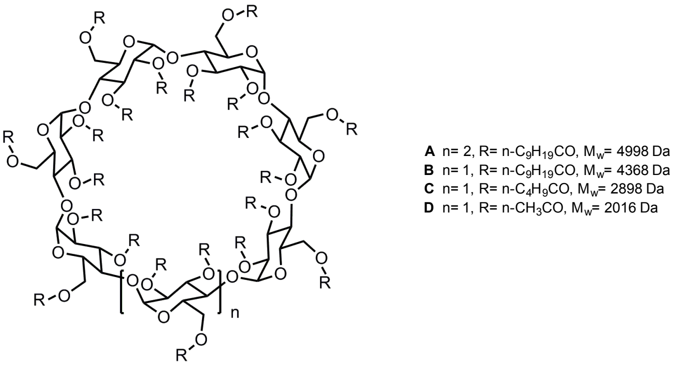

Supplementary Figure 47. Cyclodextrin standards **A-D** used for gel permeation chromatography.

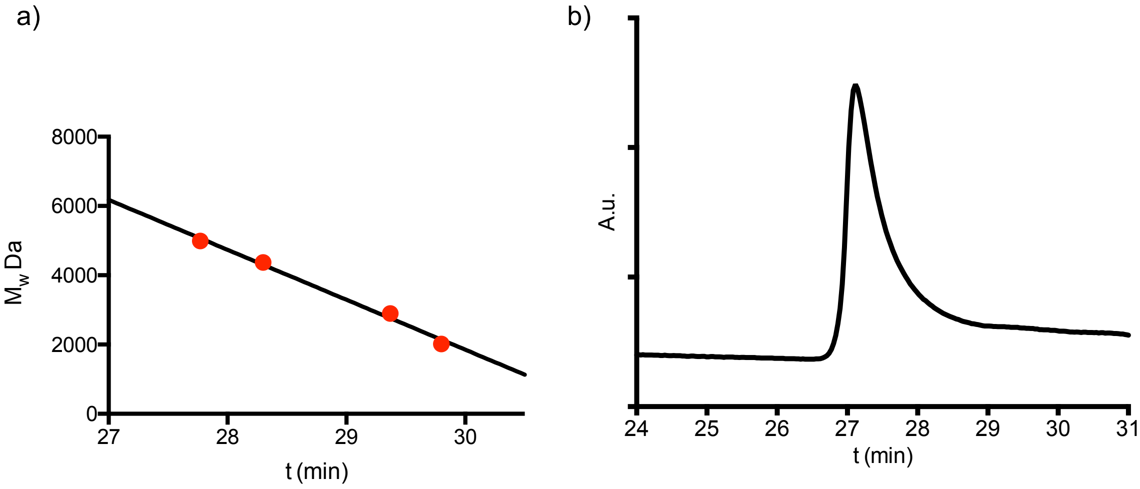

Supplementary Figure 48. (a) calibration curve obtained with standards **A-D** (Supplementary Fig. 47) and (b) gel permeation chromatogram of **1** in $CHCl_3$.

Atomic Force Microscopy (AFM). AFM imaging was carried out in ambient conditions using the JPK NanoWizard 3. Monomer **1** was investigated in intermittent contact mode (AC mode) using the MPP-12220 probe (Veeco). Polished silicon surface which was ultrasonicated in ethanol and cleaned in ammonia-hydrogen peroxide solution was used as a substrate. The sample was prepared by drop-casting the solution (x88 dilution of stock solution) on the substrate, and drying in nitrogen stream after 30 s.

Monomer **2** was investigated in Quantitative Imaging mode using PPP-XYNCSTR probe (Nanosensors). The sample was prepared by drop-casting the solution on the freshly cleaved mica and drying with nitrogen stream after 30 s.

IR spectroscopic studies. Infrared spectra were recorded in transmission geometry on an ALPHA FTIR spectrometer (Bruker Optik GmbH, Germany), equipped with a room temperature detector DLATGS. The spectral resolution was set at 2 cm^{-1}. Spectra were acquired from 100 interferogram scans. Samples were dissolved in chloroform-D ($CDCl_3$) at 0.005–0.02 M concentration. Spectra were recorded in transmission cell with CaF_2 windows and 55 µm path length. Parameters of the bands were determined by fitting the experimental spectra with Gaussian-Lorentzian shape components using GRAMS/A1 8.0 (Thermo Scientific) software.

Please note, Supplementary Figure 49 shows infrared absorption spectra in the fingerprint (1350–1780 cm^{-1}) and N–H group stretching (3030–3550 cm^{-1}) spectral regions for **1** and **1**-^{15}N-1. Vibrational modes coupled with vibrations of urea NH_2 group can be recognized by frequency downshift for ^{15}N-labeled sample (Supplementary Table 1). Thus, the intense band near 1703 cm^{-1} of compound **1** in $CDCl_3$ downshifts to 1699 cm^{-1} in the case of isotope-labeled compound. Similar band (1696 cm^{-1}) observed in infrared spectrum of urea in $CHCl_3$ was assigned to C=O stretching vibration, ν(C=O)[8]. Downshift of this band due to ^{15}N-isotope labeling indicates considerable coupling of C=O stretching and NH_2 deformation vibrational modes.[9] The bands at 1635 and 1421 cm^{-1} also exhibit red shift upon ^{14}N/^{15}N exchange and was assigned to NH_2 deformation, δ(NH_2), and C–N antisymmetric stretching, ν_a(C–N), vibrations, respectively.[9-11] In the high frequency spectral region the band at 3353 cm^{-1} downshifts to 3346 cm^{-1} for ^{15}N-labeled compound **1** and was assigned to N–H stretching vibration, ν(N–H), of terminal urea NH_2 group. An additional support for provided assignments comes from the analysis of infrared spectrum of the synthetic intermediate **8a** which does not

possess the urea group (Supplementary Fig. 50); the characteristic urea group bands at 1703 and 1421 cm^{-1} disappear from the spectrum. Addition of C_{70} into the $CDCl_3$ solution triggers remarkable changes in the infrared spectrum of compound **1**. Thus, ν(C=O) band of urea group downshifts as much as 44 cm^{-1}, while the ν_a(C–N) band upshifts in frequency by 28 cm^{-1} (Supplementary Fig. 49, Supplementary Table 1). In addition, the $^{14}N/^{15}N$-isotope substitution sensitive ν(N–H) band downshifts from 3353 to 3317 cm^{-1}. All these changes are consistent with involvement of both C=O and NH_2 groups of urea linkage in hydrogen bonding interaction; it is well-known that such interaction results in decreased frequencies of ν(C=O) and ν(N–H) modes, while opposite shift is expected for the ν_a(C–N) band[8,9,11-14].

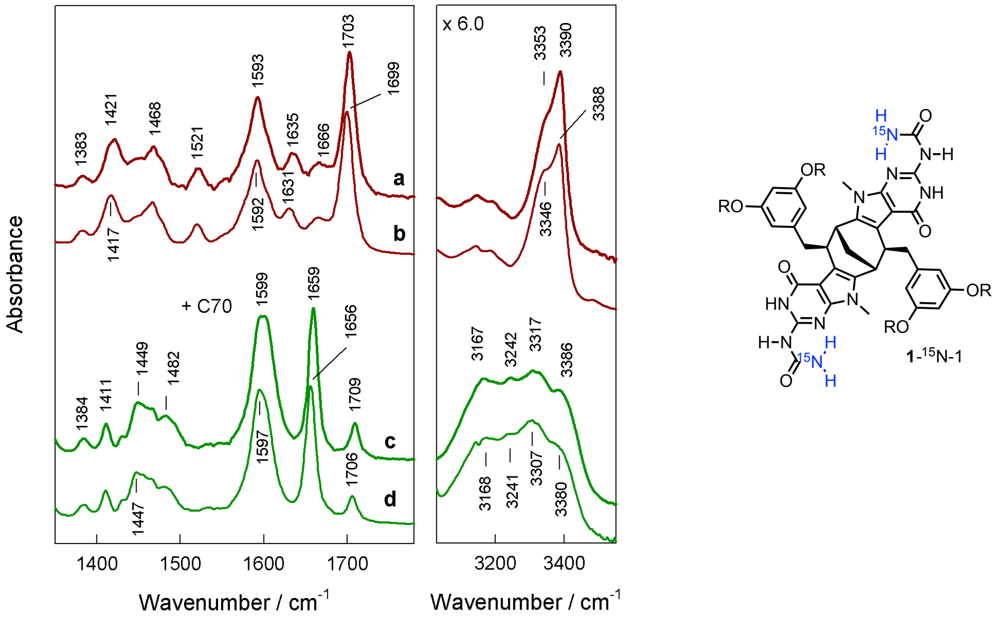

Supplementary Figure 49. Infrared absorption spectra of compound **1** (a) and compound **1**-^{15}N-1 (b) in $CDCl_3$ before introduction of C_{70}. (c) and (d), one hour after introduction of C_{70} into a $CDCl_3$ solution of **1** and **1**-^{15}N-1, respectively.

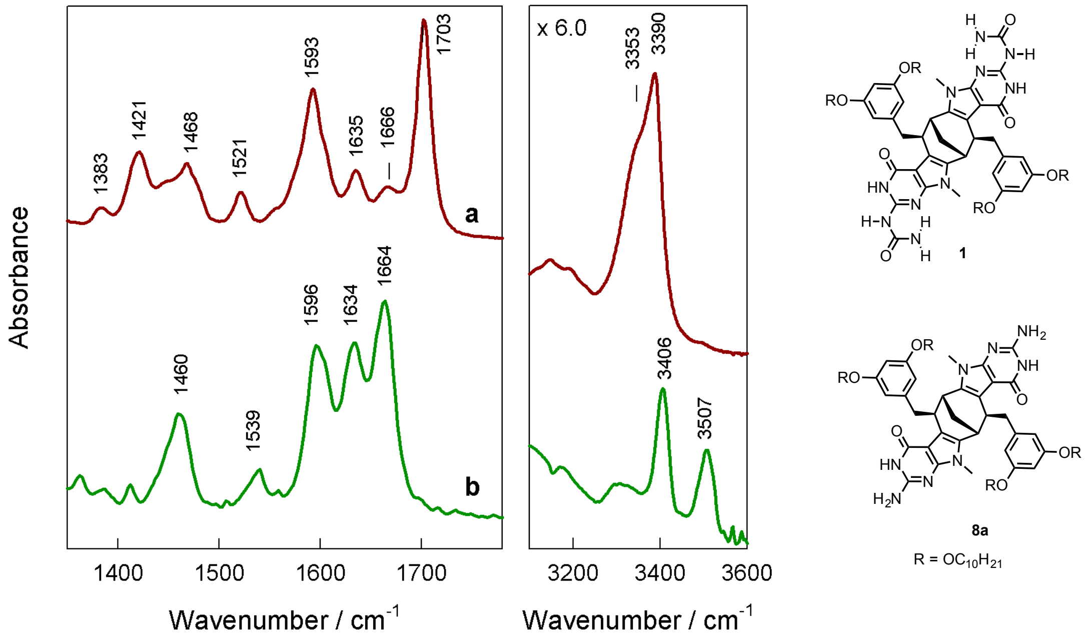

Supplementary Figure 50. Infrared absorption spectra of compound **1** and model compound **8a** in $CDCl_3$. (a) compound **1**; (b) model compound **8a**, which does not possess the urea group. Absence of 1703 and 1421-cm^{-1} bands in model compound spectrum provides evidence for assignment of these bands to C=O and C–N stretching vibrations of urea group of compound **1**.

Supplementary Table 1. Assignments of urea group bands (cm^{-1}) of **1** and **1**-^{15}N-1.

Mode	Compound 1	Compound **1**-^{15}N-1	Compound 1 in the presence of C_{70}	Compound **1**-^{15}N-1 in the presence of C_{70}
ν(N–H)	3353	3346	3386 / 3317	3380 / 3307
ν(C=O)	1703	1699	1659	1656
δ((NH$_2$)	1635	1631	–	–
ν_a(C–N)	1421	1417	1449	1447

Abbreviations: ν, stretching; ν_a, antisymmetric stretching; δ, deformation.

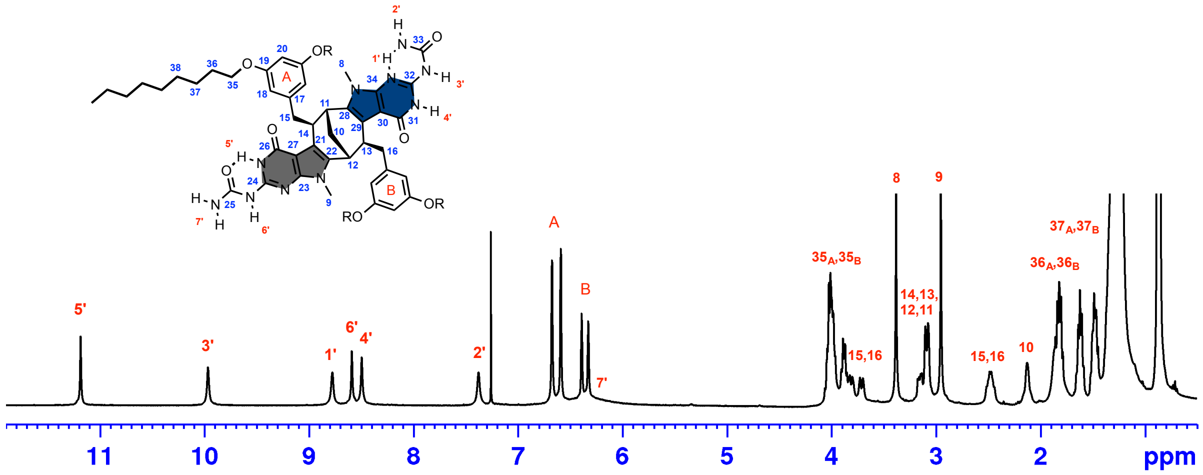

Supplementary Figure 51. ^{1}H NMR spectrum of C_{60}@**1**$_4$ in $CDCl_3$. Assignment of proton resonances were made using COSY, HSQC, HMBC and ROESY spectra (see below).

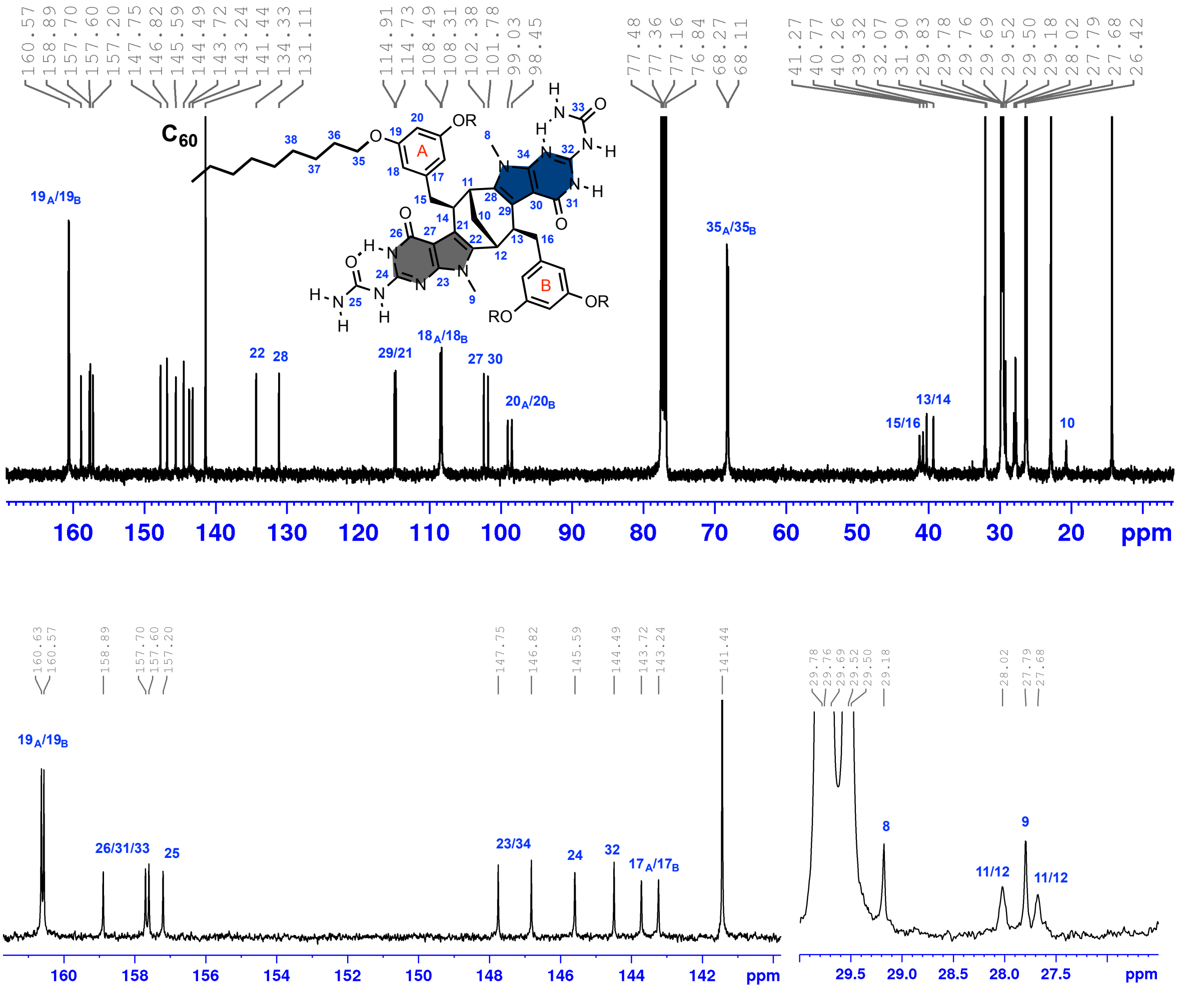

Supplementary Figure 52. ^{13}C NMR spectrum of C_{60}@**1**$_4$ in $CDCl_3$. Assignment of carbon resonances were made using DEPT, HSQC and HMBC spectra (see below).

a) C_{60}@**1**$_4$

b) **1**$_4$

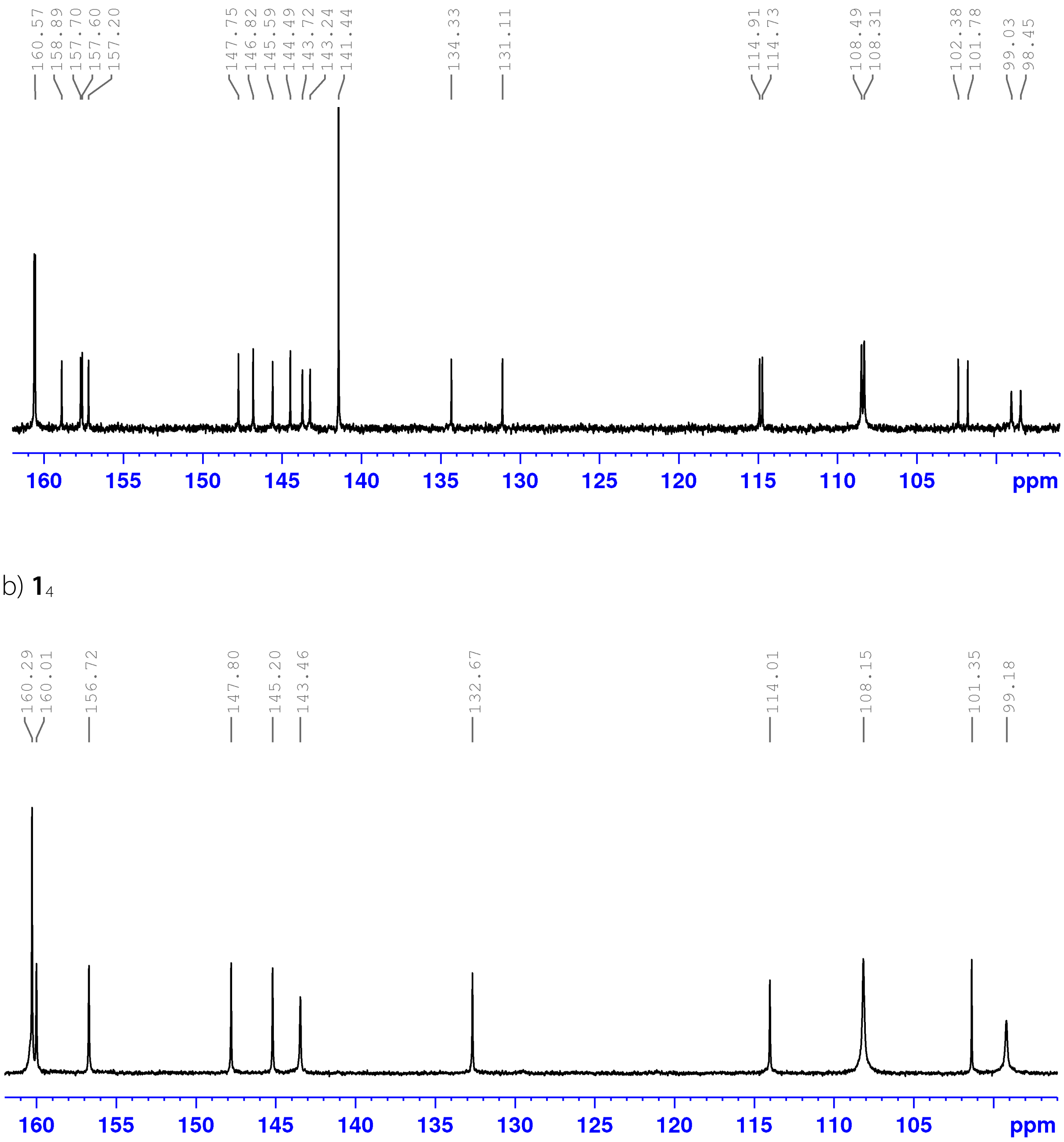

Supplementary Figure 53. Comparison of ^{13}C NMR spectra of **1** and C_{60}@**1**$_4$ in $CDCl_3$. Doubling of all resonances, except for C_{60} was observed upon complexation.

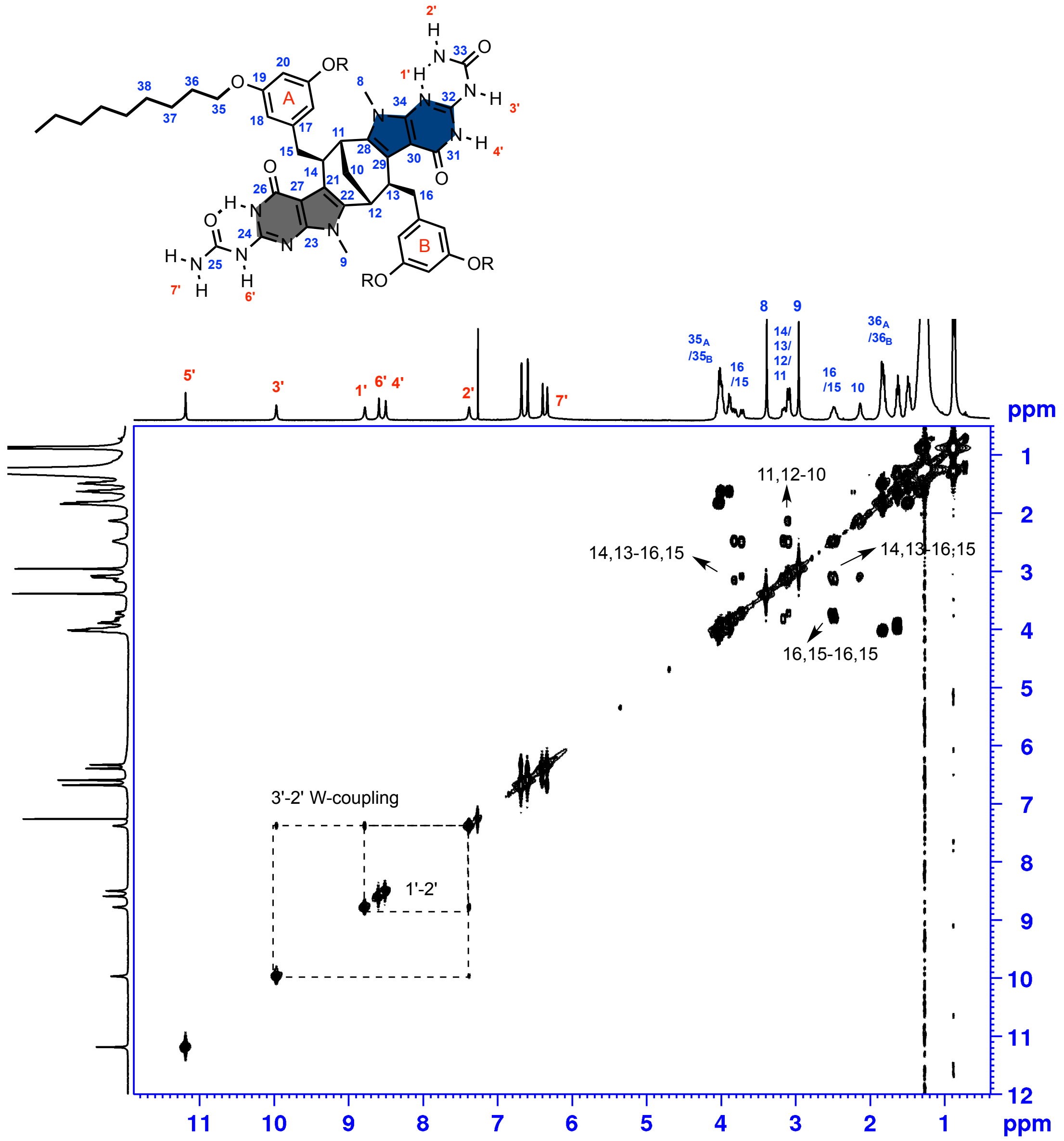

Supplementary Figure 54. COSY spectrum of $C_{60}@\mathbf{1}_4$ in $CDCl_3$.

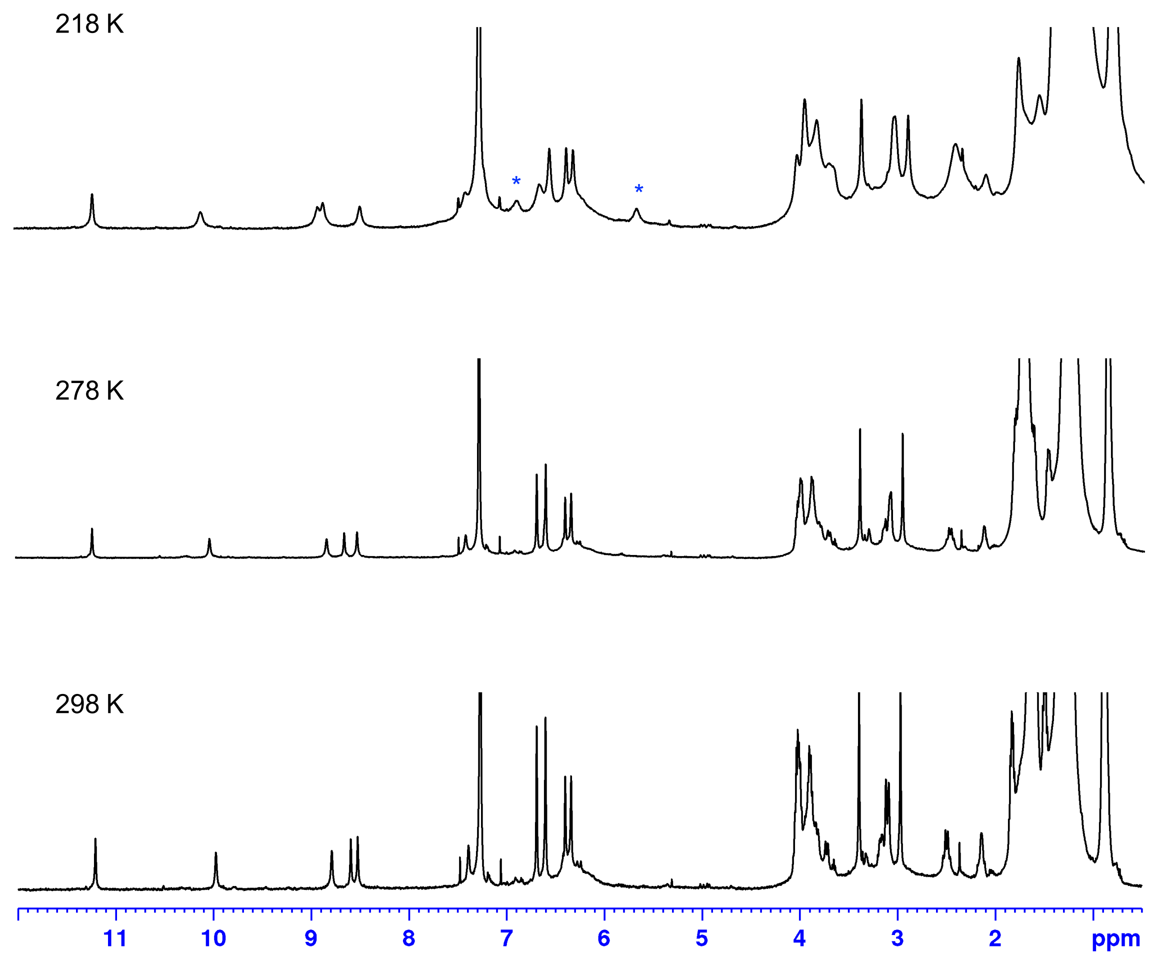

Supplementary Figure 55. Variable temperature ^{1}H NMR spectra of C_{60}@$\mathbf{1}_4$ in $CDCl_3$. The resonances labelled * correspond to protons 7′ which are too broad to observe at higher temperature as a result of the facile rotation around C-N bond.

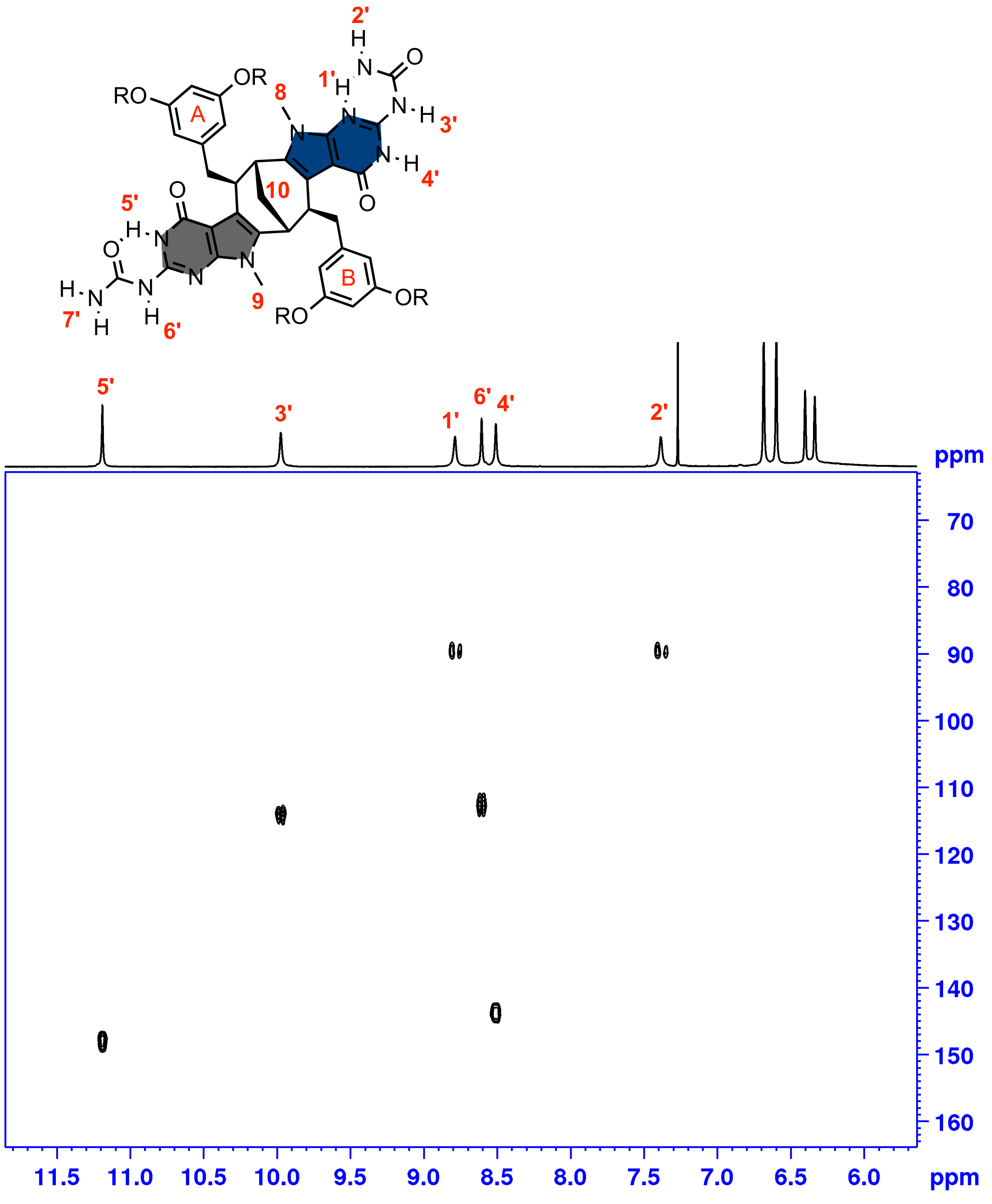

Supplementary Figure 56. ^{15}N-^{1}H HSQC spectrum of C_{60}@**1**$_4$ in $CDCl_3$. The spectrum shows that all downfielded protons are residing on nitrogen atoms and thus, indicates that no enolic tautomeric form of the isocytosine ring is involved in the aggregation. The resonance of the nitrogen atom connected to protons 7′ was not revealed due to the broadness of the signal. The values of the chemical shift of ^{15}N nuclei fully support the assignment of the resonances.

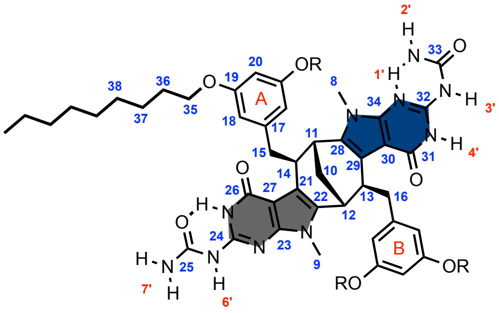

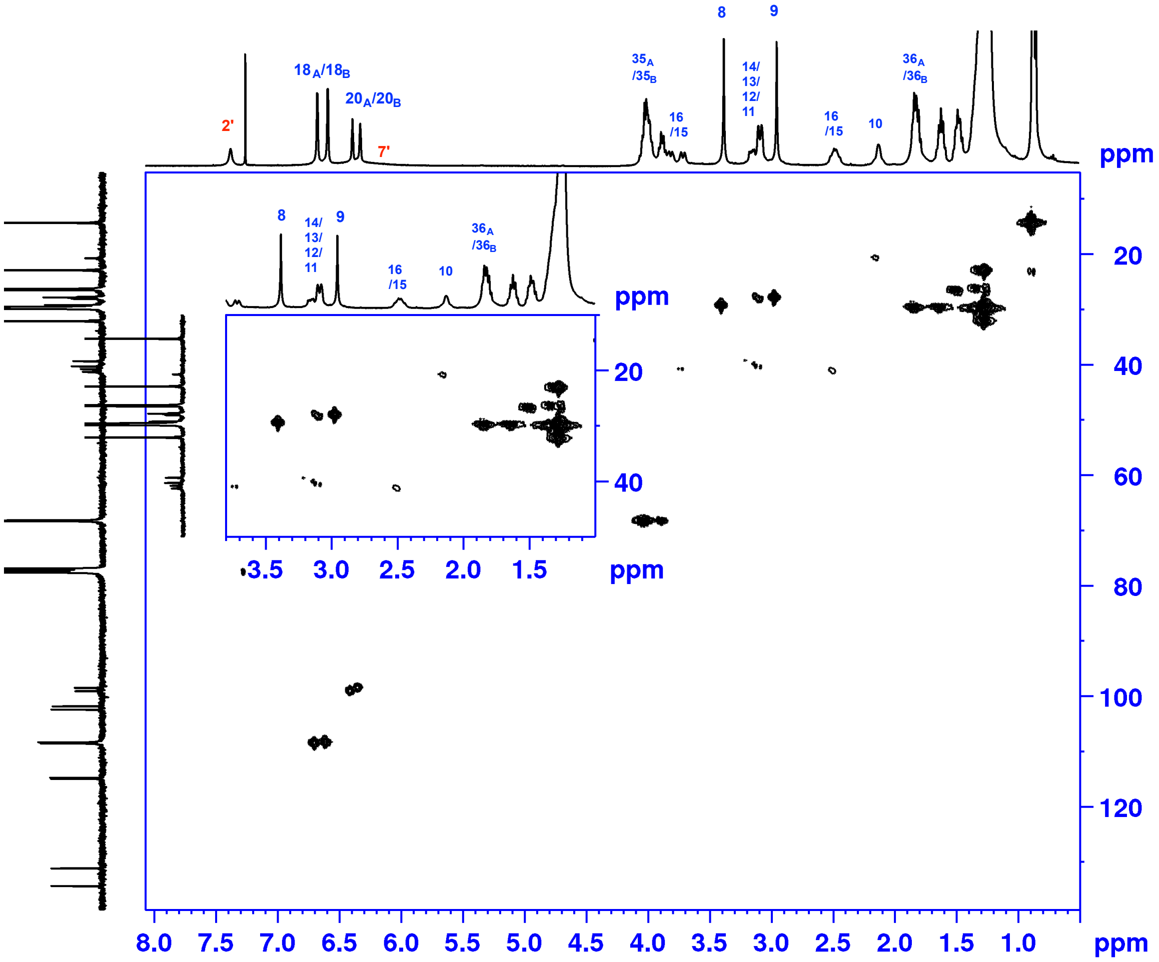

Supplementary Figure 57. ^{1}H-^{13}C HMQC spectrum of C_{60}@$\mathbf{1}_4$ in $CDCl_3$.

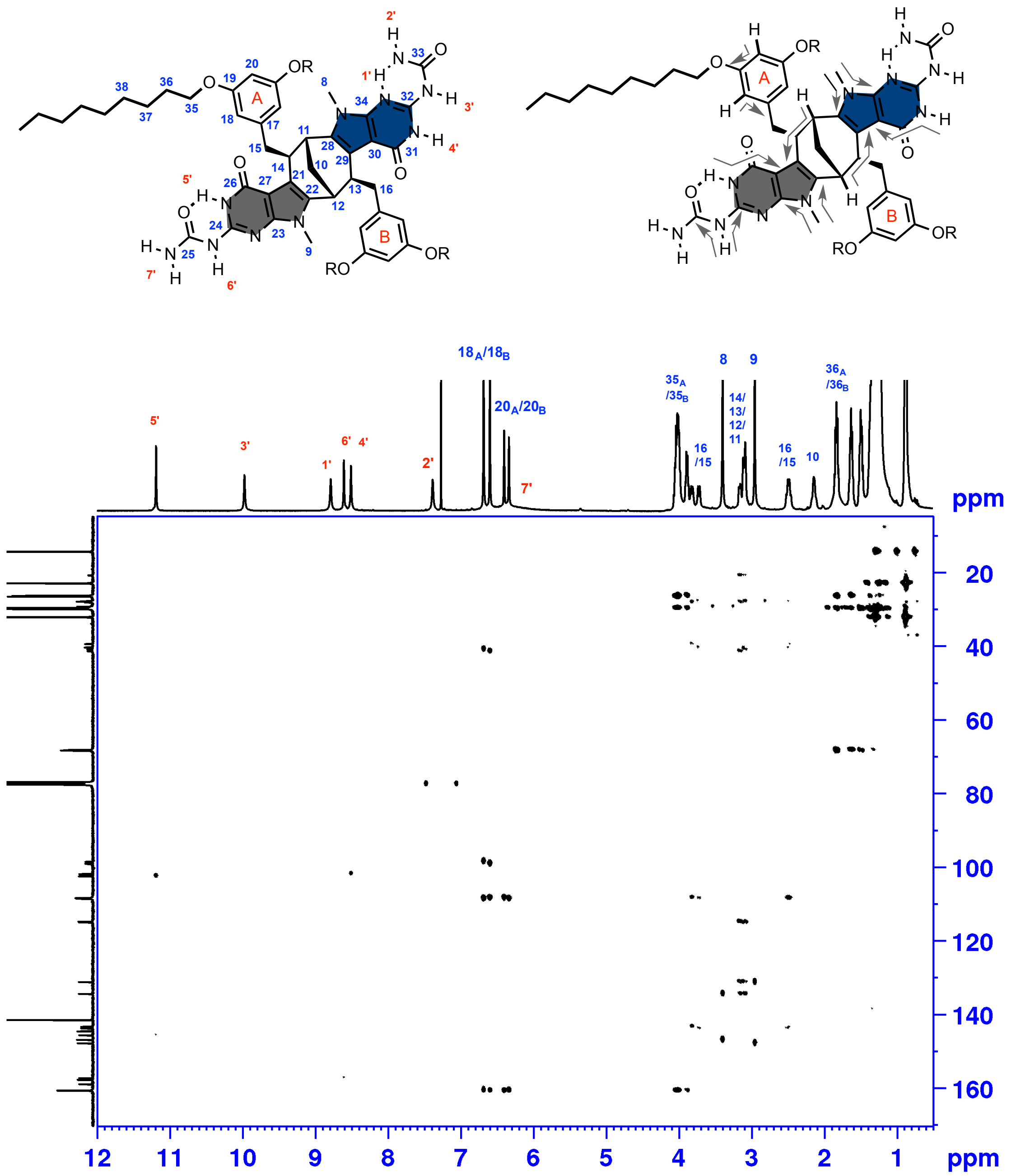

Supplementary Figure 58. HMBC spectrum of C_{60}@**1**$_4$ in $CDCl_3$. The important 2J and 3J correlations used for the assignment of ^{13}C resonances are marked with grey arrows.

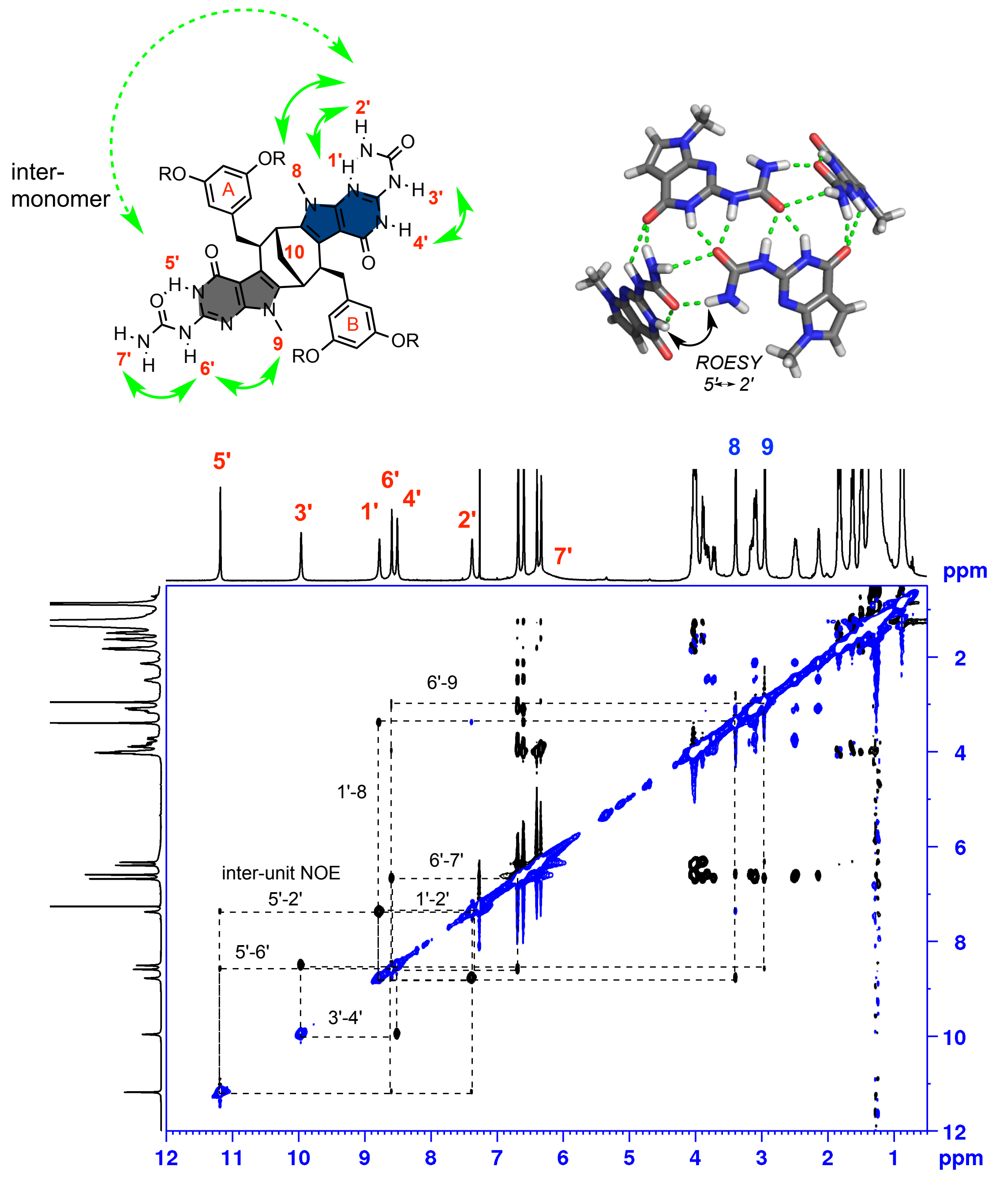

Supplementary Figure 59. ROESY spectrum of **1** in $CDCl_3$. The important intermolecular NOEs are shown in green arrows and on the H-bonding interface of C_{60}@$\mathbf{1}_4$ molecular model.

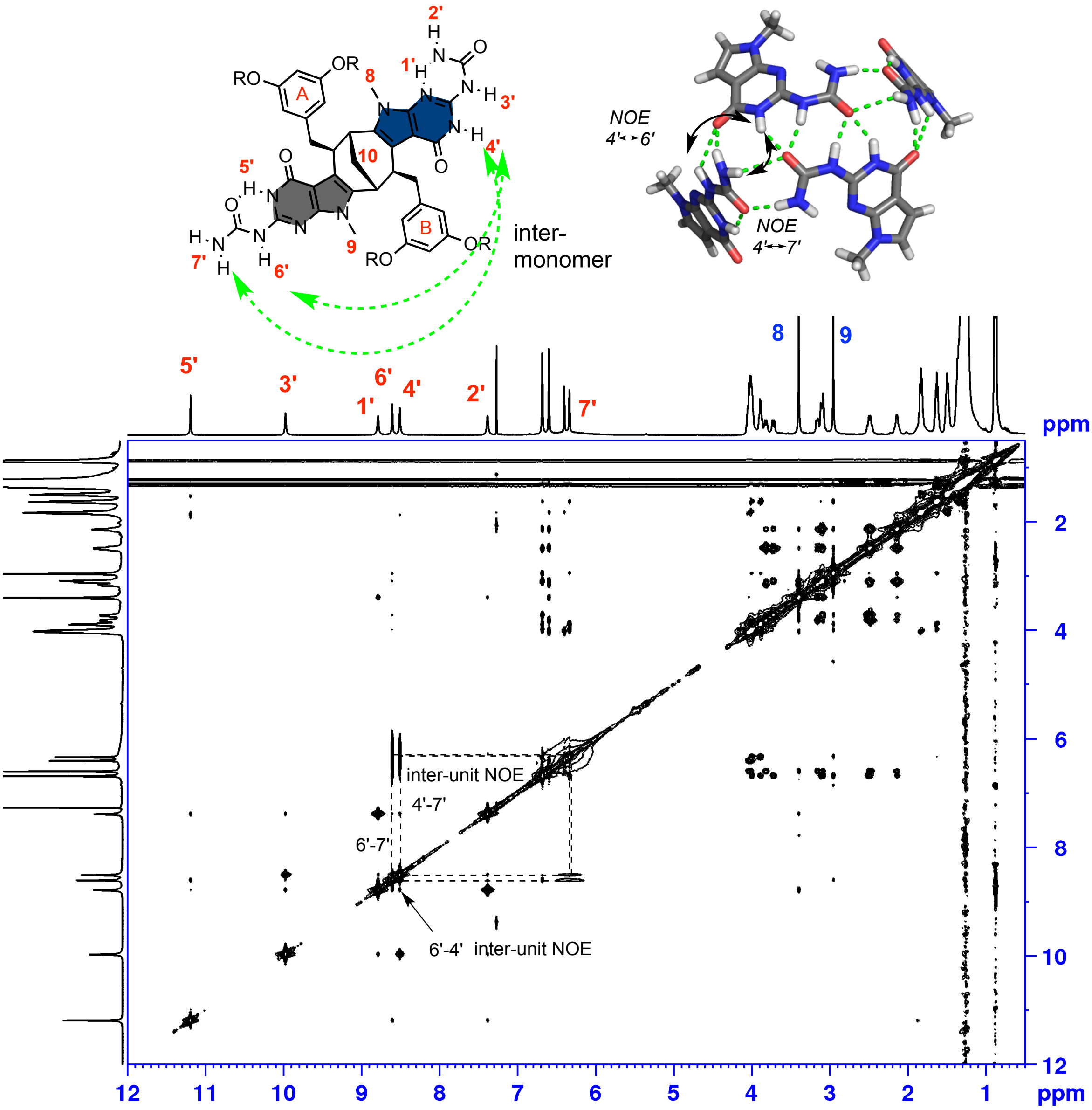

Supplementary Figure 60. NOESY spectrum of **1** in $CDCl_3$. The important intermolecular NOEs are shown in green arrows and on the H-bonding interface of C_{60}@**1**$_4$ molecular model. The broad overlapping resonance of proton 7′ is revealed by the NOE interaction with protons 4′.

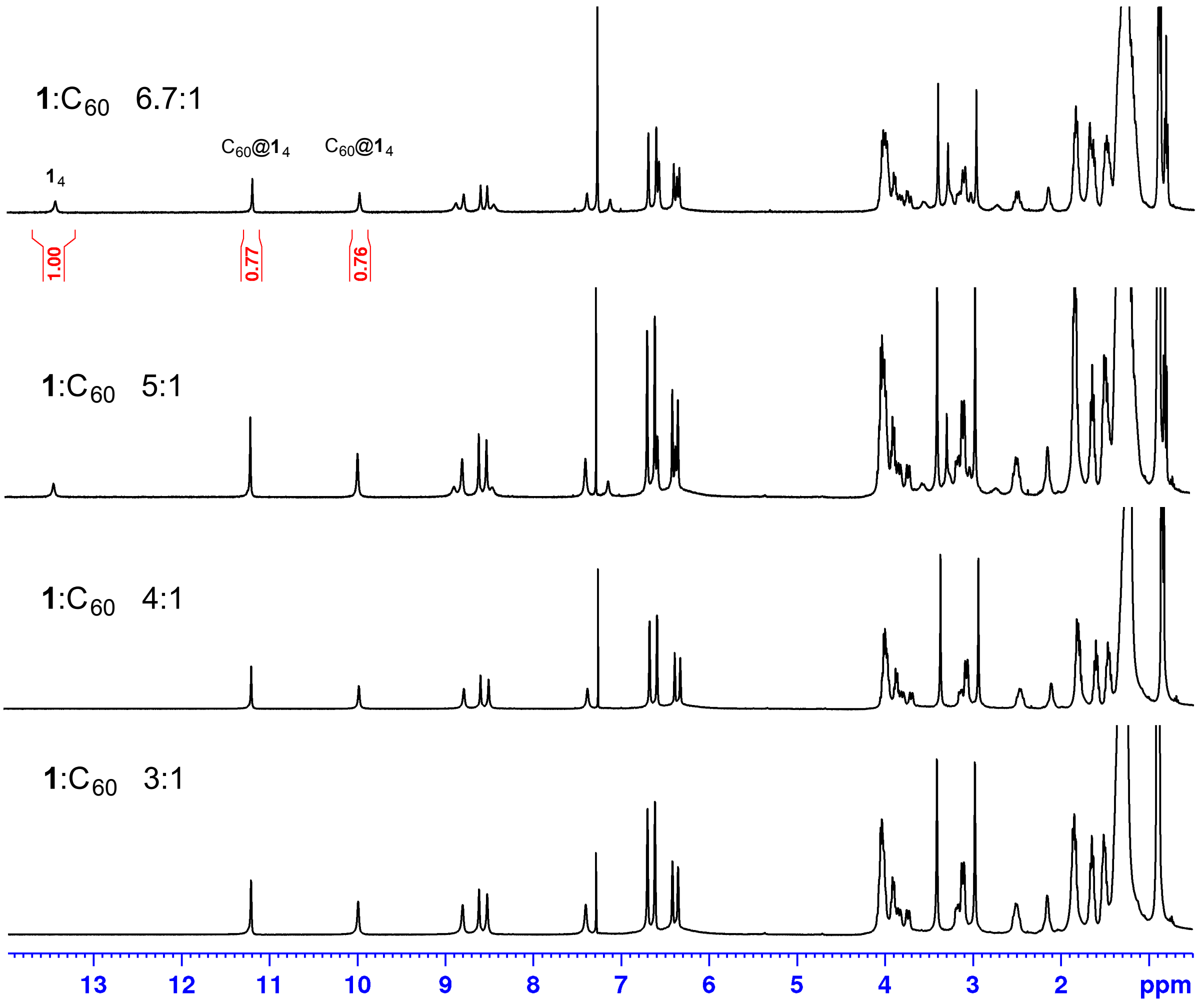

Supplementary Figure 61. **^{1}H NMR titration of C_{60} with 1 in $CDCl_3$. Sample preparation: to a carefully weighted mixture of 1 and C_{60} $CDCl_3$ was added and the mixture was stirred for one week at rt** ^{1}H NMR titration experiment showed that resonances of the free **1**$_4$ disappears when the fraction of C_{60} reaches 0.25 equiv. Mixing of C_{60} with an excess of **1**$_4$ and integrating the resulting ^{1}H NMR spectra confirmed the 4:1 stoichiometry of the inclusion complex. For instance, ^{1}H NMR spectrum of 6.7:1 – **1**:C_{60} mixture (top) gave the integral ratio of the free **1** and C_{60}@**1**$_4$ equal to 1/0.77 = 1.3 which translated into the ratio **1**:C_{60} = 6.6 (after taking into account the C_2-symmetry of **1** in free **1**$_4$). The good agreement between the actual ratio and the one calculated from monomer-tetrameric complex equilibrium confirms the 1:4 stoichiometry of the inclusion complex.

.

Molecular modeling. The geometry of monomer **1** having two different conformers of PUPY unit was first calculated at semi-empirical quantum chemistry level of theory (AM1 within Spartan 10 [15]). The optimized monomers were then used to construct D_2-symmetric tetramer with an alternating arrangement of H-bonded conformers at the poles of capsule-like aggregate with C_{60} molecule inside. Solubilizing groups were replaced with H to save computational time. However, simple geometry optimization of complete tetrameric structure showed that solubilizing groups do not interact significantly and can be easily accommodated around the capsule without significant steric interference. All attempts to assemble analogous supramolecular complex with C_{70} failed.

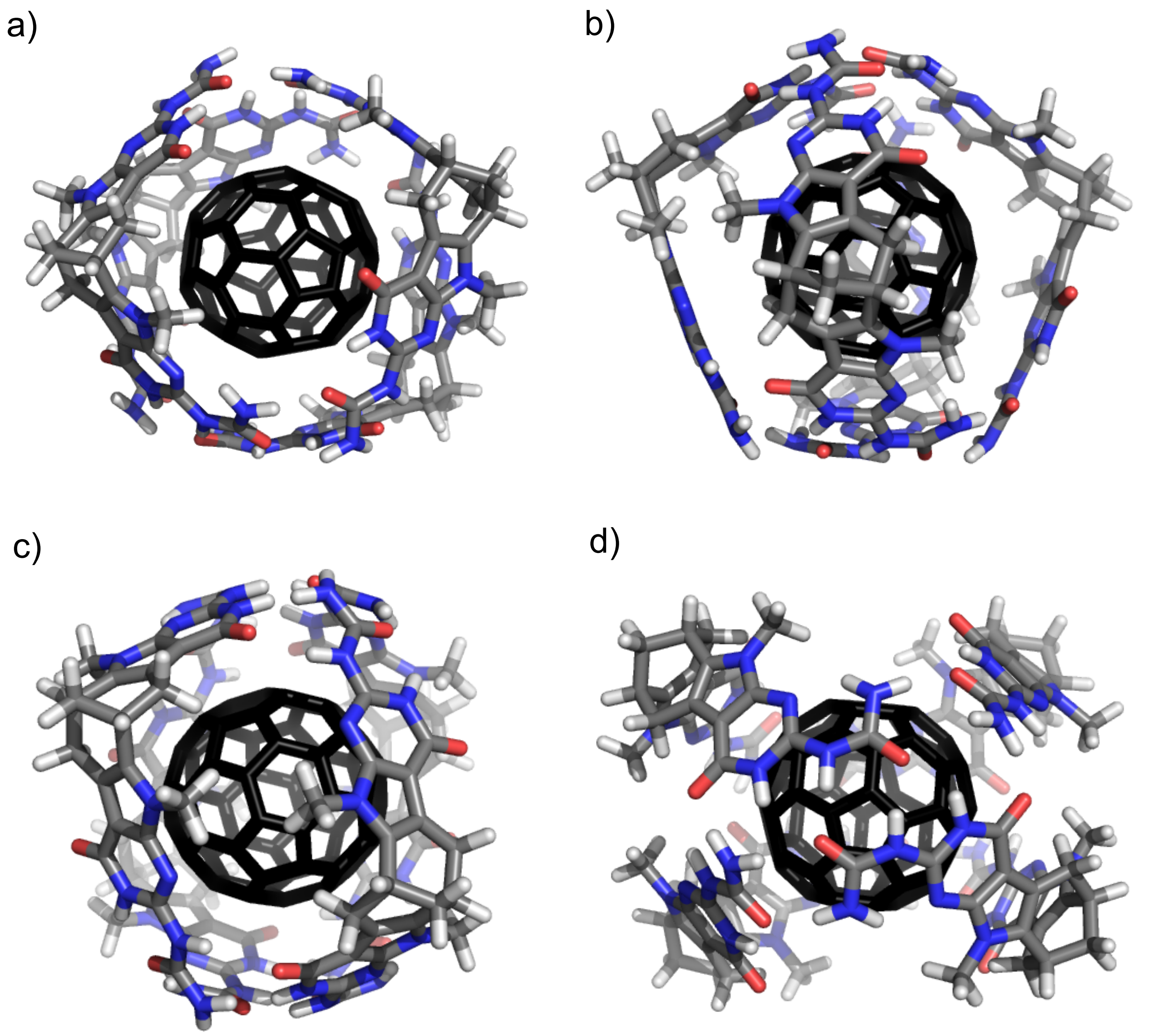

Supplementary Figure 62. Sideview (a-c) and topview (d) images of the optimized structure of C_{60}@$\mathbf{1}_4$.

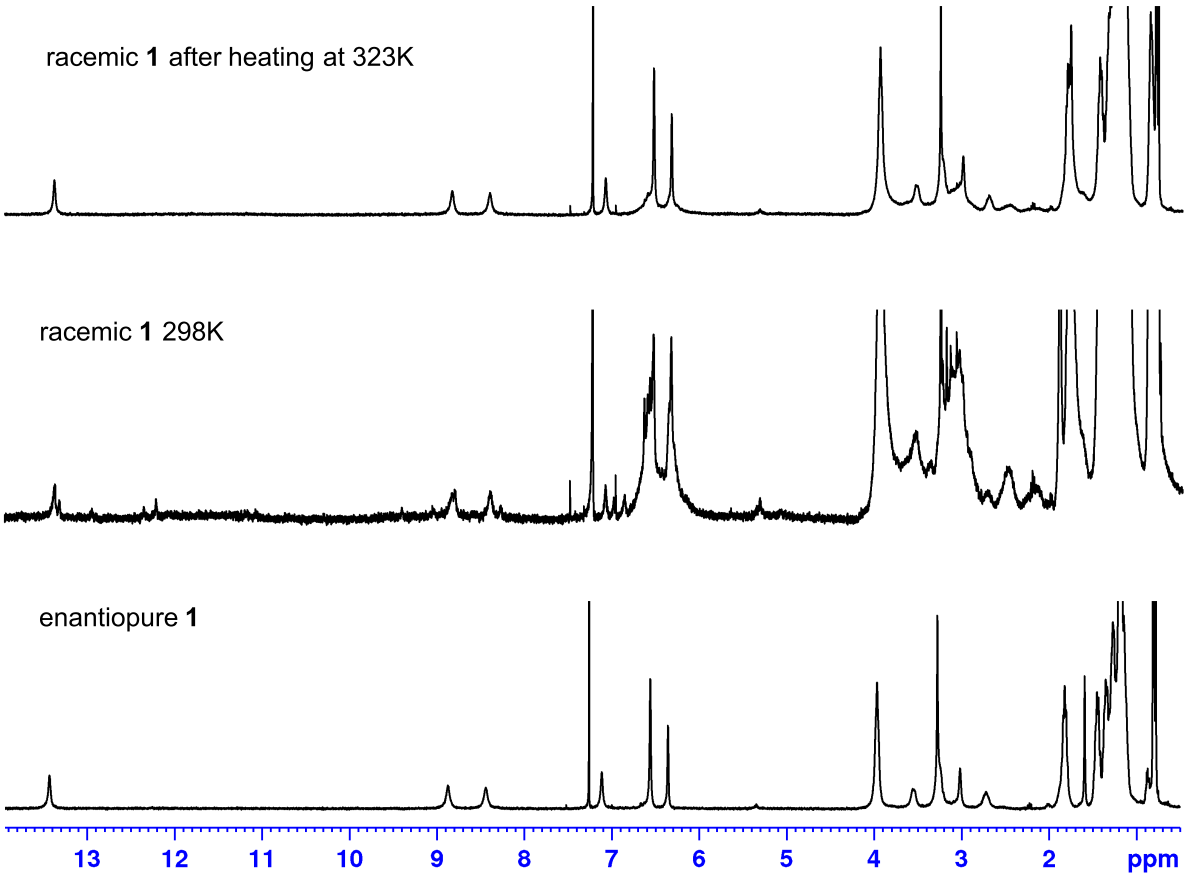

Supplementary Figure 63. ^{1}H NMR spectra of enantiopure (a) and racemic **1** (b,c). As dissolved, racemic **1** exists mainly as a polymer as evidenced by the small integral values of NH protons and broadness of resonances. The polymer is most likely a zig-zag 2H-bonded heterochiral tape. After heating to 323K, the kinetic heterochiral polymer rearranges into thermodynamic homochiral cyclic tetramer.

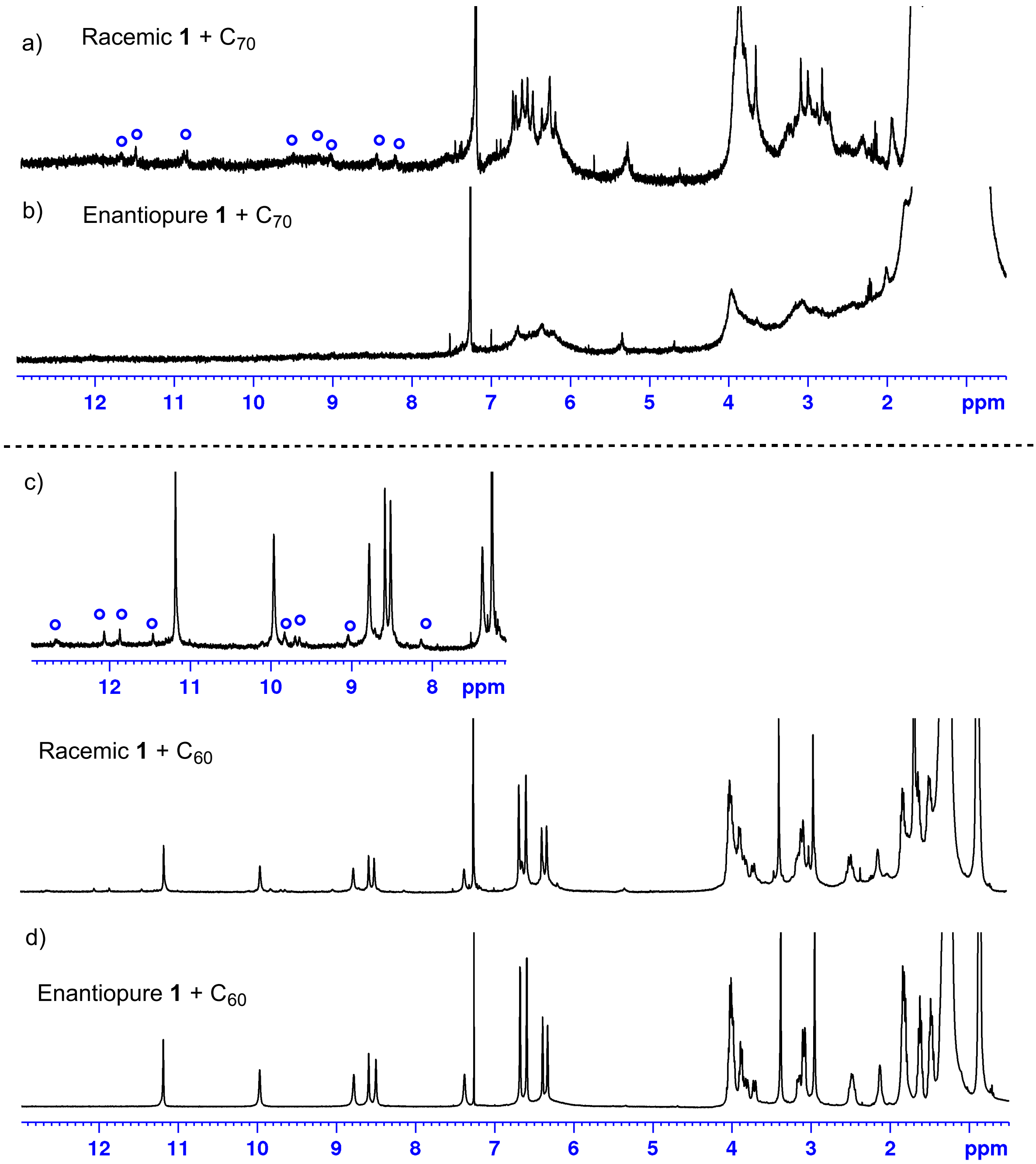

Supplementary Figure 64. Comparison of ^1H NMR spectra of the mixture of enantiopure and racemic **1** with C_{70} (a,b) and C_{60} (c,d). Blue circles indicate unknown supramolecular impurities formed from racemic **1**.

Solid-liquid extraction of C_{60}/C_{70} mixture with 1. The amounts of C_{60} and C_{70} extracted from solid C_{60}/C_{70} samples were determined using Agilent 1100 Series HPLC system equipped with Waters Symmetry® C18, 3.5 μm, 4.6x150 mm column (mobile phase: Toluene: MeOH: ACN 60:20:20 (v/v/v); flow rate: 1 mL/min; injection volume: 10 μL; detector: 330 nm).

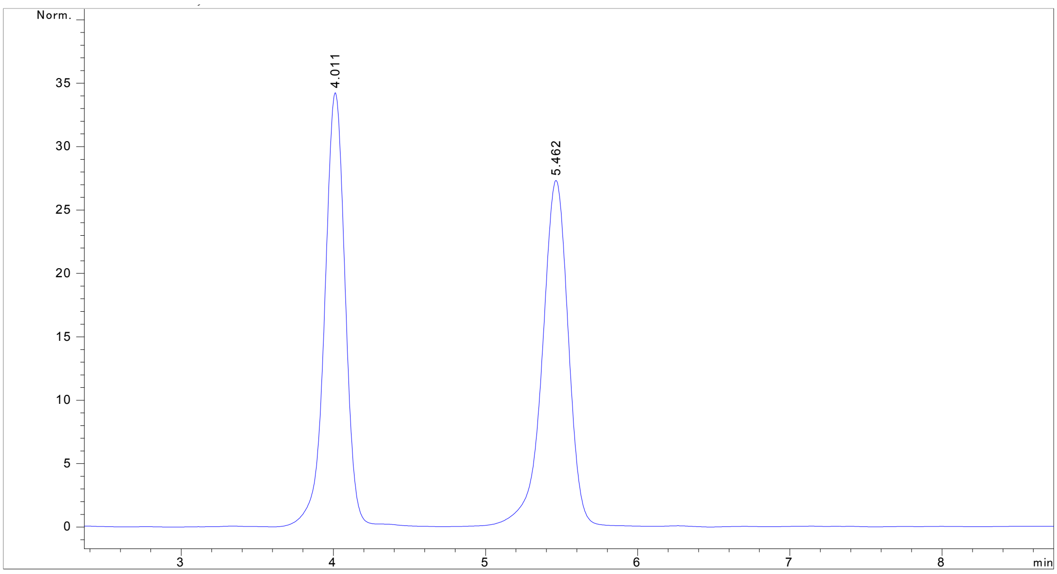

Supplementary Figure 65. Chromatogram of C_{60}/C_{70} mixture (t_{C60} = 4.011 min; t_{C70} = 5.462 min).

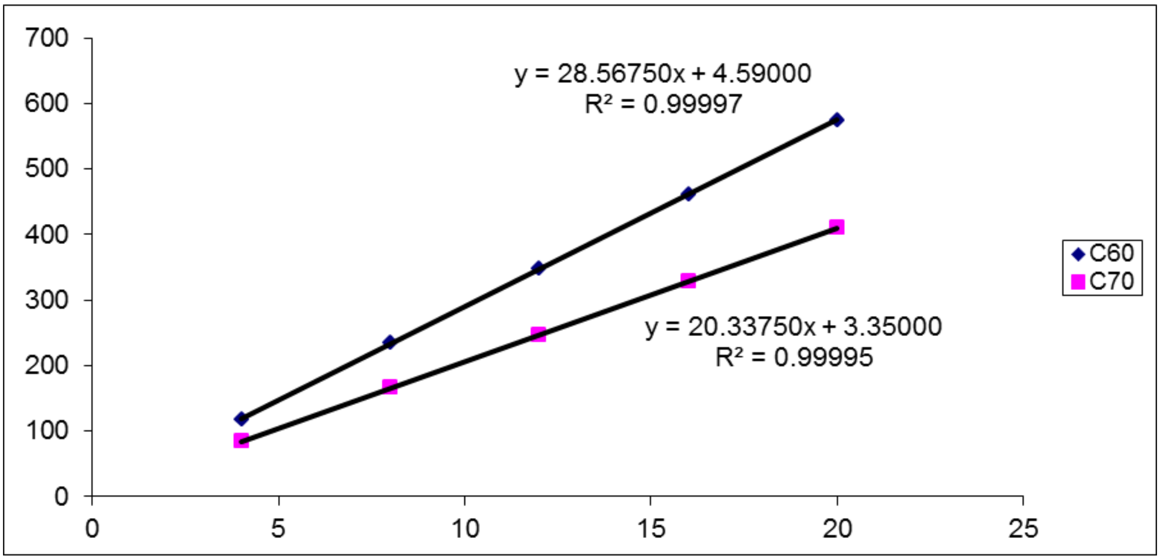

Supplementary Figure 66. Standard curve for C_{60} and C_{70}.

Supplementary Table 2. Liquid-solid extraction of C_{60}/C_{70} with **1**. The solid-liquid extraction of fullerenes with 1 showed no significant selectivity and the affinities of 1_n and 1_4 toward C_{70} and C_{60}, respectively, were very similar. The large increase of the solubility of fullerenes in $CDCl_3$ was observed in the presence of 1.

Sample[b]	C_{60}/C_{70}/**1**$_4$ (solid, mol/mol/mol)[a]	C_{60} (solid, mol%)[a]	C_{70} (solid, mol%)[a]	C_{60} (solution, mol%)[e]	C_{70} (solution, mol%)[e]
1	1.00/4.21/0.26	19.2	80.8	17.1	82.9
2	1.00/0.70/0.11	59.0	41.0	63.7	36.3
3	1.00/1.10/0.15	47.6	52.4	54.6	45.4
3B[c]				54.5	45.5
3C[d]				55.3	44.7
4	1.00/0.28/0.045	78.1	21.9	92.5	7.5
5	1.00/3.00/0.14	25.0	75.0	29.1	70.9
6	1.00/0.98/0.061	50.5	49.5	53.6	46.4
blank	1.00/1.06/0	48.5	51.5	54.8	45.2

[a] The preparation of sample: a mixture of solid C_{60}, C_{70} and **1** was put into a small vial followed by 0.8 mL of dried $CDCl_3$. The suspension was stirred at rt for at least one week. Then, it was filtered by syringe filter for removing the excess of solid fullerene. For standard solution, known amount of C_{60} or C_{70} were stirred in $CDCl_3$ until full dissolution and then diluted to obtain required concentration [b] The preparation of sample for HPLC: 50 μl of filtered solution, were diluted with 950 μl of toluene and then 10 μl TFA were added; [c] TFA was added before dilution; [d] Additional 10 μl of TFA were added to 3B; [e] The data were corrected by subtracting HPLC area of the blank sample.

Self-sorting experiments. *Sample preparation*. A mixture of monomers 1 (10.0 mg, 7.78 μmol) and 9 (10.0 mg, 7.78 μmol) were dissolved in $CDCl_3$ (600 μL) in NMR tube. After 24 hr, ^1H NMR spectrum was acquired and then, C_{60} (1.4 mg, 1.95 μmol) was added as a solid. The mixture was kept at room temperature until homogenous solution is obtained and no further changes in ^1H NMR spectrum was observed. The solution was evaporated and dried *in vacuo* before toluene-d_8 was added. After 24 hr, the ^1H NMR spectrum was recorded and the solvent was again removed *in vacuo*. Redissolving the sample in $CDCl_3$ and aging the solution for 48 hr resulted in the recovery of the original spectrum.

Supplementary References

[1] Orentas, E., Bagdžiūnas, G., Berg, U., Žilinskas, A. & Butkus, E. Enantiospecific synthesis and chiroptical properties of bicyclic enones. *Eur. J. Org. Chem.* **25**, 4251–4256 (2007).

[2] Hevener, K. E. et al. Structural studies of pterin-based inhibitors of dihydropteroate synthase. *J. Med. Chem.* **53**, 166–177 (2010).

[3] Wallentin, C.-J., Orentas, E., Butkus, E. & Wärnmark, K. Baker's yeast for sweet dough enables large-scale synthesis of enantiomerically pure bicyclo[3.3.1]nonane-2,6-dione. *Synthesis* 864–867 (2009).

[4] Hung, N. C. & Bisagni, E. Autre voie d'acces aux 5h-pyrido ¦3',4':4,5¦pyrrolo ¦3,2-c¦pyridines et leur transformation en derives n-5 et n-8 substitues. *Tetrahedron* **42**, 2303– 2309 (1986).

[5] Shi, Q. et al. Composition- and size-controlled cyclic self-assembly by solvent- and C_{60}-responsive self-sorting. *J. Am. Chem. Soc.* **135**, 15263–15268 (2013).

[6] Park, T., Todd, E. M., Nakashima, S. & Zimmerman, S. C. A quadruply hydrogen bonded heterocomplex displaying high-fidelity recognition. *J. Am. Chem. Soc.* **127**, 18133–18142 (2005).

[7] (a) Dodgson, K. & Semlyen, J. A. Studies of cyclic and linear poly(dimethylsiloxanes): 1. Limiting viscosity number–molecular weight relationships. *Polymer* **18**, 1265–1268 (1977). (b) Kricheldorf, H. R. Cyclic polymers: synthetic strategies and physical properties. *J. Polym. Sci. Polym. Chem.* **48**, 251–284 (2010). (c) Semlyen, J. A. Cyclic polymers 2nd edn (Kluwer Academic, 2000). (d) Fukatsu, M. & Kurata, M. Hydrodynamic properties of flexible ring macromolecules. *J. Chem. Phys.* **44**, 4539–4545 (1966).

[8] Dobrowolski, J. Cz., Jamróz, M. H. & Mazurek, A. P. Infrared study on the double hydrogen bond between the urea molecule and halogenated aliphatic hydrocarbon solvents. *Vib. Spectrosc.* **8**, 53–60 (1994).

[9] Keuleers, R., Desseyn, H. O., Rousseau, B. & Van Alsenoy, C. Vibrational analysis of urea. *J. Phys. Chem. A* **103**, 4621–4630 (1999).

[10] Rousseau, B., Keuleers, R., Desseyn, H. O., Geise, H. J. & Van Alsenoy, C. Solids modeled by ab-initio crystal field methods. effects of intermolecular interactions on the vibrational spectrum of urea. *Chem. Phys. Lett.* **302**, 55–59 (1999).

[11] Rousseau, B., Van Alsenoy, C., Keuleers, B. & Desseyn, H. O. Solids modeled by ab-initio crystal field methods. part 17. Study of the structure and vibrational spectrum of urea in the gas phase and in its $P42_1m$ crystal phase. *J. Phys. Chem. A* **102**, 6540–6548 (1998).

[12] Grdadolnik, J. & Maréchal, Y. Urea and urea-water solutions – an infrared study. *J. Mol. Struct*. **615**, 177–189 (2002).
[13] Perkins, S. L., Painter, P. & Colina, C. M. Molecular dynamics simulations and vibrational analysis of an ionic liquid analogue. *J. Phys. Chem. B* **117**, 10250–10260 (2013).
[14] Jung, Y. M., Czarnik-Matusewicz, B. & Kim, S. B. Characterization of concentration-dependent infrared spectral variations of urea aqueous solutions by principal component analysis and two-dimensional correlation spectroscopy. *J. Phys. Chem. B* **108**, 13008–13014 (2004).
[15] Spartan'10, Wavefunction, Inc. Irvine, CA
